# Neuronal network activity and connectivity are impaired in a conditional knockout mouse model with PCDH19 mosaic expression

**DOI:** 10.1038/s41380-023-02022-1

**Published:** 2023-03-30

**Authors:** Giorgia Giansante, Sara Mazzoleni, Antonio G. Zippo, Luisa Ponzoni, Anna Ghilardi, Greta Maiellano, Elly Lewerissa, Eline van Hugte, Nael Nadif Kasri, Maura Francolini, Mariaelvina Sala, Luca Murru, Silvia Bassani, Maria Passafaro

**Affiliations:** 1 Institute of Neuroscience, CNR, 20854 Vedano al Lambro, Italy; 2 Department of Medical Biotechnology and Translational Medicine, Università degli Studi di Milano, 20129 Milano, Italy; 3 NeuroMI Milan Center for Neuroscience, University of Milano-Bicocca, 20126 Milano, Italy; 4 Radboud University Nijmegen Medical Centre, Donders Institute for Brain, Cognition, and Behaviour, Department of Human Genetics, Department of Human Genetics Cognitive Neuroscience, Nijmegen, Netherlands

**Keywords:** Autism spectrum disorders, Schizophrenia, Neuroscience

## Abstract

Mutations in *PCDH19* gene, which encodes protocadherin-19 (PCDH19), cause Developmental and Epileptic Encephalopathy 9 (DEE9). Heterogeneous loss of PCDH19 expression in neurons is considered a key determinant of the disorder; however, how PCDH19 mosaic expression affects neuronal network activity and circuits is largely unclear. Here, we show that the hippocampus of *Pcdh19* mosaic mice is characterized by structural and functional synaptic defects and by the presence of PCDH19-negative hyperexcitable neurons. Furthermore, global reduction of network firing rate and increased neuronal synchronization have been observed in different limbic system areas. Finally, network activity analysis in freely behaving mice revealed a decrease in excitatory/inhibitory ratio and functional hyperconnectivity within the limbic system of *Pcdh19* mosaic mice. Altogether, these results indicate that altered PCDH19 expression profoundly affects circuit wiring and functioning, and provide new key to interpret DEE9 pathogenesis.

## Introduction

Mutations in the X-linked gene *PCDH19* (Xq22.1) cause a severe neurodevelopmental disorder known as Developmental and Epileptic Encephalopathy 9 (DEE9, OMIM # 300088) [1]. DEE9 is characterized by early-onset epilepsy and very heterogeneous spectrum of neuropsychiatric symptoms, including intellectual disability of various degrees, autism spectrum disorder (ASD), and late-onset schizophrenia [1–5]. Typically, DEE9 affects females with heterozygous *PCDH19* mutations, whereas hemizygous male carriers are seizure-free [2, 3, 6, 7]. Due to random chromosome X inactivation, female patients are expected to show mosaic expression of *PCDH19* in the brain, with some cells expressing the wild-type (WT) *PCDH19* and others not expressing it or expressing the mutant allele. It has been proposed that *PCDH19* mosaicism might affect cell-cell communication, according to the cellular interference hypothesis [8]. The identification of rare DEE9 male patients with postzygotic *PCDH19* mutations, another possible cause of *PCDH19* mosaic expression, reinforced this hypothesis [6, 9–12].

*PCDH19* encodes protocadherin-19 (PCDH19), an adhesion molecule of the cadherin superfamily [13]. PCDH19 is widely expressed in the central nervous system, especially in the limbic system [14–18]. PCDH19 expression starts during embryonic development and persists in adulthood, with a peak in the first postnatal period [19–22], when intense remodeling of neuronal wiring and circuit assembly occurs [23]. The adhesive properties [24] and synaptic expression of PCDH19 [21, 25] make this protein well equipped for neuronal circuit organization, as suggested by its involvement in neuronal migration, sorting and clustering [21, 26–28]. At synapses, PCDH19 has been shown to regulate synaptic transmission by forming adhesive units with NCAD [29–31] and through its interaction with type A γ-aminobutyric acid receptor (GABA_A_R) [21, 32]. Despite this, the functional consequences of PCDH19 altered expression and the mechanisms underlying DEE9 pathogenesis at network level remain to be clarified.

Here, we took advantage of the *Pcdh19* conditional knockout (cKO) mouse model to study neuronal activity and functional connectivity within the limbic system. We found that the hippocampus of *Pcdh19* cKO mice is characterized by structural and functional synaptic defects and by the presence of hyperexcitable neurons. Overall, limbic system network activity was reduced and highly synchronized, with a decrease of excitatory/inhibitory ratio and an increase of functional connectivity. Altogether, these results reveal a critical role of PCDH19 in tuning neuronal network activity and connectivity.

## Materials and methods

### Mice

*Pcdh19* floxed mice (C57BL/6 background) were generated using Cre-loxP technology by flanking exon 3 with loxP sites (see Supplementary Methods for details). *Pcdh19* floxed female mice (*Pcdh19*^*fl/fl*^) were crossed with Syn1-Cre male mice (Strain #003966, The Jackson Laboratory) to obtain *Pcdh19* cKO. Mice of both sexes were used, as indicated. For genotyping, the following PCR primers were used (5’-3’): *Pcdh19* Fw, TCTCCCCCATAGGCTCAACTTTCC; *Pcdh19* Rev, AGTGCCTTTAGGATTCCGAACCACAGG; Cre Fw, CCAGCACCAAAGGCGGGC; Cre Rev, TGCATCGACCGGTAATGCAG. *Pcdh19* floxed mice (*Pcdh19*^*fl/fl*^ or *Pcdh19*^*fl/fl*^ + *Pcdh19*^*fl/y*^, as indicated) at postnatal day (P)0 were injected intracerebroventricularly (ICV) as previously described [33–35] (see Supplementary Methods for details). Animal care and all experimental procedures involving animals were performed in accordance with the CNR licensing and were approved by the Italian Ministry of Health (authorization n. 534/2017-PR, 239/2018-PR, 530/2019-PR, 708/2019-PR, 316/2018-PR, 384/2021-PR).

### Primary mouse neurons

Dissociated cortical and hippocampal neurons were obtained from *Pcdh19* floxed mice of either sex (*Pcdh19*^*fl/fl*^ and *Pcdh19*^*fl/y*^). P0 pups were sacrificed and brain tissue was disaggregated for 30 min at 37 °C with Trypsin (0.25 %, Gibco) in Hanks’ Balanced Salt Solution (1X HBSS, Gibco) supplemented with 0.5 M MgSO_4_, 1 M HEPES pH 7.4, 1% Penicillin-Streptomycin (Gibco). Neurobasal A (Gibco) with 10% Fetal Bovine Serum (FBS) was added and, after centrifugation (3 min, 1500 rpm), the pellet was resuspended in HBSS at 37 °C with 10% FBS and 1.25% DNaseI (Merck). Tissues were mechanically disaggregated and incubated 5 min at room temperature (RT). The supernatant was centrifuged (6 min, 1000 rpm) and the pellet was resuspended in Neurobasal A medium supplemented with 1% L-glutamine, 4% B27, 1% Glucose, 1% Penicillin-Streptomycin (Gibco). Neurons were plated on poly-D-lysine coated coverslips in 12-multiwells plates at a density of 10^5^/well for immunofluorescence (IF) experiments and of 1.2 × 10^5^/well for RT-PCR and Western blots. Neurons were maintained in complete medium at 37 °C, 5% CO_2_. Neurons were infected at days in vitro (DIV)0 with adeno associated viral (AAV) particles expressing GFP-Cre (AAV9.hSyn.HI.eGFP-Cre.WPRE.SV40, Penn Vector, USA; 5.5 × 10^8^ particles for multi electrode array (MEA) and 5.5 × 10^9^ particles for RT-PCR, IF and Western blot).

### Human-induced pluripotent stem cells (hiPSCs)-derived neurons

hiPSCs used in this study were obtained from reprogrammed control fibroblasts. hiPSCs were cultured on Matrigel (Corning, #356237) in E8 Flex (Thermo Fisher) supplemented with primocin (0.1 μg/ml, InvivoGen), puromycin (0.5 μg/ml) and G418 (50 μg/ml) at 37 °C/5% CO_2_. Medium was refreshed every 2–3 days and cells were passaged twice per week using an enzyme-free reagent (ReLeSR, Stem Cell Technologies). hiPSCs were differentiated using the Neurogenin 2 (Ngn2)-protocol as previously described [36–38]. Briefly, hiPSCs were directly differentiated into excitatory cortical layer 2/3 neurons by overexpressing Ngn2 upon doxycycline treatment. Neuronal maturation was supported by rat astrocytes, which were added to the culture in a 1:1 ratio two days after hiPSC plating. At DIV3 the medium was changed to Neurobasal medium (Cat#21103049, Thermo Fisher) supplemented with B27 (Cat#17504001, Thermo Fisher), glutaMAX (Cat#35050061, Thermo Fisher), primocin (0.1 µg/ml), NT3 (10 ng/ml) (Cat#PHC7036, Thermo Fisher), BDNF (10 ng/ml), and doxycycline (4 µg/ml). Cytosine β-D-arabinofuranoside (2 µM) (Cat#C1768, Sigma-Aldrich) was added to remove any proliferating cell from the culture at DIV3. From DIV6 onwards half of the medium was refreshed every other day. From DIV10 onwards the medium was additionally supplemented with 2.5% FBS to support astrocytes viability. Neuronal cultures were kept through the whole differentiation process at 37 °C/5%CO_2_. Lentiviral vectors encoding *PCDH19* shRNA or a scrambled shRNA [21] were prepared as previously described [38]. hiPSCs-derived neurons were infected at DIV7 and their activity recorded at DIV35.

### Pentylenetetrazol (PTZ)-induced seizures and behavioral tests

*Pcdh19* cKO females (*Pcdh19*^*fl/x*^ Syn1-Cre) and control littermates (*Pcdh19*^*fl/x*^) were housed in groups of mixed genotypes and kept in 12-hours light/dark cycle with food and water ad libitum. For growth curve, mice were weighted weekly starting from the 3^rd^ postnatal week to the 3^rd^ month. All the behavioral experiments followed the ARRIVE guidelines and behavioral scoring was performed on a blind basis. Mice were habituated to the tests room at a constant temperature (22 °C ± 2 °C) for 45 min before tests. Behavioral tests were conducted on adult (>P90) mice according to standard procedures, as described in Supplementary Methods. Seizure susceptibility was assessed in P26-28 mice. *Pcdh19* cKO mice and controls, after 10 min habituation in the experimental cage, received an intraperitoneal injection of PTZ (40 mg/Kg) and were monitored for 30 min. Seizures were classified according to a modified version of the Racine scale (Stage 0: normal behavior; Stage 1: immobility/hypoactivity; Stage 2: partial clonus (head bobbing, forelimb clonus); Stage 3: mild whole body clonus; Stage 4: severe whole body clonus; Stage 5: tonic-clonic seizures [39, 40].

### RT-PCR

mRNA was extracted from primary neurons at DIV10 and from brain tissues of *Pcdh19* cKO mice (*Pcdh19*^*fl/x*^ Syn1-Cre, *Pcdh19*^*fl/y*^ Syn1-Cre) and controls (*Pcdh19*^*fl/x*^, *Pcdh19*^*fl/y*^) at P90 using RNeasy mini kit (Qiagen) and Nucleozol Reagent Kit (Macherey Nagel), respectively. mRNA was retrotranscribed into cDNA using SuperScript VILO cDNA Synthesis Kit (Thermo Fisher). *Pcdh19* and the housekeeping gene *α-actin* were amplified by using SYBR Green PCR Master Mix (Applied Biosytems) in an Applied Biosystems 7000 thermocycler. The following primers were used for RT-PCR (5’-3’): *Pcdh19* exon (E) 1-2 Fw, ATCCGGACCTACAATTGCAG; *Pcdh19* E1-2 Rev, ATAAAACAGCCGAGGAGACAAG; *Pcdh19* E4-5 Fw, GCCGTGCCCATTTAATCA; *Pcdh19* E4-5 Rev, TTCACAGCAGTATCGCAGTACAG; *Pcdh19* E5-6 Fw, TGGGATCTCAGATGCCTG; *Pcdh19* E5-6 Rev, CCAGCATCTATCAGAGTGGC; *α-actin* Fw, AGATGACCCAGATCATGTTTGAGA; *α-actin* Rev, CCTCGTAGATGGGCACAGTGT. For RT-PCR on brain tissue *Pcdh19* E1-2 For and Rev primers were used. Data were analyzed though ABI PRISM 7000 software (Applied Biosystems).

### Western blot

Western blots were done on cultured neurons at DIV10 and on brain tissues from >P90 mice: *Pcdh19* cKO mice (*Pcdh19*^*fl/x*^ Syn1-Cre, *Pcdh19*^*fl/y*^ Syn1-Cre) and controls (*Pcdh19*^*fl/x*^, *Pcdh19*^*fl/y*^); *Pcdh19* floxed mice (*Pcdh19*^*fl/fl*^, *Pcdh19*^*fl/y*^) injected with GFP-2A-Cre adeno-associated viruses (AAVs) and controls (C57BL/6 WT mice). Cells and tissues underwent homogenization in modified Radio Immunoprecipitation Assay (RIPA) buffer (50 mM Tris-HCl, 150 mM NaCl, 1 mM EDTA, 1% NP-40, 1% Triton X100, pH 7.4 and protease inhibitors). Homogenates were mixed with Sample Buffer (SB 3X: 22,2% glycerol, 18.7 mM TRIS pH 6.8, 10% SDS, 3.55% β-mercaptoethanol, Bromophenol Blue) and underwent SDS-PAGE. Proteins were transferred to nitrocellulose membranes (0.2 µm, Amersham GE Healthcare). Membranes were blocked in 5% skim milk in Tris-Buffered Saline (TBS) for 1 hour at RT and then washed in TBS with 0.1% Tween 20 detergent (TBST) for 5 min at RT. Membranes were incubated overnight at 4 °C with the following primary antibodies prepared in TBST plus 5% skim milk: rabbit anti-PCDH19 1:20000, Bethyl Laboratories, Cat#A304-468A; rabbit anti-GFP 1:2500, MBL International Corporation, Cat#598; mouse anti-alpha tubulin 1:40000, Sigma-Aldrich, Cat#T5168; rabbit anti-GAPDH 1:2000, Santa Cruz Biotechnology, Cat#sc-25778. After washing with TBST and TBS, membranes were incubated with secondary antibodies for 1 hour at RT (anti-mouse IRDye 680 nm and anti-rabbit 800 nm, 1:7500, Li-Cor, Cat#92668070 and Cat#92632211; peroxidase-conjugated anti-rabbit 1:20000, Jackson ImmunoResearch Labs, Cat#111-035-00; peroxidase-conjugated anti-mouse 1:2000, GE Healthcare, Cat#NA931). Proteins were detected by using the Odyssey CLx (Li-Cor) or ChemiDoc (Bio-Rad) detector systems and quantified by Image Studio software.

### IF and image analysis

Neuron at DIV7 or DIV11 were fixed with 4% paraformaldehyde (PFA) and 4% sucrose for 10 min at RT. Neurons were incubated with primary antibodies (guinea pig anti-MAP2 1:2000, Synaptic System, Cat#188004; rabbit anti-PCDH19 1:400, Bethyl Laboratories, Cat#A304-468A; guinea pig anti-VGAT 1:600, Synaptic System, Cat#131004; mouse anti-GABA_A_R beta3 1:300, NeuroMab, Cat#75-149; guinea pig anti-VGLUT1 1:1000, Synaptic System, Cat#135304; mouse anti-PSD95 1:500, Synaptic System, Cat#124011) prepared in gelatin detergent buffer (GDB: 0.1% gelatin; 0.3% Triton X100, 16.5 mM Na_2_HPO_4_, 0.45 M NaCl, pH 7.4) for 2 hours at RT and then with secondary antibodies for 1 hour at RT (guinea pig 649 1:400, Jackson ImmunoResearch Labs, Cat#706-605-148; rabbit or mouse Alexa Fluor 555 1:400, Thermo Fisher, Cat#A21429, Cat#A21424; rabbit Alexa Fluor 488 1:400, Thermo Fisher, Cat#A11034). P30 mice (*Pcdh19*^*fl/x*^ Syn1-Cre and *Pcdh19*^*fl/x*^, *Pcdh19*^*fl/y*^ Syn1-Cre and *Pcdh19*^*fl/y*^, *Pcdh19*^*fl/fl*^ injected with AAVs, as indicated) were anesthetized with intraperitoneal injection of Zolazepam and Tiletamine (80 mg/Kg) and brains were washed through cardiac perfusion with phosphate buffer (PB, 0.1 M: 16.5 mM Na_2_HPO_4_ x 2H_2_O, 96 mM NaH_2_PO4, pH 7.4) and then fixed with 4% PFA in 0.1 M PB. 100 µm-thick coronal brain slices were cut with a vibratome (Leica, Italy) and incubated with blocking solution (0.5% Triton X100 and 10% goat serum in 0.1 M PB) for 2 hours at RT. Sections were incubated overnight at 4 °C with primary (rabbit anti-PCDH19 1:50, Thermo Fisher, Cat#PA5-55648) and secondary (rabbit Alexa Fluor 488 1:400, Thermo Fisher; rabbit Alexa Fluor 555 1:400, Thermo Fisher) antibodies for 2 hours at RT. Brain slices were incubated with DAPI (1:4000, Invitrogen, Cat#D1306) for 15 min at RT and mounted with Fluoromount™ Aqueous Mounting Medium (Merck).

For IF on brain slices showing colocalization of PCDH19 with NeuN and GAD, the brain of an anesthetized P23 female mouse (*Pcdh19*^*fl/x*^) was washed through cardiac perfusion with phosphate buffered saline (PBS, mM: 137 NaCl, 2.7 KCl, 8 Na_2_HPO_4_ x 2H_2_O, 2.3 NaH_2_PO_4_, pH 7.4) and 5% sucrose solution and then fixed with 4% PFA and 4% sucrose in PBS. Brains were post-fixed in 4% PFA and 4% sucrose overnight at 4 °C and left the following night in 30% sucrose solution at 4 °C. Brains were included in OCT and 30 µm-thick coronal brain slices were cut using a cryostat (Reichert Jung Frigocut 2700). Free-floating slices were incubated with blocking solution for 2 hours at RT and with primary antibodies at 4 °C (overnight with rabbit anti-PCDH19 1:50, Thermofisher, Cat#PA5-55648 and mouse anti-NeuN 1:500, Millipore, Cat#MAB377, for 48 h with mouse anti-GAD67 1:200, Merck, Cat#MAB5406). Slices were incubated with secondary antibodies and DAPI as before. Images were acquired with an LSM 800 confocal microscope (Carl Zeiss, Italy). For primary neurons, a 63X oil-immersion objective was used. Images were acquired at 1024 × 1024 pixel resolution and obtained from the z-projection (maximum intensity) of 3–8 sections taken at 0.75 μm intervals. For acquisition of brain slices 10X, 20X or 40X objectives (zoom 0.7-1) were used. Images were acquired at 1024 × 1024 or 512 × 512 (cortex of GFP-Cre AAVs injected mice) pixel resolution and showed as single z-stack (hippocampus of GFP-Cre AAVs injected mice) or projection (maximum intensity) of 4-14 z-stacks at 1-1.5 μm intervals. Colocalization analysis on primary neurons was performed using the ImageJ JACop plug-in [41]. Each stack was filtered by using a 3 × 3 pixel-wide median filter and the maximal fluorescence intensities were Z-projected. Images were thresholded according to their gray level histogram (mode plus six times the standard deviation) and watershedded. Pearson’s (R) coefficient, Pearson’s as a function of pixelshift (Van Steensel’s cross correlation function), and Manders’ (M1 and M2) coefficients were evaluated.

### Long-term potentiation (LTP) and paired pulse ratio (PPR) in acute brain slices

Coronal hippocampal slices (400 μm) were prepared as described [42] from P90 *Pcdh19* cKO mice (*Pcdh19*^*fl/x*^ Syn1-Cre) and controls (*Pcdh19*^*fl/x*^). Slices were incubated at RT in artificial cerebrospinal fluid (aCSF, mM: 125 NaCl, 2.5 KCl, 1.25 NaH2PO4, 1 MgCl2, 2 CaCl2, 25 glucose, and 26 NaHCO3; pH 7.3) equilibrated with 95% O_2_ and 5% CO_2_. Field excitatory postsynaptic potentials (fEPSPs) were evoked through Schaffer collateral stimulation (0.05 Hz) and recorded from the *stratum radiatum* of the hippocampal *cornu ammonis* (CA)1 using aCSF-filled capillaries. Stimulus strength was adjusted to give 50% maximal response and LTP was induced by high-frequency stimulation (HFS) (100 stimuli, 100 Hz) [43]. The analysis of glutamate release probability was performed by fEPSP PPR experiments, delivering pairs of stimuli at 50-ms intervals every 20 s (0.05 Hz). The PPR was calculated by dividing the amplitude of the second response by that of the first [43]. fEPSPs were filtered at 2 kHz and digitized at 20 kHz using Clampex 10.1 software through the patch-clamp amplifier. All the analyses were performed offline with Clampfit 10.1 software.

### Patch-clamp recordings from dentate gyrus granule cells (DGGCs)

Coronal hippocampal slices (270 μm) were prepared as described [44] from P90 *Pcdh19*^*fl/fl*^ mice injected with GFP-CRE AAVs. Slices were incubated in aCSF equilibrated with 95% O_2_ and 5% CO_2_ at 35 °C for 40 min. Patch-clamp recordings were performed from DGGCs using a K-gluconate based internal solution (mM: 126 K-gluconate, 4 NaCl, 1 EGTA, 1 MgSO4, 0.5 CaCl2, 3 ATP (magnesium salt), 0.1 GTP (sodium salt), 10 glucose, and 10 HEPES-KOH pH 7.28). DGGCs membrane properties were evaluated injecting a series of depolarizing current steps (0–150 pA, 10 pA/step, 1 s duration) to evoke action potential (AP) firing. AP frequency was correlated to the current injected in an input/output curve. The AP accommodation has been calculated by dividing the duration of the current step in ten bins and counting AP for each bin to obtain the instantaneous firing frequency [44]. Action potentials (APs) feature analysis was performed for the first AP evoked at rheobase. Voltage-dependent inward and outward currents were recorded in voltage-clamp configuration (holding potential −65 mV) and elicited by 5 mV steps (10 ms) from −75 to +85 mV. Miniature excitatory and inhibitory postsynaptic currents (mEPSCs/mIPSCs) were recorded as previously described [44]. Recordings were filtered at 2 kHz and digitized at 20 kHz using Clampex 10.1 software through the patch-clamp amplifier. All the analyses were performed offline with Clampfit 10.1 software.

### Transmission Electron Microscopy (TEM) and analysis

TEM analysis was performed on P21 *Pcdh19* cKO mice (*Pcdh19*^*fl/x*^ Syn1-Cre) and control littermates (*Pcdh19*^*fl/x*^) or P50 *Pcdh19* floxed mice (*Pcdh19*^*fl/fl*^*, Pcdh19*^*fl/y*^) ICV injected with GFP-2A-Cre AAVs (AAV9-hSYN1-eGFP-2A-iCre-WPRE, Vector Biolabs) at P0 and age-matched C57BL/6 WT mice, as indicated. Mice were anesthetized by intraperitoneal injection of Zolazepam and Tiletamine (80 mg/Kg) and transcardially perfused with 0.1% glutaraldehyde and 4% paraformaldehyde in PB 0.1 M (pH 7.4). Dissected brains were cut in coronal sections of 100 µm thickness using a vibratome (Leica VT1200S) and further fixed for 24 h at 4 °C in the same solution. Hippocampal CA1 region was manually dissected and trimmed samples were processed for TEM as previously described [42]. Briefly, samples were washed with sodium cacodylate buffer (pH 7.4) and post-fixed with 2% osmium tetroxide in sodium cacodylate buffer (pH 7.4), rinsed and *en bloc* stained with 1% uranyl acetate in bidistilled water. Samples were then dehydrated in ethanol and embedded in Epon-Spurr epoxy resin. 70 nm-thin sections were collected on copper grids and counter-stained with uranyl acetate in bidistilled water and 1% lead citrate. Grids were observed with a Talos L120C TEM (FEI) equipped with a Ceta 4k x 4k digital camera. Images were acquired at 28000x-36000x magnification for the quantitative analysis of synapses and at 8500x for the estimation of synapse density. Excitatory synapse profiles were included in the analyses when they met the following criteria: presence of at least 3 synaptic vesicles in the presynaptic terminal, defined synaptic cleft and postsynaptic density within the postsynaptic terminal. Quantitative and stereological analyses were performed using the Fiji 1.53c software, as described elsewhere [45]. For immuno-EM, sections were collected on formvar carbon-coated nickel grids and processed for GABA immunolabeling as follows: after permeabilization in TBST pH 7.6 (Tris–HCl 0.05 M, pH 7.6, with 0.9% NaCl and 0.1% Triton X-100), grids were incubated with rabbit antiserum against GABA (Sigma-Aldrich, Cat#A2051, 1:10.000 in TBST) overnight in a moist chamber at 4 °C. Grids were then washed and blocked in TBST pH 7.6. After 5 min of conditioning in TBST pH 8.2, grids were incubated for 2 hours in goat anti-rabbit IgG conjugated to 12 nm colloidal gold (Jackson ImmunoResearch Labs, Cat#111205144) diluted 1:50 in TBST pH 8.2 at RT. They were then washed twice in TBST pH 7.6, rinsed in deionized water, allowed to air-dry, and counterstained with 1% uranyl acetate and 1% lead citrate. Distributions of PSD thickness and vesicle density were assessed by Z-score analysis.

### Analysis of multielectrode array (MEA) recordings on primary neurons and brain slices

Extracellular recordings were carried out with HD-MEA system (Biocam 4096, 3Brain) equipped with 4096 electrodes chips (21 × 21 μm^2^ in size, 42 μm pitch), either Prime HD-MEA chips (primary neurons) or Arena HD-MEA chips (brain slices) (3Brain). MEA chips with cultured neurons and hippocampal slices were prepared as described in Supplementary Methods. Hippocampal brain slices were obtained from P8-10, P18-20, P60-90 *Pcdh19* cKO mice (*Pcdh19*^*fl/x*^ Syn1-Cre) and control littermates (*Pcdh19*^*fl/x*^), as indicated. Slices activity was recorded under continuous perfusion (4.5 ml/min) in Mg^2+^-free aCSF solution supplemented with the potassium channel blocker 4-Aminopyridine (100 μM, Sigma-Aldrich). Recordings were performed at full-frame resolution (7 kHz/electrode) and analyses were conducted off-line using BrainWave 4 software (3Brain). When analyzing hippocampal slices, activated regions were manually identified overlapping slice images taken with a stereomicroscope (Zeiss Stemi 305) and the pseudocolor activity map visualized on the 3Brain software. For the analysis on the entire hippocampus, electrodes with a spiking rate >0.1 spike/sec were considered and slices with less than 20 active channels were discarded. For the analysis of the mean firing rate (MFR) of hippocampal subregions (CA3, CA1 and DG), spiking threshold was not applied. For APs detection, raw data were high-pass filtered at 300 Hz (Butterworth filter, first order, analog) and a threshold (−80/−90 μVolt, depending on noise) was set by employing the Hard Threshold algorithm integrated in the Brainwave 4 software application (3Brain). Bursts events were defined by the presence of at least 3 consecutive spikes with an interspike interval (ISI) lower than 100 ms [46]. Local field potentials (LFPs) were detected by using a 150 Hz low-pass filter (Chebyshev filter, fourth order, analog) and the Hard Threshold algorithm (high/low threshold: +70/−40 μVolt; refractory period: 10 ms; maximum event duration, 1 s). Power spectra of raw events containing LFPs were calculated by using R software. The global synchronization index (SI) was calculated for each active channel as previously described, with a time series with 1-ms time bins, using the following equation:

$$\varphi \left( {{\it{t}},{\it{n}}} \right) = 2\pi {\it{n}} + 2\pi \ast \frac{{{\it{t}} - {\it{t}}({\it{n}})}}{{{\it{t}}\left( {{\it{n}} + 1} \right) - {\it{t}}({\it{n}})\prime }}$$

where *t* is the time bin, *n* is the number of the most recent spike before or at time *t*, and *t*(*n*) is the time of spike *n* [47]. SI allows identifying clusters of locally synchronized neurons, and ranges from 0 (random uncoordinated activity) to 1 (full synchronous activity).

### MEA recordings on hiPSCs-derived neurons

All recordings were performed using the 24-wells MEA system (Multichannel Systems, Germany). Recordings and analysis were performed according to previous published protocols [37, 48]. In brief, MEA devices were composed of 24 independent wells with embedded microelectrodes (i.e., 12 electrodes/well, 80 mm in diameter and spaced 300 mm apart). Spontaneous electrophysiological activity of hiPSC-derived neuronal networks was recorded for 10 min in a recording chamber that was continuously maintained at 37 °C/95% O_2_/5% CO_2_. Before recording, MEAs were allowed to acclimate for 10 min in the recording chamber. The recording was sampled at 10 kHz and filtered with a high-pass filter with a 100 Hz cut-off frequency and low-pass filter with a 3500 Hz cut-off frequency. The spike detection threshold was set at ±4.5 standard deviations. Spike detection, burst detection and network burst detection was performed by a built-in algorithm in Multiwell Analizer software (Multichannel Systems), and a custom-made MATLAB (The Mathworks, Natick, USA) code to extract parameters describing network activity. MFR was calculated as the average of the spike frequency of all channels. Burst detection settings were set at 50 ms ISI to start burst, 50 ms ISI to end burst and 100 ms inter burst interval. Network bursts were detected when at least 50% of the channels in one well displayed a synchronous burst.

### In vivo MEA recordings

Recording sessions of P37-40 freely moving *Pcdh19* cKO (*Pcdh19*^*fl/y*^ Syn1-Cre) and control mice (*Pcdh19*^*fl/y*^) started 3 days after MEA implant (Supplementary Methods) and lasted 10 min/day for 5-7 days. The recording headstage was wireless connected to the amplifier, which filtered and stored the data (Triangle BioSystems Int.). Data were analyzed off-line both by Matlab software and by locally developed Python routines. Electrophysiological signals were digitized and recorded with band-pass filters at 300–6000 Hz for spikes, 1–120 Hz for LFPs. The latter were further filtered with a Notch filter (3 Hz width) to remove the contribution of electrical line noise and downsampled to 0.5 KHz [49, 50]. The spikes were extracted and sorted by using the Wave_clus Matlab toolbox. Spike waveforms were processed by principal component analysis of their wavelet coefficients [51]. Neurons resulting from sorting with average spiking rates below 2 Hz and above 100 Hz were excluded from the analysis. Recorded neurons were uniformly distributed over the recording electrodes. Putative inhibitory and excitatory neurons were clustered by estimating the duration of the hyperpolarized and depolarized states of each cell as previously described [52]. Signal separation in different LFP frequency bands was performed by a 5^th^ order band-pass Butterworth filter [53]. Phase synchronies between two recorded LFP sequences (x and y) were measured by the Welch’s coherence function:

$$\gamma \left( {x,y} \right) = \frac{{\left| {PSD(x,y)} \right|^2}}{{(PSD(x,x) \cdot PSD(y,y))}}$$

where PSD (x, x) and PSD (y, y) are the power spectral density estimates of the signals x and y, and PSD (x, y) is the cross spectral estimate of x and y. When *γ* is equal to 1 (0), then x and y are perfectly synchronous (asynchronous). To investigate the extent of functional connections between the recorded regions (CA1; amygdala, Amy; entorhinal cortex, ERC; perirhinal cortex, PRC), we reduced the 30 × 30 LFP connectivity to 8 × 8 (4 areas from 2 hemispheres) by averaging the LFP connectivity strength from electrodes within each region. In order to reduce type II errors we performed a non-parametric permutation test. Thus, we generated 10000 surrogate signals for each recorded electrode by using the amplitude adjusted fast Fourier transform. Only connections with strength greater or equal to the original connectivity strength in more than 9950 surrogates (*p* value < 0.05) were selected, otherwise discarded.

### Statistical analysis

Statistical analysis was conducted using Prism 9.4 software (GraphPad). Unpaired two-tailed Student’s *t*-test was used to compare two sample groups, unless the values were not normally distributed, in which case the non-parametric Mann-Whitney test was used. To determine whether the mean calculated from sample data collected from a single group was different from zero, the one-sample *t*-test was used. To compare more than two sample groups, one-way or two-way ANOVA was used followed by false discovery rate (FDR) *post hoc* test (two-stage linear step-up procedure of Benjamini, Krieger and Yekutieli). Data are presented as mean ± standard error of the mean (SEM). Differences were considered significant for *p* < 0.05. Asterisks (or alternative symbols, i.e., #, §, as indicated) refer to the following significance levels: *(§) *p* < 0.05; **(##, §§) *p* < 0.01; ***(§§§) *p* < 0.001. Unless differently indicated, data from dissociated neurons were obtained from at least 3 independent cultures. For behavioral experiments, sample size was estimated by using G*Power software [54]. No samples were excluded from the analyses and no randomization methods were applied. Statistical details (number of mice and recording sessions for in vivo experiments; number of mice, brain slices, cell number for ex vivo experiments; number of cultures, recording sessions, cells, and synapses for in vitro experiments; exact mean values; SEM; statistical tests and *p* values) can be found in the Supplementary Tables 1–14 and in figure legends.

## Results

### *Pcdh19* cKO mice show PCDH19 mosaic expression

**Fig. 1:**
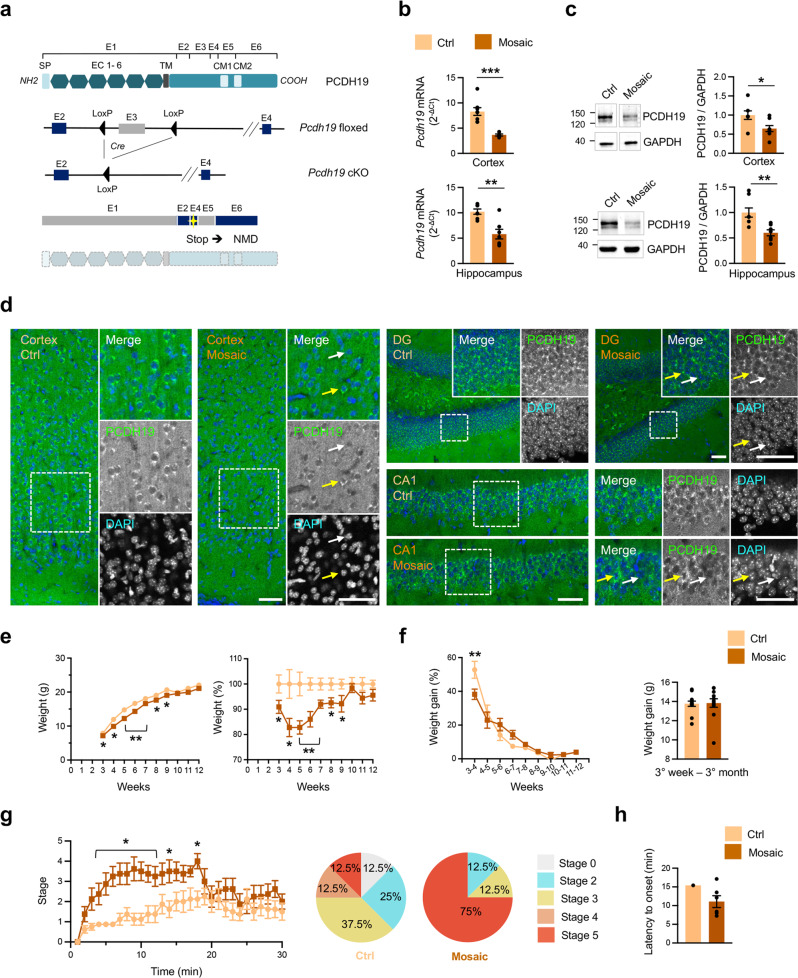
*Pcdh19* cKO female mice display mosaic expression of PCDH19 and increased seizure susceptibility. **a** Schematic representation of *Pcdh19* gene targeting strategy (E = exon; SP = signal peptide; EC = extracellular cadherin domain; TM = transmembrane domain; CM = conserved motif; Stop = premature stop codons, NMD = nonsense-mediated decay). **b** Quantification of *Pcdh19* expression in cerebral cortex (upper panel) and hippocampus (lower panel) from adult (P90) *Pcdh19* cKO female mice (*Pcdh19*^*fl/x*^ Syn1-Cre, Mosaic) compared to controls (*Pcdh19*^*fl/x*^, Ctrl). *Pcdh19* mRNA was normalized on *actin* mRNA level (Cortex: N, Ctrl 8, Mosaic 6; Hippocampus: N, Ctrl 7, Mosaic 7; Student’s *t*-test, **p < 0.01, ***p < 0.001). **c** Representative PCDH19 Western blots and relative quantification in cortex (upper panel) and hippocampus (lower panel) from adult (P90-120) mice as in **b**. PCDH19 expression was normalized on GAPDH (Cortex: N, Ctrl 7, Mosaic 8; Hippocampus: N, Ctrl 8, Mosaic 7; Student’s *t*-test, *p < 0.05, **p < 0.01). **d** IF on coronal brain slices from mice as in **b** at P30 stained for PCDH19 and DAPI. PCDH19 expression is shown in cortex (left panels) and hippocampus (DG, right top panels; CA1, right bottom panels). The inserts show higher magnification images; arrows indicate representative couples of PCDH19-positive (yellow arrows) and -negative (white arrows) neurons. Scale bars, 50 µm. **e** Growth curve of *Pcdh19* cKO mice (*Pcdh19*^*fl/x*^ Syn1-Cre, Mosaic) and controls (*Pcdh19*^*fl/x*^, Ctrl) showing mice weight expressed in grams (**g**, left) or in percentage (normalized on Ctrl, right) (N, Ctrl 22, Mosaic 20; mixed-effects two-way ANOVA and FDR *post hoc* test, *p < 0.05, **p < 0.01). **f** Weight gain in mice as in **e** calculated between consecutive weeks (gain expressed as percentage, left) (N, Ctrl 18, Mosaic 19; mixed-effects two-way ANOVA and FDR *post hoc* test, **p < 0.01) or between 3 weeks and 3 months of age (gain expressed in grams, right) (N, Ctrl 13, Mosaic 12). **g** Susceptibility of P26-28 *Pcdh19* cKO mice (*Pcdh19*^*fl/x*^ Syn1-Cre, Mosaic) and controls (*Pcdh19*^*fl/x*^, Ctrl) to pharmacologically-induced seizures. Left, scale of maximum seizure intensity as a function of time after PTZ injection (N, Ctrl 8, Mosaic 8; two-way ANOVA and FDR *post hoc* test, *p < 0.05). Right, pie charts showing the percentage of mice reaching a certain convulsive stage within 30 min after PTZ injection (N, Ctrl 8, Mosaic 8). **h** Latency to tonic-clonic seizures (stage 5) onset in the subgroup of mice as in **g** that experienced this seizure stage (N, Ctrl 1, Mosaic 6). All data (**b**–**c**, **e**–**h**) are shown as means ± SEM. See also Supplementary Table 1.

In order to mimic *PCDH19*-related syndrome, we generated a cKO mouse for *Pcdh19* (*Pcdh19* cKO) by exploiting Cre/loxP technology. *Pcdh19* exon 3 was flanked by two loxP sites (*Pcdh19* floxed) to cause its Cre-mediated excision. This was expected to cause reading frameshift generating premature stop codons that activate mRNA nonsense-mediated decay (NMD) (Fig. 1a). To verify this, we prepared primary cortical and hippocampal neurons from *Pcdh19* floxed mice (*Pcdh19*^*fl/fl*^ and *Pcdh19*^*fl/y*^) at P0 and infected them with AAVs encoding GFP-Cre (GFP-Cre AAVs) at DIV0. *Pcdh19* mRNA expression in infected and uninfected (control) neurons was analyzed at DIV7. No significant transcript level was detected by RT-PCR in GFP-Cre expressing neurons using probes targeting regions both downstream (exons 4–5 and 5–6) and upstream (exons 1–2) of exon 3, thus allowing to exclude the generation of mutant transcripts that could encode a truncated PCDH19 variant (Supplementary Fig. 1a). IF and Western blot experiments reconfirmed the loss of PCDH19 expression in GFP-Cre expressing neurons (Supplementary Fig. 1b, c).

Having verified the effect of exon 3 excision, we next induced *Pcdh19* deletion in vivo. PCDH19 is a synaptic protein expressed in both excitatory and inhibitory neurons (Supplementary Fig. 2a–c). To target both neuronal populations, we crossbred *Pcdh19* floxed female mice (*Pcdh19*^*fl/fl*^) with transgenic male mice expressing the Cre recombinase under the control of rat Synapsin I promoter (Syn1-Cre). As expected, the progeny that inherited the Syn1-Cre transgene (50%, *Pcdh19* cKO: females *Pcdh19*^*fl/x*^ Syn1-Cre and males *Pcdh19*^*fl/y*^ Syn1-Cre) displayed the excision of *Pcdh19* exon 3 selectively in the brain tissue (Supplementary Fig. 3a, b). *Pcdh19* cKO female mice (*Pcdh19*^*fl/x*^ Syn1-Cre), which have one WT and one floxed *Pcdh19* allele, were characterized by *Pcdh19* mosaic expression. In cortex and hippocampus of these mice, *Pcdh19* mRNA was reduced by approximately 55% and 43%, respectively (Fig. 1b) and PCDH19, evaluated by western blot, was reduced by nearly 40% in both areas (Fig. 1c). IF experiments showed the coexistence of PCDH19-positive and PCDH19-negative cells in cortex and hippocampus (Fig. 1d), brain areas characterized by high levels of PCDH19 expression. Heterozygous *Pcdh19* floxed females that did not inherit the Syn1-Cre transgene (*Pcdh19*^*fl/x*^), as well as homozygous *Pcdh19* floxed females (*Pcdh19*^*fl/fl*^), expressed normal levels of PCDH19, demonstrating that loxP sites do not perturb gene expression (Supplementary Fig. 3c).

*Pcdh19* mosaic expression is considered a key determinant of DEE9, making *Pcdh19* cKO female mice, hereinafter simply referred to as *Pcdh19* mosaic mice, a suitable model to study this syndrome.

### *Pcdh19* mosaic mice show increased seizure susceptibility and behavioral defects associated with altered synapse plasticity and structure

*Pcdh19* mosaic mice (*Pcdh19*^*fl/x*^ Syn1-Cre) were viable and did not show gross physical or neuroanatomical abnormalities, but were characterized by transient growth retardation. Adolescent *Pcdh19* mosaic mice weighted significantly less than control mice (*Pcdh19*^*fl/x*^) (3^th^-9^th^ postnatal week, Fig. 1e). However, this was generally compensated over time. The weight gain curve of *Pcdh19* mosaic mice surpassed that of controls starting from the 5^th^ postnatal week and in adulthood they reached a comparable body weight to controls (Fig. 1f).

We did not observe spontaneous seizures in *Pcdh19* mosaic mice; however, they displayed higher seizure susceptibility when treated with the GABA_A_R antagonist PTZ. 4 week-old mice (P26-28) were observed for 30 min after receiving a single dose of PTZ (40 mg/Kg). *Pcdh19* mosaic mice exhibited significantly more severe seizures than their Cre-negative littermates. The majority (75%) of *Pcdh19* mosaic mice displayed at least one generalized tonic-clonic seizure with loss of posture and lying on their side (stage 5), compared to only one control littermate (12.5%) (Fig. 1g). In *Pcdh19* mosaic mice, generalized tonic-clonic seizures were often preceded by vocalization (83,3% of mice) and their latency was on average shorter than that experienced by the control mouse (Fig. 1h).

**Fig. 2:**
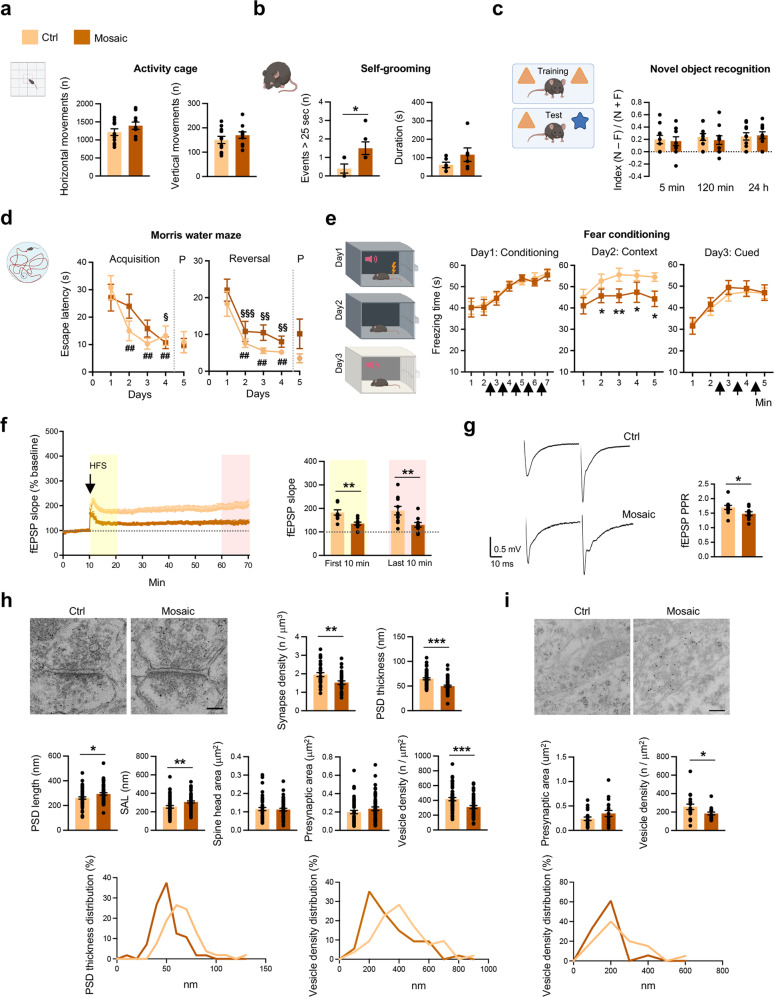
*Pcdh19* mosaic mice display behavioral deficits and synaptic defects. **a** Spontaneous motor activity of *Pcdh19* mosaic mice (*Pcdh19*^*fl/x*^ Syn1-Cre, Mosaic) and controls (*Pcdh19*^*fl/x*^, Ctrl) evaluated by activity cage test (N, Ctrl 11, Mosaic 10). **b** Self-grooming test in mice as in **a** (N, Ctrl 5, Mosaic 6; Student’s *t*-test, *p < 0.05). **c** Recognition memory of mice as in **a** assayed by novel object recognition test (N = novel object, F = familiar object; N, Ctrl 10, Mosaic 10). **d** Morris water maze test on mice as in **a** (acquisition phase, left; reversal phase, right; P = probe test) (N, Ctrl 11, Mosaic 10; acquisition and reversal phase: two-way ANOVA, n.s.; one-way ANOVA and FDR *post hoc* test, Mosaic, ^§^*p* < 0.05, ^§§^*p* < 0.01, ^§§§^*p* < 0.001, Ctrl, ^##^*p* < 0.01; probe tests: Student’s *t*-test, n.s.). **e** Fear conditioning test in mice as in **a** (from left to right: conditioning, context, cued phase) (N, Ctrl 9, Mosaic 9; two-way ANOVA and FDR *post hoc* test, *p < 0.05, **p < 0.01). Arrows = tones. **f** LTP recorded in hippocampal slices (CA1) from mice as in **a** in response to HFS (100 stimuli at 100 Hz) of Schaffer collaterals. fEPSPs slope (expressed as percentage of the baseline) relative to the entire recording period (left) and the first and last 10 min of recording (right) are shown (N, 10 slices/genotype from 4 Ctrl and 5 Mosaic mice; Student’s *t*-test, **p < 0.01). **g** Presynaptic short-term plasticity in mice as in **a** assessed by PPR with an interstimulus interval of 50 ms (the stimulus artifact has been removed for clarity; N, 10-11 slices, from 4 Ctrl and 5 Mosaic mice; Student’s *t*-test, *p < 0.05). **h** Representative electron micrographs of excitatory synapses on apical dendrites of CA1 pyramidal neurons from P21 mosaic mice (*Pcdh19*^*fl/x*^ Syn1-Cre, Mosaic) and controls (*Pcdh19*^*fl/x*^, Ctrl) (scale bar, 100 nm) and quantification of synapse density, structural features and analysis of values distribution (N mice, Ctrl 2, Mosaic 2; N synapses > 50/genotype; synapse density evaluated on 615.00 µm^2^/genotype; Mann-Whitney test or Student’s *t*-test as indicated in Supplementary Table 2, *p < 0.05, **p < 0.01, ***p < 0.001). **i** Representative electron micrographs of CA1 inhibitory synapses from mice as in **h** (scale bar, 200 nm), quantification of presynaptic features and analysis of values distribution (N mice, Ctrl 2, Mosaic 2; N synapses > 18/genotype; Student’s *t*-test, *p < 0.05). All data (**a**–**i**) are shown as means ± SEM. See also Supplementary Table 2. The figures to schematize behavioral tests were created with BioRender.com.

To investigate whether *Pcdh19* mosaic mice could recapitulate cognitive and autistic traits of DEE9, we tested them in behavioral paradigms. Spontaneous activity evaluated in an activity cage revealed no differences between *Pcdh19* mosaic mice and their Cre-negative littermate controls (Fig. 2a). When tested for self-grooming to assess repetitive and stereotyped behaviors associated with ASD, *Pcdh19* mosaic mice scored more long-lasting events (Fig. 2b). Despite performing normally in the novel object recognition test (Fig. 2c), *Pcdh19* mosaic mice displayed some difficulties in the Morris water maze and fear conditioning tests, suggesting hippocampal-dependent cognitive impairment.

Although *Pcdh19* mosaic mice and controls performed similarly in the Morris water maze test, mosaic mice showed a less steep learning curve compared to controls in the acquisition phase. Both groups learned the platform location by the last training session (day 4). However, while control mice took significantly less time to reach the hidden platform already by the second training session (day 2) compared to the first (day 1), *Pcdh19* mosaic mice significantly improved their performance only by day 4. No differences between the two groups were observed in the probe tests and reversal phase (Fig. 2d).

In the fear conditioning test, the freezing behavior of *Pcdh19* mosaic mice and controls were comparable during the conditioning phase (day 1), when mice were exposed to paired auditory cues (conditioned stimulus) and mild electric foot shocks (aversive unconditioned stimulus). However, when re-exposed to the same context after 24 hours (day 2), the freezing time of *Pcdh19* mosaic mice was reduced compared to controls. A similar freezing behavior between groups was observed on day 3, when animals were exposed to the auditory tone in a different context (Fig. 2e), thus suggesting that *Pcdh19* mosaic mice learned and remembered the association with the auditory cue but not with the spatial context.

According to the close relationship existing between cognitive tasks, in particular contextual learning, and hippocampal synaptic plasticity [55, 56], we hypothesized that the behavioral defects observed could be associated with impairment in synaptic function, also in view of the synaptic expression of PCDH19 (Supplementary Fig. 2b, c).

To validate our hypothesis, we prepared acute hippocampal slices from adult *Pcdh19* mosaic mice and control littermates and measured LTP and PPR in CA1 neurons. LTP at Shaffer collaterals-CA1 synapses was strongly reduced in *Pcdh19* mosaic mice (Fig. 2f). The significantly reduced PPR in *Pcdh19* mosaic mice compared to controls indicated that also short-term plasticity was impaired (Fig. 2g).

According to ultrastructure analysis, the *stratum radiatum* of CA1 area from *Pcdh19* mosaic mice was characterized by reduced number of excitatory synapses with altered postsynaptic density (PSD) morphology. In particular, PSD thickness was reduced, PSD length and synaptic appositions length (SAL) were slightly increased, while spine head area was unchanged in mosaic mice compared to controls (Fig. 2h). The density of neurotransmitter-containing vesicles, but not the presynaptic area, was significantly reduced in both excitatory and inhibitory synapses of mosaic mice (Fig. 2h, i). Altered synaptic parameters, i.e. PSD thickness and vesicle density, showed a unimodal distribution in both mouse groups, suggesting a uniform effect of mosaic *Pcdh19* deletion on synapse ultrastructure (Fig. 2h, i).

Similar results were obtained when *Pcdh19* mosaic expression was achieved through the ICV injection of AAVs expressing Cre in *Pcdh19* floxed (*Pcdh19*^*fl/fl*^ and *Pcdh19*^*fl/y*^) neonatal mice. This model, characterized by a comparable reduction (−45%) of PCDH19 protein expression in the hippocampus (Supplementary Fig. 4a), reproduced the reduction in excitatory synapse density, vesicle density and PSD thickness. While PSD length was unaffected, a slight decrease of spine head area was observed. Unimodal distributions of PSD thickness and vesicle density were reconfirmed (Supplementary Fig. 4b–d).

Altogether, these data indicate that *Pcdh19* mosaic mice recapitulate key traits of DEE9 syndrome. Mosaic mice are characterized by higher susceptibility to pharmacologically induced seizures and behavioral deficits, which are associated with functional and structural synaptic defects.

### Neurons that do not express *Pcdh19* within the mosaic brain show hyperexcitability

**Fig. 3:**
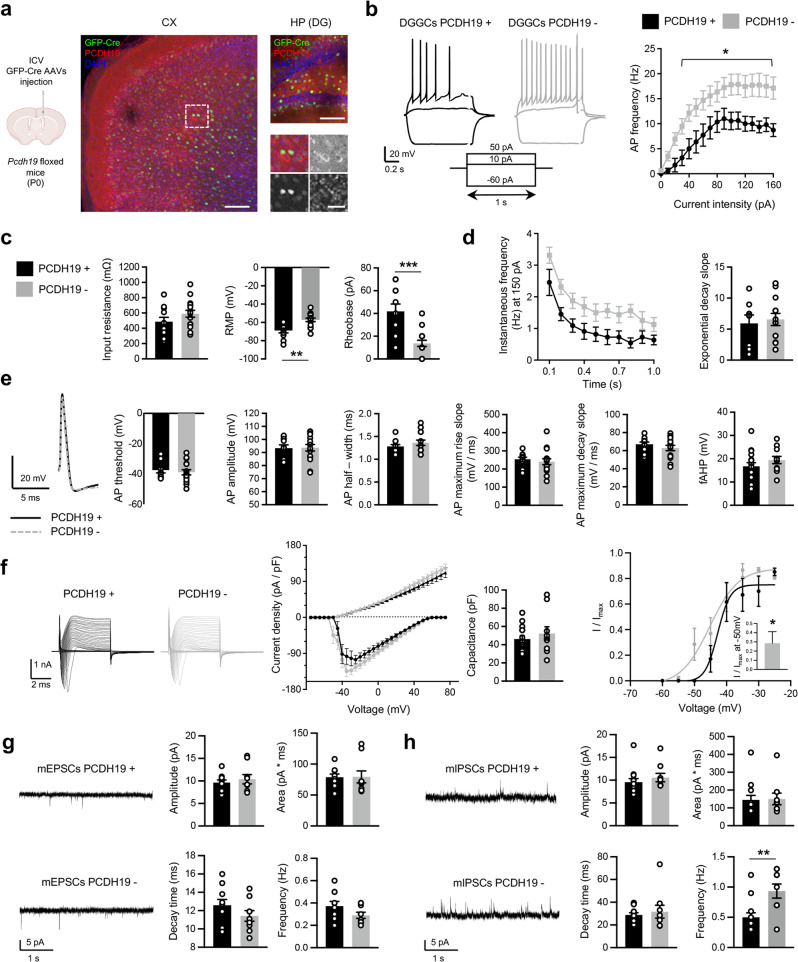
Neurons in the *Pcdh19* mosaic brain display heterogeneous excitability. **a** Cartoon showing ICV injection of AAVs in *Pcdh19* floxed mice (*Pcdh19*^*fl/fl*^) and representative IF images of cortex (CX) and hippocampus (HP, DG region). Higher magnification images in the inserts show a couple of cortical neurons infected with GFP-Cre not expressing PCDH19 next to a couple of uninfected neurons expressing PCDH19 (in red). Scale bar, 100 µm (CX, lower magnification and HP), 40 µm (CX, higher magnification). The cartoon was created with BioRender.com. **b** Representative traces of APs evoked by −60, +10 and +50 pA of injected current in GFP-Cre expressing DGGCs (PCDH19-) and controls (PCDH19+) (left) and AP frequency at different current step intensities (right) from *Pcdh19* floxed mice (*Pcdh19*^*fl/fl*^) injected with AAVs as shown in **a** (N DGGCs from 3 mice, PCDH19+11, PCDH19- 16; two-way ANOVA and FDR *post hoc* test, *p < 0.05). **c** AP input resistance, RMP and rheobase of neurons as in **b** (N DGGCs from 3 mice, PCDH19+ 11, PCDH19- 16; Student’s *t*-test, **p < 0.01, ***p < 0.001). **d** Instantaneous frequency and exponential decay slope of APs induced by 150 pA of injected current in DGGCs from mice as in **b** (N DGGCs from 3 mice, PCDH19+ 11, PCDH19- 16). **e** Representative traces of APs evoked at rheobase in PCDH19+ and PCDH19- DGGCs from mice as in **b** and quantification of AP threshold, amplitude, half-width, kinetic and fAHP (N DGGCs from 3 mice, PCDH19+ 11, PCDH19- 16). **f** Voltage-clamp experiments in PCDH19+ and PCDH19- DGGCs from mice as in **b**. From left to right: representative traces and current density of inward and outward currents, neuronal capacitance, activation kinetic of inward currents mediated by voltage-gated ion channels and histogram showing I/Imax at −50mV (N DGGCs from 4 mice, PCDH19+ 10, PCDH19- 15; one-sample *t*-test, *p < 0.05). **g** Representative traces and quantification of mEPSCs recorded in PCDH19+ and PCDH19- DGGCs from mice as in **b** (N DGGCs from 4 mice, PCDH19+ 10, PCDH19- 9). **h** Representative traces and quantification of mIPSCs recorded in PCDH19+ and PCDH19- DGGCs from mice as in **b** (N DGGCs from 4 mice, PCDH19+ 13, PCDH19- 9; Student’s *t*-test, *p < 0.05). All data (**b**–**h**) are shown as means ± SEM. See also Supplementary Table 3.

We recently reported that PCDH19 downregulation associates with increased excitability in primary hippocampal neurons [57]. This prompted us to investigate whether PCDH19-positive (PCDH19+) and PCDH19-negative (PCDH19-) cells within the *Pcdh19* mosaic brain are characterized by different intrinsic excitability. For these experiments, *Pcdh19* mosaic expression was obtained through ICV injection of GFP-Cre AAVs in *Pcdh19* floxed mice (*Pcdh19*^*fl/fl*^), in order to distinguish cells not expressing (PCDH19-, GFP-positive) or expressing PCDH19 (PCDH19+, GFP-negative) based on GFP fluorescence (Fig. 3a).

We prepared acute hippocampal slices from adult *Pcdh19*^*fl/fl*^ mice injected at P0 and measured AP firing and membrane properties in neurons of the DG, the “gate” which protects hippocampal circuits from overexcitation [58]. PCDH19- DG granule cells (DGGCs) were characterized by higher firing frequency in response to depolarizing injected currents compared to PCDH19+ neurons (Fig. 3b). While input resistance was comparable between groups, PCDH19- DGGCs showed depolarized resting membrane potential (RMP) and reduced rheobase compared to neighboring PCDH19+ DGGCs (Fig. 3c). No differences were observed in spike accommodation (Fig. 3d), AP voltage threshold, amplitude, kinetics, and fast afterhyperpolarization (fAHP) between the two neuronal subpopulations (Fig. 3e).

Among possible contributors to increased neuronal excitability, we investigated voltage-gated ion channels functioning by measuring ion currents at different membrane voltage values. While outward currents were comparable between groups, inward currents were detected at more negative potentials (starting from −50 mV) in PCDH19- neurons compared to PCDH19+ neurons. This resulted in a leftward shift in the activation curve of channels mediating inward currents in PCDH19- neurons, possibly contributing to their hyperexcitability (Fig. 3f). Excitability levels can shape the way neurons form synaptic connections [59]. To gain insights into the synaptic inputs of PCDH19+ and PCDH19- neurons, we measured mIPSCs and mEPSCs. While amplitude, area and decay time of spontaneous currents were similar in the two groups, PCDH19- neurons were characterized by a significant higher frequency of mIPSCs and a decreasing trend in the frequency of mEPSC, suggesting an unbalance in favor of inhibitory synaptic inputs (Fig. 3g, h).

Taken together, these results indicate that PCDH19- neurons in the mosaic hippocampus display hyperexcitability associated with altered voltage-gated channels currents and a concomitant increase of mIPSCs frequency with respect to PCDH19+ neurons.

### *Pcdh19* mosaic networks show reduced firing and increased synchronization

Neuronal excitability levels can profoundly affect whole network functioning [60]. This notion, and data indicating structural and functional alterations in synapses, prompted us to analyze network activity. To this end, we performed MEA recordings, first in vitro in cultured neurons, and next ex vivo in acute hippocampal slices from *Pcdh19* mosaic mice (*Pcdh19*^*fl/x*^ Syn1-Cre) and control littermates (*Pcdh19*^*fl/x*^) obtained by crossbreeding of *Pcdh19* floxed mice with Syn1-Cre mice.

**Fig. 4:**
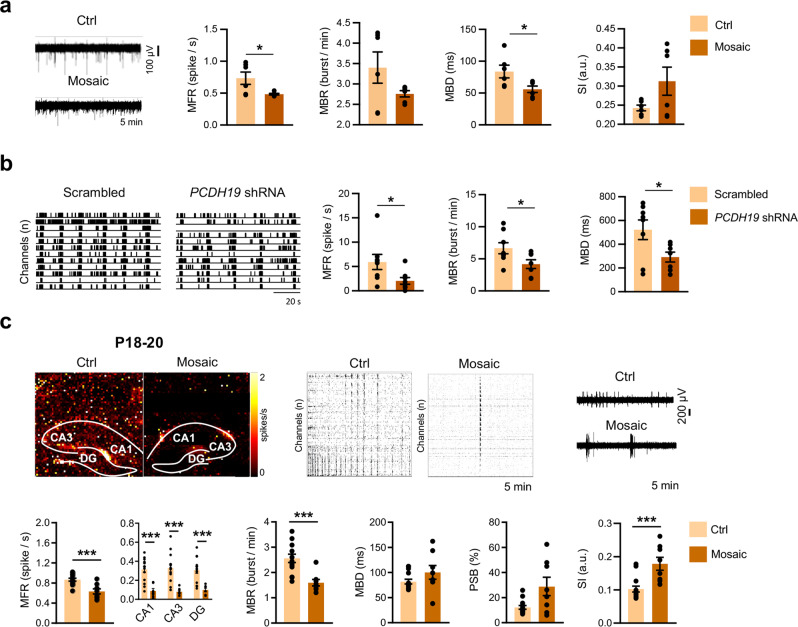
*Pcdh19* mosaic networks show reduced activity and increased synchronization. **a** Representative traces of MEA activity recorded from *Pcdh19* floxed (*Pcdh19*^*fl/fl*^ and *Pcdh19*^*fl/y*^) primary cortical neurons, either uninfected (Ctrl) or infected with GFP-Cre AAVs to reproduce PCDH19 mosaicism, and quantification of MFR, MBR, MBD, and SI in both neuronal cultures (N cultures, Ctrl 1, Mosaic 1, N recordings, 3 at DIV 16 + 3 at DIV21 for each culture; Student’s *t*-test, *p < 0.05). **b** MEA recordings from hiPSCs-derived cortical neurons expressing *PCDH19* shRNA or a scrambled shRNA. Representative raster plots of electrophysiological activity and relative quantification (N cultures, Ctrl 8, Mosaic 7-8; Student’s *t*-test, *p < 0.05). **c** Top left panel, representative images of MEA activity maps in pseudocolors, showing firing activity of hippocampal slices from *Pcdh19* mosaic (*Pcdh19*^*fl/x*^ Syn1-Cre, Mosaic) and control mice (*Pcdh19*^*fl/x*^, Ctrl) at P18-20. Top middle panel, representative raster plots showing electrical activity recorded from 100 active channels over 5 min. Top right panel, representative traces recorded from a single electrode over 5 min in Ctrl and Mosaic mice. Bottom panels, quantification of MFR, MBR, MBD, percentage of spikes in busts (PSB), and SI in Ctrl and Mosaic mice (N slices, Ctrl 14, Mosaic 8, from 6 Ctrl and 4 Mosaic mice; Mann-Whitney test or Student’s *t*-test, ***p < 0.001). All data (**a**–**c**) are shown as means ± SEM. See also Supplementary Table 4.

Cultured cortical neurons obtained from *Pcdh19* floxed mice (*Pcdh19*^*fl/fl*^ and *Pcdh19*^*fl/y*^) at P0 were sparsely transduced with GFP-Cre AAVs at DIV0 in order to reproduce PCDH19 mosaic expression, and were recorded when mature (DIV16-21). Compared to untransduced neurons (control), mosaic cultures were characterized by a significantly reduced global firing frequency and by reduced burst duration. Furthermore, a tendency in increased synchronization index (SI) was observed (Fig. 4a).

To verify whether a network composed by neurons of human origin could recapitulate this phenotype, we prepared cortical neurons from hiPSCs. We downregulated *PCDH19* expression with a specific shRNA at DIV7 and performed MEA recordings at DIV35. Notably, we observed that spike rate and burst rate and duration were significantly reduced compared to control neurons expressing a scrambled shRNA (Fig. 4b).

Reduced network activity was reconfirmed ex vivo in hippocampal slices, which were recorded in Mg^2+^-free aCSF solution supplemented with the potassium channel blocker 4-aminopyridine to increase neuronal activity. In preadolescent (P18-20) *Pcdh19* mosaic mice, approaching the critical time-window of growth, we found a significant reduction in hippocampal network mean firing rate (MFR) and mean bursting rate (MBR) compared to controls. Mean duration of bursts (MBD) and the percentage of spikes in bursts (PSB) were comparable between groups. The degree of synchronization of the hippocampal network, evaluated with the cross-correlation method [61], appeared higher in *Pcdh19* mosaic mice compared to controls (Fig. 4c). To investigate whether this phenotype might be developmentally controlled, we extended the analysis to early-postnatal (P8-10) and adult (P60-90) mice. We found that a reduction in MFR and MBR, together with an increase in the SI, was detectable already in the early-postnatal period, corresponding to PCDH19 expression peak [21], and persisted in adulthood (Supplementary Fig. 5a, b).

We concluded that PCDH19 mosaic expression associates with reduced network firing in both rodent and human neurons. In mouse hippocampal slices, reduced network activity associates with increased synchronization and persists from the early-postnatal period to adulthood.

### The limbic system of *Pcdh19* mosaic mice shows globally reduced firing and excitatory/inhibitory unbalance

To validate the data obtained in vitro and in hippocampal slices and extend the analysis to additional regions of the limbic system, we recorded neuronal network activity in vivo.

**Fig. 5:**
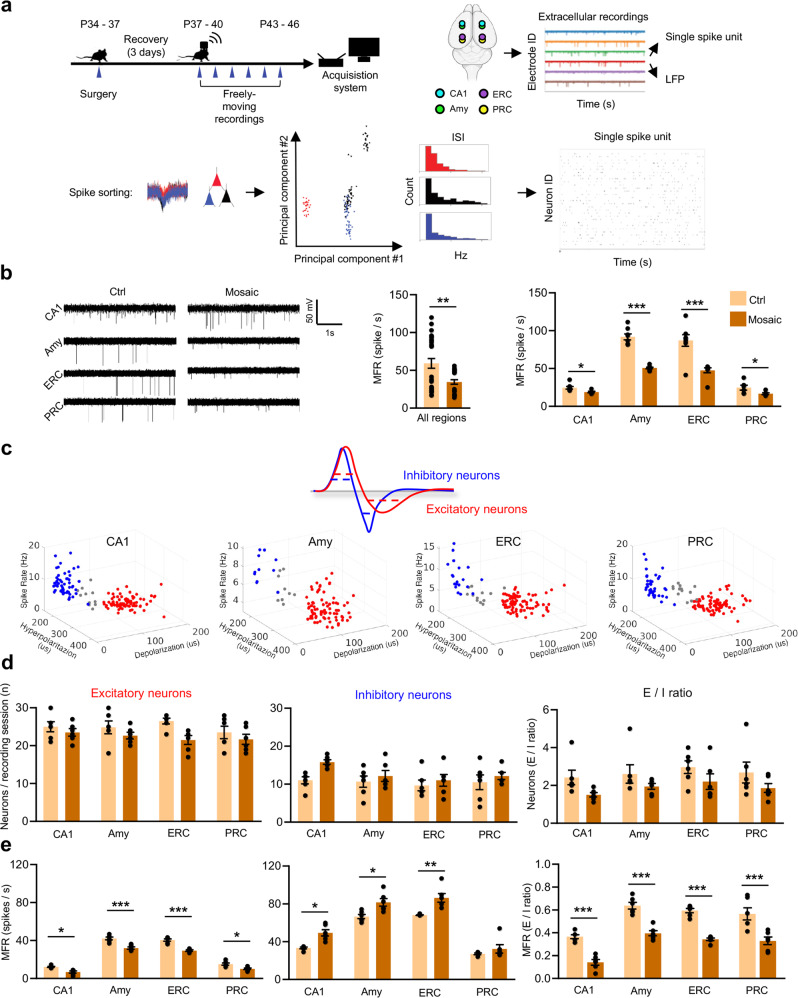
Network activity and E/I ratio are reduced in the limbic system of *Pcdh19* mosaic mice. **a** Top left, in vivo recordings experimental planning. Top right, electrodes position in limbic system regions and representative extracellular recordings from which single spike units and LFP signals were extrapolated. Bottom, spike sorting process used to extrapolate single spike unit activity (ISI = interspike interval). **b** Representative traces of neuronal extracellular activity (left panel) and MFR (right panels) in different limbic system regions of mosaic mice (*Pcdh19*^*fl/y*^ Syn1-Cre, Mosaic) and controls (*Pcdh19*^*fl/y*^, Ctrl) (N, 5-7 recording sessions/brain region/mouse, 2 mice/genotype; Student’s *t*-test, *p < 0.05, **p < 0.01, ***p < 0.001). **c** Classification of neurons into excitatory and inhibitory according to the duration of AP depolarization and hyperpolarization phases. Hierarchical clustering allowed sorting of neurons from *Pcdh19* mosaic and control mice as in **b** (red, excitatory neurons; blue, inhibitory neurons; grey, unclassified neurons). **d** Number of excitatory and inhibitory neurons and ratio in limbic system regions of Mosaic and Ctrl mice as in **b** (N, 5-7 recording sessions/brain region/mouse, 2 mice/genotype). **e** MFR of excitatory and inhibitory neurons and MFR ratio in limbic system regions of Mosaic and Ctrl mice as in **b** (N, 5-7 recording sessions/brain region/mouse, 2 mice/genotype; Student’s *t*-test, *p < 0.05, **p < 0.01, ***p < 0.001). All data (**b**, **d**, **e**) are shown as means ± SEM. See also Supplementary Table 5.

To this end, microelectrodes were implanted in the brain of adolescent (P34-37) mice in correspondence of anatomo-functional connected regions of the limbic system: hippocampal CA1, amygdala (Amy), entorhinal cortex (ERC) and perirhinal cortex (PRC). Each region in each hemisphere was sampled by 3/4 microelectrodes, for a total of 30 microelectrodes (15/hemisphere) plus one reference microelectrode for each hemisphere (Supplementary Fig. 6). We chose to perform these experiments on male mice as they recover better from surgery than females, especially when individually housed [62]. This was made possible by the observation that *Pcdh19* cKO male mice (*Pcdh19*^*fl/y*^ Syn1-Cre) showed PCDH19 mosaicism to a comparable extent to females (Supplementary Fig. 7a–c). This is consistent with Cre recombinase efficiency lower than 100%, most likely due to the modest transgene expression level driven by the Synapsin I promoter. Cre-negative male littermates (*Pcdh19*^*fl/y*^), which express normal level of PCDH19 (Supplementary Fig. 7d), were used as controls. After recovery from the surgery, the network activity was recorded in freely behaving mice. Extracellular signals were sorted according to their neurophysiological origin into spiking activity and LFPs and analyzed separately (Fig. 5a). The MFR, obtained by averaging recordings from all channels, was strongly reduced in *Pcdh19* mosaic mice compared to controls. In particular, the reduction was of 16% in the CA1, 47% in Amy, 44% in ERC and 31% in PRC (Fig. 5b). These results confirmed those obtained in vitro in mouse and human neurons, and ex vivo in murine hippocampal slices, and clearly indicated that *Pcdh19* mosaic expression associates with decreased neuronal network firing rate.

Next, we classified the neurons of each brain region into putative excitatory or inhibitory neurons according to the half width of their hyperpolarization and depolarization states [52] and we measured their number and spiking rate across recording sessions, in order to estimate their relative contribution to the network activity. A comparable number of excitatory and inhibitory neurons were found in *Pcdh19* mosaic mice and controls. However, in *Pcdh19* mosaic mice the excitatory neurons showed a decreased spiking rate compared to controls across all limbic system areas. Conversely, the spiking rate of inhibitory neurons was significantly increased in *Pcdh19* mosaic mice compared to controls across all limbic system areas analyzed, with the exception of PRC. As a result, in *Pcdh19* mosaic mice the excitatory/inhibitory (E/I) ratio inferred from the spiking activity was shifted in favor of the latter (Fig. 5c–e).

### The limbic system of *Pcdh19* mosaic mice shows altered network connectivity

**Fig. 6:**
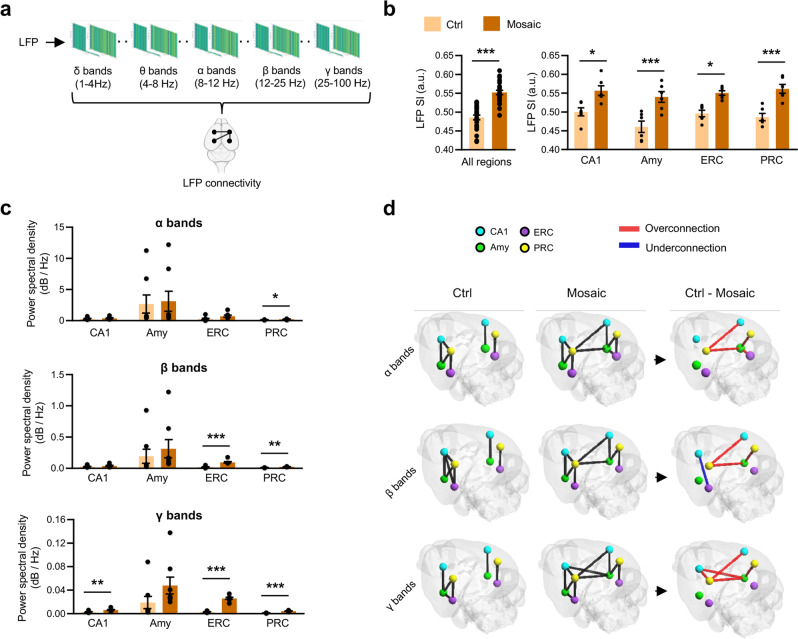
*Pcdh19* mosaic mice show hyperconnectivity among limbic system areas. **a** Experimental workflow scheme: extracellular neuronal activity was analyzed to obtain LFP signals at discrete frequency bands (1–100 Hz interval) and extrapolate functional connectivity between limbic system areas. **b** LFP SI relative to recordings in all limbic system regions (left) and in single regions (right) from mosaic mice (*Pcdh19*^*fl/y*^ Syn1-Cre, Mosaic) and controls (*Pcdh19*^*fl/y*^, Ctrl) (N, 5-7 recording sessions/brain region/mouse, 2 mice/genotype; Student’s *t*-test, *p < 0.05, ***p < 0.001). **c** LFP power spectral density in α, β, and γ bands from limbic system regions of mice as in **b** (N, 5-7 recording sessions/brain region/mouse, 2 mice/genotype; Student’s *t*-test, *p < 0.05, **p < 0.01, ***p < 0.001). **d** Connectome maps illustrating functional connections between limbic system regions of the ipsi- and contralateral hemisphere, extrapolated from LFP signals in α, β and γ bands in mice as in **b**. Differential connections emerging from the comparison between the two groups are shown in red (overconnections in Mosaic mice) and blue (underconnections in Mosaic mice). All data (**b**, **c**) are shown as means ± SEM. See also Supplementary Table 6.

After spiking activity, we extended the analysis to the slow components of network activity, the LFPs (Fig. 6a). First, we calculated LFPs SI in the different limbic system regions and we observed a global increase of this parameter in *Pcdh19* mosaic mice compared to controls (Fig. 6b). Next, we measured power spectral density. We observed an increase in all high-frequency bands (α, 8–12 Hz; β, 12–25 Hz; γ, 25–100 Hz) in the PRC, in β and γ bands in the ERC, and in γ bands in hippocampal CA1, while no frequency bands significantly varied in the amygdala of *Pcdh19* mosaic mice compared to controls (Fig. 6c). No difference was observed for lower frequencies (δ, 1–4 Hz; θ, 4–8 Hz) in any limbic system region analyzed (Supplementary Fig. 8). These data were consistent with those obtained by MEA recordings in hippocampal slices from *Pcdh19* mosaic mice, in which we observed a general increase of LFPs power spectral density at higher frequencies. This phenotype was more evident in the early postnatal period and attenuated in preadolescence, ages at which also other LFP measurements were affected (LFP amplitude and duration). In adult mosaic mice the LFPs spectral content was more similar to that of controls, with the exception of the β frequency band, which was decreased (Supplementary Fig. 9a–g).

Finally, we exploited the LPF signals from in vivo recordings to extrapolate the functional connectivity between limbic system areas in each frequency band. High-frequency bands (from α to γ) revealed significant connectivity differences between *Pcdh19* mosaic mice and controls. In general, limbic system regions displayed more functional connections in *Pcdh19* mosaic mice compared to controls. Only one connection in β band between hippocampal CA1 and ERC was observed in controls but not in *Pcdh19* mosaic mice. Notably, inter-hemisphere connections appeared exclusively in *Pcdh19* mosaic mice (Fig. 6d).

Altogether, these data indicate that *Pcdh19* mosaic mice are characterized by an aberrant configuration of their network states, a phenomenon typically observed in epilepsy and its comorbidities [63].

## Discussion

Epilepsy and associated cognitive and behavioral deficits are considered brain-network disorders [63–65]. However, the pathophysiology of PCDH19-related syndrome at network level remains uncharacterized. In this study, we exploit the *Pcdh19* cKO mouse model to fill this gap. This model, obtained by delivering Cre recombinase in *Pcdh19* floxed mice via crossbreeding or ICV injection of AAVs, is characterized by mosaic expression of PCDH19 in neurons, a condition believed to trigger DEE9 [8]. We focused on hippocampus and, more generally, on the limbic system structures for their relevance in DEE9. In fact, these structures express high levels of PCDH19 [66, 67], are implicated in memory and emotional behavior and are affected by PCDH19-related seizures [68].

*Pcdh19* mosaic mice showed delayed growth in the transition between childhood and adulthood, when circuits undergo intense remodeling, and a similar phenotype was observed in Dravet syndrome mouse models in concomitance with seizure onset [69–72]. Even though *Pcdh19* mosaic mice did not show spontaneous seizures, in agreement with the phenotype of the constitutive *Pcdh19* KO model [17, 73], they showed higher susceptibility to pharmacologically-induced seizures. In mosaic mice, generalized tonic-clonic seizures induced by PTZ were often preceded by vocalization. Interestingly, affective symptoms associated with seizures, such as terrified expression and screams of fear, indicating involvement of the limbic system, are commonly observed in DEE9 patients [68]. *Pcdh19* mosaic mice reproduced subtle autistic traits and cognitive impairment, which characterize a large proportion of DEE9 patients. Albeit not so severe to compromise mice performances across all tests performed, cognitive deficits were associated with marked structural and functional defects observed in hippocampal synapses. The density of excitatory synapses was reduced and their fine structure affected. In parallel, long-term and short-term plasticity were impaired, as inferred from reduced LTP and PPR. Even though the observed reduced PPR is generally ascribed to increase release probability, we cannot exclude that it might rather reflect the depletion of synaptic vesicles following the first stimulus [74, 75], in agreement with the reduced content of synaptic vesicles that we observed in both excitatory and inhibitory synapses.

Some synaptic defects could be a direct consequence of PCDH19 loss, given the expression of PCDH19 at synapses and its interplay with GABA_A_R [21] and NCAD [31], molecules involved in the maturation and stabilization of inhibitory and excitatory synapses, respectively [76, 77]. In particular, PCDH19 mismatch at synapses has recently been shown to affect NCAD-dependent signaling at presynaptic terminals of mossy fibers, impairing their function [31].

However, our distribution analyses are consistent with a homogeneous alteration of the ultrastructure of synapses, despite the different patterns of PCDH19 expression that are expected in the synapses of a mosaic brain (e.g. mismatched vs. matched expression at pre- and post-synaptic sites). Thus, reduction of excitatory synapses density and structural remodeling suggesting their weakening (such as reduced vesicle density and PSD thickness) may alternatively represent an attempt to compensate for the hyperexcitability of PCDH19-negative neurons within the mosaic brain. Indeed, lowering excitatory innervation is one of the neural strategies applied to balance high intrinsic excitability [78]. Our data anticipate that increased neuronal excitability might result from altered functioning of ion channels, which deserves closer examination in future studies.

Increasing the firing of inhibitory neurons while decreasing that of excitatory ones, as inferred from spike sorting in vivo, appears as an additional activity-reducing strategy. Furthermore, ex vivo analysis of spontaneous synaptic currents frequency suggests that, within the *Pcdh19* mosaic brain, synapses are redistributed to increase inhibitory inputs to hyperexcitable PCDH19-negative neurons, compared to neighboring PCDH19-expressing neurons. Thus, despite the reduced density of neurotransmitter vesicles observed in the inhibitory synapses of *Pcdh19* mosaic mice, and the reported role of PCDH19 in regulating GABA_A_R-mediated transmission [21, 32, 57], our data suggest an attempt of the mosaic brain to compensate for neuronal hyperexcitability by shifting the E/I balance in favor of the latter.

As a possible consequence of primary synaptic defects and/or overcompensating mechanisms, reduced network activity was observed in different experimental conditions sharing mosaic expression of PCDH19 (in vitro in mouse cortical neurons and hiPSCs-derived cortical neurons, ex vivo in mouse hippocampal slices, and in vivo in different limbic system regions of freely behaving mice) and across developmental stages (from the first postnatal period until adulthood, according to ex vivo recordings) and sexes.

The limbic system of *Pcdh19* mosaic mice was also characterized by a significant increase of synchronization in spiking activity and LFP oscillations. Hypersynchronization and the concomitant increase of LFP power spectral density could be due to the increased activity of inhibitory neurons, given their role in generating high-frequency oscillations in both local circuits and across brain regions [79, 80], and may contribute to undermining circuit stability by promoting epileptiform activity [79, 81] and compromise cognitive processes. In fact, high-frequency bands (α, β and γ) are involved in a variety of cognitive aspects such as memory, attention, and sensory-motor functions [82–85] and abnormal synchronization of oscillatory neuronal responses has been observed in several brain disorders, including epilepsy, ASD and schizophrenia [86–88].

The temporal covariance between activities in distributed neuronal populations allows the analysis of functional connectivity, which is increasingly recognized as a critical step for understanding brain function and dysfunction [89]. Notably, in *Pcdh19* mosaic mice we observed aberrant functional connections between limbic regions. In particular, we observed a diffused hyperconnectivity, a mechanism implemented by the brain to face with different insults [90] and a phenotype observed in animal models of ASD [91, 92] and in the brain of children with ASD across long- and short-range connections [86].

In conclusion, our study led to the identification of key functional features of neuronal networks associated with PCDH19 altered expression. We show that PCDH19-negative neurons within the *Pcdh19* mosaic brain are hyperexcitable and that the resulting network is characterized by a global reduction of neuronal activity, with an E/I balance shifted in favour of the latter. Increased neuronal synchronization and pattern of functional hyperconnectivity within limbic system regions were also observed. Altogether, these results point to PCDH19-related syndrome as a disorder of neuronal network, whose pathophysiology likely emerges from altered patterns of activity and connectivity between brain regions.

## Supplementary information

Supplementary Information

Supplementary Figure 1

Supplementary Figure 2

Supplementary Figure 3

Supplementary Figure 4

Supplementary Figure 5

Supplementary Figure 6

Supplementary Figure 7

Supplementary Figure 8

Supplementary Figure 9

## Data Availability

LFP phase synchrony was implemented in a python (≥ 3.6) language routine available at https://github.com/antoniozip/pcdh19_analysis.
