# Supplementary material for: Neuronal network activity and connectivity are impaired in a conditional knockout mouse model with PCDH19 mosaic expression
Source: Mol Psychiatry. 2023 Mar 30;29(6):1710–25. doi: 10.1038/s41380-023-02022-1 (PMC11371655; doi:10.1038/s41380-023-02022-1)
Supplement: Supplementary file 1 — Supplementary Information [file 41380_2023_2022_MOESM1_ESM.pdf]

SUPPLEMENTARY METHODS

Pcdh19 floxed mice

Pcdh19 floxed mice were generated at InGenious Targeting Laboratories, Inc. (USA) by inserting two loxP sites located 174 bp upstream and 225 bp downstream of exon 3, respectively. To this end, a targeting vector was constructed by using a fosmid clone. The targeting vector (15.4 kb) was composed by: 5' homology arm (6.8 kb), loxP site, the target region containing exon 3 (target region, 727 bp), loxP/FRT flanked Neomycin (Neo) selection cassette (2808 bp), 3' homology arm (5.1 kb). The targeting construct was subcloned from the fosmid and purified prior to electroporation into iTL BF1 (C57BL/6 FLP) embryonic stem (ES) cells, which express FLP recombinase vector to remove the selection cassette. Correctly targeted ES cells were microinjected into Balb/c blastocysts. Resulting chimeras were mated to C57BL/6 WT mice to generate germline Neo deleted mice.

ICV injection of AAVs

Pcdh19 floxed pups (P0) were anesthetized via cryo-anaesthesia and their head was disinfected with 70% ethanol. Sterilized glass micropipettes were prepared with a puller (Sutter Instrument, USA) and attached to a 3ml syringe through a silicon tube. Micropipettes loaded with the AAVs were inserted 2-3 mm deep into the skull of the anesthetized pups in order to reach the ventricles (0.25 mm lateral to the sagittal suture and 0.50-0.75 mm rostral to the neonatal coronary suture). 1 µl of AAV solution containing 5.5 x 10^9 GFP-Cre AAV9 particles (AAV9.hSyn.HI.eGFP-Cre.WPRE.SV40, Penn Vector, USA) (Fig. 3; Supplementary Fig. 1) or 3.4 x 10^9 GFP-2A-Cre AAV9 particles (AAV9-hSYN1-eGFP-2A-iCre-WPRE, Vector Biolabs) (Supplementary Fig. 4), 0.05% w/v trypan blue, and sterile PBS (mM: 137 NaCl, 2.7 KCl, 8 Na_2HPO_4 x $2H_2O$, 2.3 NaH_2PO_4, pH 7.4) was slowly injected in each ventricle. The micropipette stayed in position for 15 sec to prevent backflow. Pups were placed under a warming light until full recovery and finally returned to their home cage with the mother.

Behavioral tests

Adult (> P90) female mice, *Pcdh19* cKO (*Pcdh19$^{fl/x}$* Syn1-Cre) and control littermates (*Pcdh19$^{fl/x}$*), were used for behavioral experiments.

Spontaneous motor activity: Mice locomotion was evaluated by recording animals in an automated activity cage (43 x 43 x 32 cm) (Ugo Basile). Beam breaks were counted for 30 min to extrapolate horizontal and vertical movements.

Self-grooming: Mice were habituated to a transparent cylinder (46 x 23.5 x 20 cm) for 10 min and self-grooming events in the following 10 min were recorded and analyzed manually.

Novel object recognition: After 10 min of habituation in the test cage, two different objects were placed at the center of the cage and mice were free to explore them for 20 min (familiarization phase) before returning to their home cage. After 5 min, 120 min, and 24 hours mice returned to the test cage, in which one of the two familiar objects had been replaced by a novel one, for 20 min (recognition phase). The discrimination index (I) was calculated as the difference between the exploration time of the novel object (N) and the exploration time of the familiar one (F) on the total time of exploration: I = (N-F) / (N+F). Exploration was achieved when mice stayed within 0.5 cm from the object with the nose toward the object.

Morris water maze: The Morris water maze test was performed in a circular pool (154 cm in diameter x 38 cm in height). A circular hidden platform with a diameter of 13.5 cm was inside the pool, 0.5 cm below the surface of the water. The swimming pool was filled with water at a constant temperature (22°C ± 2°C) and made opaque with the addition of non-toxic white tempera paint to hide the platform. First, mice were habituated to the water maze (60 sec) and to the platform (60 sec). Next, in the acquisition phase, mice performed 4 trials/day (with 1 hour inter-trial interval) for 4 consecutive days: each mouse was placed in the pool with the platform in a fixed position, but changing the mouse starting position on each trial. Mice unable to find the platform within 60 sec (cut-off) were guided to the platform and remained on it for 60 sec. The 5th day the probe test (duration, 60 sec) was performed, in which the platform was removed. Finally, in the reversal phase, starting 2 days after

the probe test, mice performed 4 trials/day (trial cut-off, 60 sec; 1 hour inter-trial interval) for 4 consecutive days: each mouse was placed in the pool, in which the platform had been moved to the opposite quadrant, changing the mouse starting position on each trial. The 5th day, a second probe test (duration, 60 sec) was performed, in which the platform was removed. To assess visual acuity, mice performed a single-trial test in which the platform was moved to a different quadrant and made visible by a flag on top. After each trial, mice were placed on a paper towel for drying and then put back into their home cages. Mice were recorded during each trial and latency to reach the platform was analyzed through Ethovision XT software (Noldus Information Technology).

Fear conditioning: The fear conditioning apparatus (26 x 26 x 27 cm) consisted in a ventilated and sound-attenuated chamber connected to a PC (Panlab Harvard Apparatus). The chamber was provided with a floor composed by 19 steel rods through which a scrambled shock can be delivered, black stainless steel sidewalls and a Plexiglas door. The day of conditioning (Day 1), each mouse, after 2 min of exploration, received 5 consecutive tone-shock pairings at 30 sec inter-trial intervals. The 85-dB tone (conditioned stimulus, CS) lasts 28 sec and was followed by a 0.5 mA shock (unconditioned stimulus, US) for 2 sec. Acetic acid (5%) was used for cleaning in between training sessions. 24 h after (Day 2, context test) mice were re-exposed to the same chamber for 5 min without receiving any stimulus. 24 h later (Day 3, cued test) mice were habituated for 2 min to a different context, i.e. a cylinder with a smooth floor cleaned with ethanol 4%, to provide a different odor. Subsequently, they received 3 tone-shock pairings. Stimuli delivery, data acquisition and analysis were performed automatically using Packwin software (Panlab Harvard Apparatus). The percentage of freezing during sessions was evaluated and was defined as percentage of time the animal was immobile except for breathing.

Preparation of Prime MEA chips with cultured mouse neurons

The external surface of Prime HD-MEA system chips (3Brain, Switzerland) was cleaned with ethanol, and chips were placed into a sterile 10 cm-diameter Petri dish (Corning). Chips plating area was then filled with 70% ethanol for 20 min, rinsed 2-3 times with sterile double-distilled water (DDW), and dried under a laminar hood. Afterwards, chips were coated with a drop of laminin (0.1 mg/ml in DDW; Sigma Aldrich) mixed with a drop of poly-d-lysine (0.1 mg/ml in borate buffer: 100 mM boric acid, 25 mM sodium tetraborate, pH 8.4; Sigma Aldrich), Petri dishes were sealed with parafilm to prevent evaporation, and Petri with chips were incubated overnight at 37 °C and 5% CO_2. The next day, MEA chips were rinsed 4 times with sterile DDW and left to dry under the hood before cell seeding. Dissociated neurons (80 µl, 70000 neurons) were plated on the recording area of chips. After incubation (1h, 37 °C, 5% CO_2), 1.5 mL of complete medium (Neurobasal A, Gibco) supplemented with 1% L-glutamine (Gibco), 4% B27 (Gibco), 1% Glucose, 1% Penicillin-Streptomycin (Gibco) was added. One-third of the medium was replaced with fresh medium twice a week, according to the manufacturer's instructions. Spontaneous extracellular activity of neurons cultured on HD-MEAs was recorded at DIV16-21. On the day of the recordings, MEA chips were moved out of the incubator. After acclimation of 10 min, spontaneous activity was monitored for 20-25 min at RT to allow activity stabilization and recorded during the following 5 min.

Preparation of Arena MEA chips with acute brain slices

Pcdh19 cKO mice (*Pcdh19*$^{fl/x}$ Syn1-Cre) and control littermates (*Pcdh19*$^{fl/x}$) were sacrificed by cervical dislocation and their brain was rapidly removed and placed in an ice-cold cutting solution at pH 7.3, equilibrated with 95% O_2 and 5% CO_2 (cutting solution: mM, 220 sucrose, 2 KCl, 1.3 NaH_2PO_4, 12 $MgSO_4$, 0.2 $CaCl_2$, 10 glucose, 2.6 $NaHCO_3$, 3 kynurenic acid). After removing cerebellum and prefrontal cortex, 400 µm-thick coronal brain slices containing the hippocampus (central region along the antero-posterior axis) were cut using a vibratome (Leica VT1000S). Slices

were incubated at 35°C for 40 min and then at RT for 1 hour in standard aCSF before recordings. Slices were transferred to Arena HD-MEA chips (3Brain, Switzerland).

A square-shaped platinum mesh anchor (custom-made by 3Brain) was placed over the slices to prevent displacement and to ensure uniform contact with the electrodes. Slices were perfused at a rate of ~4.5 mL/min with oxygenated aCSF at RT (22-24°C) for 10 min. Subsequently, Mg^{2+}-free aCSF solution supplemented with 4-Aminopyridine (100 µM, Sigma Aldrich) was used in order to evoke epileptic-like activity. Recordings were performed at RT and slices were continuously perfused at a rate of 4.5 ml/min with aCSF.

Surgery for *in vivo* MEA implant

After 3 days of acclimatization at animal facility, wireless MEAs were implanted in P34-37 mouse brains, in correspondence of limbic system core regions, i.e. the amygdala (Amy), the CA1 hippocampal region, the perirhinal cortex (PRC) and the entorhinal cortex (ERC). To prevent infections, tetracycline was administered with water (50 mg/500 ml) 2 days before the surgery and instruments, working areas, and the stereotaxic apparatus were sterilized. Stereotaxic coordinates were -3.0 mm anterior-posterior and 3.0 mm medial-lateral from bregma, which identify an area on the parietal and interparietal bones overlying the primary and secondary visual cortices. Mice were anesthetized by a gaseous mixture of oxygen (95%) and isoflurane (5%) in the induction chamber for few minutes. The level of anesthesia was checked by testing the mouse response to opportune sensory stimuli usually provoking limb retraction. After induction, anesthesia was maintained throughout the surgical procedure by continuously administering isoflurane through a face mask. The mouse head was shaved down to the level of the descending trapezium and the skin was disinfected. The mouse was placed on a sterilized surgical desk in prone position. The mouse skin was incised from the nasal bones to the occipital bone. The surgical incision was then enlarged over the skull vault. The periosteum was then accurately removed from the bone vault to clear the access to the frontal and parietal left bones. A sterile dressing soaked with physiological solution was placed on the

surgical field and fixed with surgical clips. The mouse, but the head, was then inserted within a plastic multi-perforated sack to avoid contaminations from fur and placed on a warmed surgical bed with temperature regulation at 37.5 °C within the frame of the stereotaxic apparatus. After fixing the mouse head to the apparatus, the bone surface was again cleaned and dried. If serous or bleeding sources were evident, they were dried out by pressing cotton blobs or burned by cauterization. Under surgical microscope control, a bone window for each hemisphere (mm^2: 2x4, medial-lateral x anterior-posterior) was created to expose the meningeal dura mater surface. Bone powders springing off during the drilling procedure were conveniently removed by a pipette connected to a void-pump. One small pilot hole was made to aid for the insertion of a micro-screw (ground electrode). After, the cortical surface was maintained well hydrated and in correct osmotical environment by warmed aCSF. The microelectrodes matrix, previously cleaned by ethanol solution (70%) rinsing and then by immersion in sterile distilled water, was fixed to a sterile clamp mounted on a micro-stepper electronic motor (M228 Stepper-Mike Linear Actuators) on the stereotaxic frame. The motor was initially placed by manual control over the insertion area, in orthogonal position respect to the cortical surface, and slowly lowered toward the cortical surface. Next, the programmable motor was remotely controlled to guide the microelectrode matrix to touch the cortical surface. Finally, an electronically controlled descent step was made to reach the brain areas of interest (2-2.65 mm for PRC and CA1 electrodes, 4-5mm for Amy and ERC electrodes) and the electrode ground wire was fixed to the previously implanted micro-screw. The exposed cortical surface was gently covered with polyethylene glycol 4000, removing the excesses from the bone around. The whole surgical operation area was then covered by dental cement, molded around the plastic basis of the electrodes. The mouse was extracted from the protecting sack, removed from the stereotactic frame and placed in its cage in a warm environment. Gentamycin was spread around the surgical region. Electrophysiological recordings started 3 days after the surgery (recovery period).

SUPPLEMENTARY FIGURES LEGENDS

Supplementary Fig. 1 *Pcdh19* exon 3 excision by Cre recombinase prevents *Pcdh19* expression.

a *Pcdh19* transcripts were assayed by RT-PCR with probes targeting upstream (exon, E 1-2) and downstream (E 4-5, E 5-6) regions of exon 3 in primary neurons prepared from P0 *Pcdh19* floxed mice (*Pcdh19*$^{fl/fl}$ and *Pcdh19*$^{fl/y}$). Neurons were infected at DIV0 with GFP-Cre AAVs. Infected and uninfected (Ctrl) neurons were lysed at DIV10. *Pcdh19* mRNA expression was quantified by $2^{-\Delta\Delta Ct}$ method and normalized on *actin* mRNA levels. Data are shown as means ± SEM (N = 3; Student's t-test, ** $p < 0.01$, *** $p < 0.001$). See also Supplementary Table 7.

b IF on primary neurons as in **a**. Neurons were stained at DIV7 with MAP2 and PCDH19. Scale bar, 20 µm.

c Western blot on primary neurons as in **a**. Neurons were lysed at DIV10. GAPDH was used as loading control.

Supplementary Fig. 2 PCDH19 is expressed in both excitatory and inhibitory neurons and localizes at excitatory and inhibitory synapses.

a Representative IF images of coronal cortical and hippocampal slices from a P23 *Pcdh19* floxed mouse (*Pcdh19*$^{fl/x}$) stained for PCDH19, NeuN, GAD and DAPI, as indicated. The inserts show higher magnification images. Scale bars, 100 µm.

b Representative IF images of primary neurons from P0 *Pcdh19* floxed mice (*Pcdh19*$^{fl/fl}$ and *Pcdh19*$^{fl/y}$) (left) and colocalization analysis (right). Neurons were stained at DIV11 with PCDH19 and the markers of excitatory synapses VGLUT and PSD95. The fluorescence intensity profiles plot refers to the dendrite in the higher magnification images. Scale bars, 10 µm.

Van Steensel's cross correlation function (Pearson's, R, as a function of pixelshift), R and Manders' coefficients (M1, fraction of PCDH19 overlapping with the indicated synaptic markers and M2,

fraction of each synaptic marker overlapping with PCDH19) are shown. N=13 neurons from 3 independent cultures.

c Representative IF images of primary neurons from mice as in **b** (left) and colocalization analysis (right). Neurons were stained at DIV11 with PCDH19 and the proteins of inhibitory synapses VGAT and $GABA_AR$ beta 3 subunit. The fluorescence intensity profiles plot refers to the dendrite in the higher magnification images. Scale bars, 10 µm. Van Steensel's cross correlation function, R, and M1, M2 coefficients are shown. N=15 neurons from 3 independent cultures.

All data (**b, c**) are shown as means ± SEM. See also Supplementary Table 8.

Supplementary Fig. 3 Genotyping strategy and expression of the *Pcdh19* floxed allele.

a Schematic representation of PCR primers annealing on *Pcdh19* WT, floxed and cKO alleles and amplification products length (base pairs, bp: WT 1053, floxed 1224, cKO 379). E = exon.

b Representative results of PCR-based genotyping for *Pcdh19* WT, cKO female (*Pcdh19$^{fl/x}$* Syn1-Cre) and cKO male (*Pcdh19$^{fl/y}$* Syn1-Cre) mice, as indicated. Genomic DNA was extracted from the tail (left panel) or from the cortex (right panel). The *Pcdh19* floxed allele (1224 bp) is converted in the cKO allele (379 bp) selectivity in brain tissue.

c Representative Western blot and relative quantification showing PCDH19 protein expression in the hippocampus of three different groups of female mice: WT, heterozygous *Pcdh19* floxed mice (*Pcdh19$^{fl/x}$*) and homozygous *Pcdh19* floxed mice (*Pcdh19$^{fl/fl}$*). PCDH19 was normalized on GAPDH. Data are shown as means ± SEM (N mice = 5/genotype; one-way ANOVA, n.s.). See also Supplementary Table 9.

Supplementary Fig. 4 Analysis of synapse ultrastructure in *Pcdh19* mosaic mice obtained by Cre AAV injection

a Representative Western blots and relative quantification of PCDH19 expression in the hippocampus of > P90 *Pcdh19* mosaic mice obtained via GFP-2A-Cre AAVs injection of *Pcdh19* floxed mice

(*Pcdh19*$^{fl/fl}$ and *Pcdh19*$^{fl/y}$) (Mosaic) and uninjected WT (Ctrl) mice. PCDH19 expression was normalized on tubulin (N, Ctrl 3, Mosaic 3; Student's t-test, * $p < 0.05$).

b Representative electron micrographs of excitatory synapses on apical dendrites of hippocampal CA1 pyramidal neurons from P50 mice as in **a** (top panel; scale bar, 200 nm)

c Quantification of synapse density and structural features from neurons as in **b** (N mice, Ctrl 3, Mosaic 2; N synapses > 100/genotype. Synapse density was evaluated on 369.0 μm^2 (Ctrl) and 328.0 μm^2 (Mosaic). Mann-Whitney test or Student's t-test, * $p < 0.05$, *** $p < 0.001$, see Supplementary Table 10).

d PSD thickness and vesicle density distribution in excitatory synapses as in **b** (N mice, Ctrl 3, Mosaic 2).

All data (**a, c**) are shown as means ± SEM. See also Supplementary Table 10.

Supplementary Fig. 5 Hippocampal slices from *Pcdh19* mosaic mice at P8-10 and P60-90 display altered network activity.

a Top left panel, representative images of MEA activity maps in pseudocolors, showing firing activity of hippocampal slices from *Pcdh19* mosaic mice (*Pcdh19*$^{fl/x}$ Syn1-Cre, Mosaic) and controls (*Pcdh19*$^{fl/x}$, Ctrl) at P8-10. Top middle panel, representative raster plots showing electrophysiological activity recorded from 100 active channels over 5 min in Ctrl and Mosaic mice. Top right panel, representative traces recorded form a single electrode over 5 min in Ctrl and Mosaic mice. Bottom panels, quantification of MFR (total and by area: CA1, CA3, DG), MBR, MBD, PSB, and SI in Ctrl and Mosaic mice (N slices, Ctrl 8, Mosaic 6, from 6 Ctrl and 3 Mosaic mice; Student's t-test, * $p < 0.05$, *** $p < 0.001$).

b Panels as in **a**, showing data from *Pcdh19* mosaic mice (*Pcdh19*$^{fl/x}$ Syn1-Cre, Mosaic) and controls (*Pcdh19*$^{fl/x}$, Ctrl) at P60-90 (N slices, Ctrl 5, Mosaic 5, from 3 mice/genotype; Student's t-test, * $p < 0.05$, ** $p < 0.01$).

All data (**a-b**) are shown as means ± SEM. See also Supplementary Table 11.

Supplementary Fig. 6 Stereotactic coordinates of the extracellular microelectrodes for *in vivo* recordings.

Top and middle panels, coronal views of the two bilateral recording sites along the anteroposterior axis. Colored vertical bars superimposed on mouse coronal sections drawings [1] indicate groups of microelectrodes targeting brain regions of interests. 4 microelectrodes were positioned in amygdala (Amy), CA1 and entorhinal cortex (ERC), and 3 microelectrodes were positioned in perirhinal cortex (PRC). Bottom panel, length and spacing of the 15 microelectrodes that compose one of the two rows (one for each hemisphere) of the recording matrix (MicroProbes, Gaithersburg, US) connected to the headstage (Omnetics, Minneapolis, US). A, anterior; P, posterior.

Supplementary Fig. 7 *Pcdh19* cKO male mice display mosaic expression of PCDH19.

a Quantification of *Pcdh19* expression in cerebral cortex and in hippocampus from adult (P90) *Pcdh19* cKO male mice (*Pcdh19*$^{fl/y}$ Syn1-Cre, Mosaic) compared to sex-matched control littermates (*Pcdh19*$^{fl/y}$, Ctrl). *Pcdh19* mRNA expression was normalized on *actin* mRNA levels (Cortex: N, Ctrl 4, Mosaic 3; Hippocampus: N, Ctrl 5, Mosaic 3; Student's t-test, * $p < 0.05$, ** $p < 0.01$).

b Representative Western blots and relative quantification of PCDH19 expression in cortex and hippocampus from adult (P90-120) male mice as in **a**. PCDH19 expression was normalized on GAPDH (Cortex: N, Ctrl 8, Mosaic 9; Hippocampus: N, Ctrl 6, Mosaic 7; Student's t-test, *** $p < 0.001$).

c Representative IF images of coronal cortical slices from P30 mice as in **a** stained for PCDH19 and DAPI. The inserts show higher magnification images; arrows indicate representative couples of PCDH19-positive (yellow arrows) and -negative (white arrows) neurons. Scale bar, 100 µm (lower magnification), 20 µm (higher magnification).

d Representative Western blot and relative quantification of PCDH19 protein expression in the hippocampus of three different groups of male mice: WT, *Pcdh19* floxed (*Pcdh19*$^{fl/y}$) and Syn1-Cre

mice. PCDH19 was normalized on GAPDH (N mice, WT 7, *Pcdh19*$^{fl/y}$ 6, Syn1-Cre 6; one-way ANOVA n.s.).

All data (**a, b, d**) are shown as means ± SEM. See also Supplementary Table 12.

Supplementary Fig. 8 LFP power spectral density at low-frequency bands is preserved in *Pcdh19* mosaic mice.

LFP power spectral density in δ (1-4 Hz) and θ (4-8 Hz) bands from limbic system regions of *Pcdh19* mosaic mice (*Pcdh19*$^{fl/y}$ Syn1-Cre, Mosaic) and controls (*Pcdh19*$^{fl/y}$, Ctrl) (N mice, Ctrl 2, Mosaic 2; Student's t-test, n.s.). Data are shown as means ± SEM. See also Supplementary Table 13.

Supplementary Fig. 9 LFP oscillations are affected in hippocampal slices from *Pcdh19* mosaic mice.

a Representative LFP trace before and after lowpass filtering (1-150 Hz). LFP event amplitude and duration are shown.

b-d Representative LFP events (top) and quantification of LFP rate, amplitude, duration and synchronization (bottom) in hippocampal slices from mosaic mice (*Pcdh19*$^{fl/x}$ Syn1-Cre, Mosaic) and controls (*Pcdh19*$^{fl/x}$, Ctrl) at P8-10 (**b**), P18-20 (**c**), and P60-90 (**d**) (P8-10: N slices, Ctrl 12, Mosaic 6, from 8 Ctrl and 4 Mosaic mice; P18-20: N slices, Ctrl 13, Mosaic 8, from 6 Ctrl and 4 Mosaic mice; P60-90: N slices, Ctrl 5, Mosaic 5, from 3 Ctrl and 3 Mosaic mice; Student's t-test, * $p < 0.05$, ** $p < 0.01$).

e-g Power spectral density of LFP bands (Hz, α 10-12, β 12-25, γ 25-100) in hippocampal slices from mice as in **b-d** at P8-10 (**e**), P18-20 (**f**), and P60-90 (**g**) (P8-10: N slices, Ctrl 12, Mosaic 6, from 8 Ctrl and 4 Mosaic mice; P18-20: N slices, Ctrl 13, Mosaic 8 from 6 Ctrl and 4 Mosaic mice; P60-90: N slices, Ctrl 13, Mosaic 8, from 3 Ctrl and 3 Mosaic mice; Student's t-test, * $p < 0.05$, ** $p < 0.01$, *** $p < 0.001$).

All data (**b-g**) are shown as means ± SEM. See also Supplementary Table 14.

SUPPLEMENTARY REFERENCES

1. Paxinos G, Franklin KB. The Mouse Brain in Stereotaxic Coordinates. Second Edi. 2004.

SUPPLEMENTARY TABLES

Supplementary table 1. Mean values, SEM, number of samples and p values of data displayed in Figure 1.

**Fig. 1b** *Pchd19* **mRNA**	**Mean**	**SEM**	**N**	**p value** (Two – tailed unpaired Student's t – test)
Ctrl (Cortex)	8.296	0.755	8	Ctrl vs. Mosaic p < 0.001 (***)
Mosaic (Cortex)	3.656	0.204	6	
Ctrl (Hippocampus)	10.240	0.510	7	Ctrl vs. Mosaic p = 0.001 (**)
Mosaic (Hippocampus)	5.799	0.947	7	

**Fig. 1c** **PCDH19 / GAPDH**	**Mean**	**SEM**	**N**	**p value** (Two – tailed unpaired Student's t – test)
Ctrl (Cortex)	1.000	0.111	7	Ctrl vs. Mosaic p = 0.020 (*)
Mosaic (Cortex)	0.645	0.078	8	
Ctrl (Hippocampus)	1.000	0.090	8	Ctrl vs. Mosaic p = 0.003 (**)
Mosaic (Hippocampus)	0.602	0.059	7	

**Fig. 1e** **Weight (g)**	**Group**	**Mean**	**SEM**	**N**	**p value** (Mixed – effect Two – way ANOVA, *post hoc* FDR)
Week					
3	Ctrl	7.876	0.283	18	Ctrl vs. Mosaic p = 0.041 (*)
	Mosaic	7.165	0.205	20	
4	Ctrl	11.961	0.683	20	Ctrl vs. Mosaic p = 0.021(*)
	Mosaic	9.898	0.432	19	
5	Ctrl	14.762	0.693	16	Ctrl vs. Mosaic p = 0.010 (**)
	Mosaic	12.222	0.389	17	
6	Ctrl	16.664	0.403	22	Ctrl vs. Mosaic p = 0.007 (**)
	Mosaic	14.334	0.512	14	
7	Ctrl	18.075	0.423	18	Ctrl vs. Mosaic p = 0.015 (**)
	Mosaic	16.634	0.290	20	
8	Ctrl	19.049	0.458	17	Ctrl vs. Mosaic p = 0.025 (*)
	Mosaic	17.634	0.377	14	
9	Ctrl	20.661	0.397	11	Ctrl vs. Mosaic p = 0.041 (*)
	Mosaic	19.046	0.673	9	
10	Ctrl	19.913	0.507	12	Ctrl vs. Mosaic p = 0.316 (n.s.)
	Mosaic	19.585	0.351	10	

11	Ctrl	21.125	0.538	8	Ctrl vs. Mosaic p = 0.074 (n.s.)
	Mosaic	19.952	0.491	11	
12	Ctrl	22.049	0.342	17	Ctrl vs. Mosaic p = 0.074 (n.s.)
	Mosaic	21.076	0.508	12	
**Weight (%)**	**Group**	**Mean**	**SEM**	**N**	**p value** (Mixed – effect Two – way ANOVA, *post hoc* FDR)
Week					
3	Ctrl	100.000	3.598	18	Ctrl vs. Mosaic p = 0.044 (*)
	Mosaic	90.971	2.603	20	
4	Ctrl	100.000	5.708	20	Ctrl vs. Mosaic p = 0.021(*)
	Mosaic	82.756	3.609	19	
5	Ctrl	100.000	4.694	16	Ctrl vs. Mosaic p = 0.010 (**)
	Mosaic	82.797	2.633	17	
6	Ctrl	100.000	2.421	22	Ctrl vs. Mosaic p = 0.007 (**)
	Mosaic	86.017	3.071	14	
7	Ctrl	99.910	2.338	18	Ctrl vs. Mosaic p = 0.015 (*)
	Mosaic	92.028	1.608	20	
8	Ctrl	99.910	2.403	17	Ctrl vs. Mosaic p = 0.025 (*)
	Mosaic	92.568	1.981	14	
9	Ctrl	99.910	1.920	11	Ctrl vs. Mosaic p = 0.044 (*)
	Mosaic	92.182	3.259	9	
10	Ctrl	100.000	2.548	12	Ctrl vs. Mosaic p = 0.316 (n.s.)
	Mosaic	98.355	1.764	10	
11	Ctrl	100.000	2.548	8	Ctrl vs. Mosaic p = 0.074 (n.s.)
	Mosaic	94.446	2,323	11	
12	Ctrl	99.910	1.552	17	Ctrl vs. Mosaic p = 0.074 (n.s.)
	Mosaic	95.585	2.305	12	

**Fig. 1f** **Weight gain (%)**	**Group**	**Mean**	**SEM**	**N**	**p value** (Mixed – effect Two – way ANOVA, *post hoc* FDR)
Week					
3 – 4	Ctrl	52.766	5.026	16	Ctrl vs. Mosaic p = 0.003 (**)
	Mosaic	38.254	3.083	19	
4 – 5	Ctrl	27.217	4.655	14	Ctrl vs. Mosaic p = 0.667 (n.s.)
	Mosaic	22.931	4.750	16	
5 – 6	Ctrl	14.121	3.179	16	Ctrl vs. Mosaic p = 0.506 (n.s.)
	Mosaic	20.289	3.712	11	
6 – 7	Ctrl	7.395	1.222	18	Ctrl vs. Mosaic p = 0.427 (n.s.)
	Mosaic	14.269	2.532	14	
7 – 8	Ctrl	6.615	0.820	16	Ctrl vs. Mosaic p = 0.933 (n.s.)
	Mosaic	8.588	0.777	14	
8 – 9	Ctrl	3.520	1.109	7	Ctrl vs. Mosaic p = 0.933 (n.s.)
	Mosaic	4.380	0.984	6	
9 – 10	Ctrl	-0.778	0.571	3	Ctrl vs. Mosaic p = 0.933 (n.s.)
	Mosaic	2.243	1.535	5	
10 – 11	Ctrl	2.518	0	1	Ctrl vs. Mosaic p = 0.933 (n.s.)
	Mosaic	2.518	1.463	8	
11 – 12	Ctrl	3.877	0.277	7	Ctrl vs. Mosaic

	Mosaic	3.889	1.351	8	p = 0.933 (n.s.)

**Weight gain (g)**	**Mean**	**SEM**	**N**	**p value** (Two – tailed unpaired Student's t – test)
Ctrl	13.77	0.269	13	Ctrl vs. Mosaic p = 0.894 (n.s.)
Mosaic	13.84	0.445	12	

**Fig. 1g** **Stage / time (min)**	**Group**	**Mean**	**SEM**	**N**	**p value** (Two – way ANOVA, *post hoc* FDR)
2 min	Ctrl	0.625	0.183	8	Ctrl vs. Mosaic p = 0.126 (n.s.)
	Mosaic	1.375	0.375	8	
3 min	Ctrl	0.750	0.164	8	Ctrl vs. Mosaic p = 0.068 (n.s.)
	Mosaic	2.125	0.549	8	
4 min	Ctrl	0.875	0.125	8	Ctrl vs. Mosaic p = 0.049 (*)
	Mosaic	2.375	0.532	8	
5 min	Ctrl	0.875	0.125	8	Ctrl vs. Mosaic p = 0.021 (*)
	Mosaic	2.875	0.549	8	
6 min	Ctrl	0.875	0.125	8	Ctrl vs. Mosaic p = 0.016 (*)
	Mosaic	3.250	0.491	8	
7 min	Ctrl	1.125	0.295	8	Ctrl vs. Mosaic p = 0.016 (*)
	Mosaic	3.375	0.532	8	
8 min	Ctrl	1.375	0.324	8	Ctrl vs. Mosaic p = 0.021 (*)
	Mosaic	3.375	0.532	8	
9 min	Ctrl	1.125	0.295	8	Ctrl vs. Mosaic p = 0.016 (*)
	Mosaic	3.625	0.596	8	
10 min	Ctrl	1.125	0.295	8	Ctrl vs. Mosaic p = 0.016 (*)
	Mosaic	3.500	0.463	8	
11 min	Ctrl	1.375	0.420	8	Ctrl vs. Mosaic p = 0.016 (*)
	Mosaic	3.375	0.420	8	
12 min	Ctrl	1.375	0.420	8	Ctrl vs. Mosaic p = 0.028 (*)
	Mosaic	3.250	0.491	8	
13 min	Ctrl	1.875	0.479	8	Ctrl vs. Mosaic p = 0.077 (n.s.)
	Mosaic	3.375	0.532	8	
14 min	Ctrl	1.625	0.420	8	Ctrl vs. Mosaic p = 0.042 (*)
	Mosaic	3.500	0.567	8	
15 min	Ctrl	2.000	0.463	8	Ctrl vs. Mosaic p = 0.067 (n.s.)
	Mosaic	3.500	0.463	8	
16 min	Ctrl	2.000	0.535	8	Ctrl vs. Mosaic p = 0.084 (n.s.)
	Mosaic	3.375	0.420	8	
17 min	Ctrl	2.125	0.549	8	Ctrl vs. Mosaic p = 0.072 (n.s.)
	Mosaic	3.500	0.267	8	
18 min	Ctrl	2.125	0.549	8	Ctrl vs. Mosaic p = 0.016 (*)
	Mosaic	4.000	0.378	8	
19 min	Ctrl	2.250	0.412	8	Ctrl vs. Mosaic p = 0.308 (n.s.)
	Mosaic	3.000	0.627	8	
20 min	Ctrl	2.000	0.535	8	Ctrl vs. Mosaic p = 0.558 (n.s.)
	Mosaic	2.250	0.526	8	
21 min	Ctrl	2.125	0.515	8	Ctrl vs. Mosaic p = 0.474 (n.s.)
	Mosaic	2.500	0.500	8	
22 min	Ctrl	1.625	0.375	8	Ctrl vs. Mosaic p = 0.157 (n.s.)

	Mosaic	2.625	0.532	8	
23 min	Ctrl	1.500	0.423	8	Ctrl vs. Mosaic p = 0.134 (n.s.)
	Mosaic	2.625	0.532	8	
24 min	Ctrl	1.375	0.324	8	Ctrl vs. Mosaic p = 0.359 (n.s.)
	Mosaic	1.875	0.515	8	
25 min	Ctrl	2.125	0.515	8	Ctrl vs. Mosaic p = 0.635 (n.s.)
	Mosaic	2.000	0.598	8	
26 min	Ctrl	1.500	0.423	8	Ctrl vs. Mosaic p = 290 (n.s.)
	Mosaic	2.250	0.559	8	
27 min	Ctrl	1.750	0.453	8	Ctrl vs. Mosaic p = 0.403 (n.s.)
	Mosaic	2.250	0.559	8	
28 min	Ctrl	1.625	0.460	8	Ctrl vs. Mosaic p = 0.288 (n.s.)
	Mosaic	2.375	0.498	8	
29 min	Ctrl	1.625	0.420	8	Ctrl vs. Mosaic p = 0.133 (n.s.)
	Mosaic	2.625	0.420	8	
30 min	Ctrl	1.500	0.420	8	Ctrl vs. Mosaic p = 0.344 (n.s.)
	Mosaic	2.000	0.378	8	

**Latency to onset (min)**	**Mean**	**SEM**	**N**	**p value** (Two – tailed unpaired Student's t – test)
Ctrl	15.43	0.00	1	Ctrl vs. Mosaic p = 0.346 (n.s.)
Mosaic	11.08	3.87	6	

Supplementary table 2. Mean values, SEM, number of samples and p values of data displayed in Figure 2.

**Fig. 2a** **Horizontal movements (n)**	**Mean**	**SEM**	**N**	**p value** (Two – tailed unpaired Student's t – test)
Ctrl	1217	92.26	11	Ctrl vs. Mosaic p = 0.206 (n.s.)
Mosaic	1395	100.5	10	
**Vertical movements (n)**	**Mean**	**SEM**	**N**	**p value** (Two – tailed unpaired Student's t – test)
Ctrl	150.3	14.81	11	Ctrl vs. Mosaic p = 0.340 (n.s.)
Mosaic	170.0	13.52	10	

**Fig. 2b** **Events > 25 sec (n)**	**Mean**	**SEM**	**N**	**p value** (Two – tailed unpaired Student's t – test)
Ctrl	0.400	0.245	5	Ctrl vs. Mosaic p = 0.033 (*)
Mosaic	1.500	0.342	6	
**Duration (s)**	**Mean**	**SEM**	**N**	**p value** (Two – tailed unpaired Student's t – test)
Ctrl	60.83	14.46	6	Ctrl vs. Mosaic p = 0.187 (n.s.)
Mosaic	116.7	36.63	6	

**Fig. 2c**	**Group**	**Mean**	**SEM**	**N**	

**Index** (N – F) / (N + F)					**p value** (Two – way ANOVA)
Time					
5 min	Ctrl	0.199	0.068	10	Ctrl vs. Mosaic p > 0.05 (n.s.)
	Mosaic	0.172	0.070	10	
120 min	Ctrl	0.240	0.055	10	
	Mosaic	0.189	0.070	10	
24 h	Ctrl	0.249	0.059	10	
	Mosaic	0.267	0.056	10	

**Fig. 2d** **Escape latency (s)**	**Mean**	**SEM**	**N**	
Acquisition phase				**p value** (One – way ANOVA, *post hoc* FDR)
Ctrl				
1°day	30.79	4.342	11	1°day vs 2°day p = 0.001 (##)<br>1°day vs 3°day p = 0.001 (##)<br>1°day vs 4°day p = 0.009 (##)
2°day	15.01	3.601	11	
3°day	10.34	2.009	11	
4°day	13.25	3.537	11	
Mosaic				
1°day	27.12	4.856	10	1°day vs 2°day p = 0.464 (n.s.)<br>1°day vs 3°day p = 0.082 (n.s.)<br>1°day vs 4°day p = 0.013 (§)
2°day	24.00	4.383	10	
3°day	15.82	3.092	10	
4°day	10.69	2.144	10	
				**p value** (Two – way ANOVA, *post hoc* FDR)
				1°day Ctrl vs. Mosaic p = 0.609 (n.s.)<br>2°day Ctrl vs. Mosaic p = 0.329 (n.s.)<br>3°day Ctrl vs. Mosaic p = 0.329 (n.s.)<br>4°day Ctrl vs. Mosaic p = 0.609 (n.s.)
Probe				**p value** (Two – tailed unpaired Student's t – test)
Ctrl	10.89	3.84	11	Ctrl vs. Mosaic p = 0.797 (n.s.)
Mosaic	9.672	2.62	10	
Reversal phase				**p value** (One – way ANOVA, *post hoc* FDR)
Ctrl				
1°day	18.32	3.377	11	1°day vs 2°day p = 0.004 (##)<br>1°day vs 3°day p = 0.004 (##)<br>1°day vs 4°day p = 0.004 (##)
2°day	7.606	1.141	11	
3°day	5.585	0.759	11	
4°day	5.195	0.635	11	
Mosaic				
1°day	22.03	3.023	10	1°day vs 2°day p < 0.0001 (§§§)<br>1°day vs 3°day p = 0.003 (§§)<br>1°day vs 4°day p = 0.002 (§§)
2°day	10.82	2.719	10	
3°day	10.48	2.109	10	
4°day	8.037	1.525	10	
				**p value** (Two – way ANOVA, *post hoc* FDR)

	1°day Ctrl vs. Mosaic p = 0.444 (n.s.)<br>2°day Ctrl vs. Mosaic p = 0.415 (n.s.)<br>3°day Ctrl vs. Mosaic p = 0.214 (n.s.)<br>4°day Ctrl vs. Mosaic p = 0.233 (n.s.)

Probe				**p value**<br>(Two – tailed unpaired Student's t – test)
Ctrl	3.520	1.217	11	Ctrl vs. Mosaic p = 0.115 (n.s.)
Mosaic	10.18	4.021	10	

**Fig. 2e**<br><br>**Freezing time (s)**	**Group**	**Mean**	**SEM**	**N**	
Conditioning					**p value**<br>(Two – way ANOVA)
1 min	Ctrl	40.31	3.30	10	Ctrl vs. Mosaic p > 0.05 (n.s.)
	Mosaic	40.18	4.40	9	
2 min	Ctrl	42.12	3.82	10	
	Mosaic	40.28	3.72	9	
3 min	Ctrl	44.77	2.46	10	
	Mosaic	44.53	3.20	9	
4 min	Ctrl	50.62	1.76	10	
	Mosaic	50.14	2.21	9	
5 min	Ctrl	51.68	1.35	10	
	Mosaic	54.10	1.47	9	
6min	Ctrl	53.57	2.87	10	
	Mosaic	52.08	1.70	9	
7 min	Ctrl	55.97	1.41	10	
	Mosaic	55.49	2.74	9	
Context					**p value**<br>(Two – way ANOVA, *post hoc* FDR)
1 min	Ctrl	45.12	3.71	10	Ctrl vs. Mosaic p = 0.099 (n.s.)
	Mosaic	41.04	3.82	9	
2 min	Ctrl	52.67	3.23	10	Ctrl vs. Mosaic p = 0.013 (*)
	Mosaic	45.69	3.19	9	
3 min	Ctrl	55.60	3.07	10	Ctrl vs. Mosaic p = 0.007 (**)
	Mosaic	45.74	3.18	9	
4 min	Ctrl	55.25	2.35	10	Ctrl vs. Mosaic p = 0.034 (*)
	Mosaic	47.39	4.74	9	
5 min	Ctrl	54.51	1.98	10	Ctrl vs. Mosaic p = 0.013 (*)
	Mosaic	44.27	3.63	9	
Cued					**p value**<br>(Two – way ANOVA)
1 min	Ctrl	32.93	1.97	10	Ctrl vs. Mosaic p > 0.05 (n.s.)
	Mosaic	31.60	3.87	9	
2 min	Ctrl	39.59	3.12	10	
	Mosaic	41.59	3.07	9	
3 min	Ctrl	46.31	3.21	10	
	Mosaic	49.40	3.23	9	
4 min	Ctrl	47.41	2.25	10	

	Mosaic	48.91	3.60	9	
5 min	Ctrl	46.72	2.52	10	
	Mosaic	47.02	3.59	9	

**Fig. 2f**<br><br>**fEPSP slope**	**Mean**	**SEM**	**N**	**p value**<br>(Two – tailed unpaired Student's t – test)
First 10 min				
Ctrl	182.8	11.12	10	Ctrl vs. Mosaic p = 0.002 (**)
Mosaic	135.3	6.078	10	
Last 10 min				
Ctrl	190.3	18.28	10	Ctrl vs. Mosaic p = 0.010 (**)
Mosaic	130.2	9.918	10	

**Fig. 2g**<br><br>**fEPSP PPR**	**Mean**	**SEM**	**N**	**p value**<br>(Two – tailed unpaired Student's t – test)
Ctrl	1.696	0.072	11	Ctrl vs. Mosaic p = 0.040 (*)
Mosaic	1.483	0.063	10	

**Fig. 2h**<br><br>**Synapse density ($N / \mu m^3$)**	**Mean**	**SEM**	**N**	**p value**<br>(Two – tailed unpaired Student's t – test)
Ctrl	1.955	0.111	30	Ctrl vs. Mosaic p = 0.005 (**)
Mosaic	1.523	0.096	30	
**PSD thickness (nm)**	**Mean**	**SEM**	**N**	**p value**<br>(Mann – Whitney test)
Ctrl	66.09	2.371	53	Ctrl vs. Mosaic p <0.001 (***)
Mosaic	50.00	1.806	56	
**PSD length (nm)**	**Mean**	**SEM**	**N**	**p value**<br>(Mann – Whitney test)
Ctrl	266.7	10.74	53	Ctrl vs. Mosaic p = 0.019 (*)
Mosaic	304.2	11.72	56	
**SAL (nm)**	**Mean**	**SEM**	**N**	**p value**<br>(Mann – Whitney test)
Ctrl	253.8	13.81	52	Ctrl vs. Mosaic p = 0.001 (**)
Mosaic	306.7	11.39	53	
**Spine head area (μm^2)**	**Mean**	**SEM**	**N**	**p value**<br>(Two – tailed unpaired Student's t – test)
Ctrl	0.137	0.023	53	Ctrl vs. Mosaic p = 0.780 (n.s.)
Mosaic	0.112	0.007	53	

Presynaptic area (μm^2)	Mean	SEM	N	p value (Mann – Whitney test)
Ctrl	0.191	0.017	52	Ctrl vs. Mosaic p = 0.084 (n.s.)
Mosaic	0.227	0.017	53	
**Vesicle density (N / μm^2)**	**Mean**	**SEM**	**N**	**p value** (Mann – Whitney test)
Ctrl	417.9	21.78	53	Ctrl vs. Mosaic p < 0.001 (***)
Mosaic	310.2	19.73	53	

Distribution of PSD thickness (nm)	Ctrl, %	Mosaic, %	p value (Comparison of Z-score, Mann-Whitney test)
10	0.00	1.79	Ctrl vs. Mosaic p = 0.742 (n.s.)
20	0.00	0.00	
30	0.00	7.14	
40	7.55	26.79	
50	18.87	37.50	
60	26.42	12.50	
70	24.53	10.71	
80	13.21	1.79	
90	3.77	1.79	
100	1.89	0.00	
110	1.89	0.00	
120	0.00	0.00	
130	1.89	0.00	
140	0.00	0.00	
**Distribution of vesicle density (N / μm^2)**	**Ctrl, %**	**Mosaic, %**	**p value** (Comparison of Z-score, Two – tailed unpaired Student's t – test)
100	3.77	5.56	Ctrl vs. Mosaic p = 0.986 (n.s.)
200	9.43	35.19	
300	22.64	24.07	
400	28.30	14.81	
500	16.98	9.26	
600	7.55	9.26	
700	9.43	0.00	
800	0.00	1.85	
900	1.89	0.00	

Fig. 2i Pre-synaptic area (μm^2)	Mean	SEM	N	p value (Two – tailed unpaired Student's t – test)

Ctrl	0.238	0.036	20	Ctrl vs. Mosaic p = 0.122 (n.s.)
Mosaic	0.347	0.061	18	
**Vesicle density ($N / \mu m^2$)**	**Mean**	**SEM**	**N**	**p value** (Two – tailed unpaired Student's t – test)
Ctrl	257.2	29.64	20	Ctrl vs. Mosaic p = 0.044 (*)
	185.4	15.1	18	

**Distribution of vesicle density ($N / \mu m^2$)**	**Ctrl, %**	**Mosaic, %**	**p value** (Comparison of Z-score, Two – tailed unpaired Student's t – test)
100	20.00	33.33	Ctrl vs. Mosaic p = 0.114 (n.s.)
200	40.00	61.11	
300	20.00	0.00	
400	15.00	5.56	
500	0.00	0.00	
600	5.00	0.00	
700	0.00	0.00	

Supplementary table 3. Mean values, SEM, number of samples and p values of data displayed in Figure 3.

**Fig. 3b** **AP frequency (Hz)**	**DGGCs**	**Mean**	**SEM**	**N**	**p value** (Two – way ANOVA, *post hoc* FDR)
0 pA	PCDH19 +	0.000	0	11	PCDH19 + vs. PCDH19 – p = 0.453 (n.s.)
	PCDH19 -	0.625	0.34	16	
10 pA	PCDH19 +	0.546	0.55	11	PCDH19 + vs. PCDH19 – p = 0.173 (n.s.)
	PCDH19 -	3.500	0.92	16	
20 pA	PCDH19 +	1.727	1.36	11	PCDH19 + vs. PCDH19 – p = 0.048 (*)
	PCDH19 -	6.938	1.27	16	
30 pA	PCDH19 +	3.182	1.56	11	PCDH19 + vs. PCDH19 – p = 0.026 (*)
	PCDH19 -	9.500	1.53	16	
40 pA	PCDH19 +	5.000	2	11	PCDH19 + vs. PCDH19 – p = 0.019 (*)
	PCDH19 -	12.13	1.63	16	
50 pA	PCDH19 +	6.364	2.20	11	PCDH19 + vs. PCDH19 – p = 0.019 (*)
	PCDH19 -	13.25	1.75	16	
60 pA	PCDH19 +	7.727	2.21	11	PCDH19 + vs. PCDH19 – p = 0.019 (*)
	PCDH19 -	14.69	1.91	16	
70 pA	PCDH19 +	9.182	2.22	11	PCDH19 + vs. PCDH19 – p = 0.026 (*)
	PCDH19 -	15.56	1.98	16	
80 pA	PCDH19 +	10.36	2.09	11	PCDH19 + vs. PCDH19 – p = 0.030 (n.s.)
	PCDH19 -	16.38	2.03	16	
90 pA	PCDH19 +	11.00	2.09	11	PCDH19 + vs. PCDH19 – p = 0.026 (*)
	PCDH19 -	17.25	1.98	16	
100 pA	PCDH19 +	10.55	1.75	11	PCDH19 + vs. PCDH19 – p = 0.019 (*)
	PCDH19 -	17.81	2.09	16	
110 pA	PCDH19 +	10.64	1.70	11	PCDH19 + vs. PCDH19 –

	PCDH19 -	17.88	2.08	16	p = 0.019 (*)
120 pA	PCDH19 +	10.00	1.49	11	PCDH19 + vs. PCDH19 - p = 0.019 (*)
	PCDH19 -	17.44	2.19	16	
130 pA	PCDH19 +	10.00	1.40	11	PCDH19 + vs. PCDH19 - p = 0.019 (*)
	PCDH19 -	17.69	2.22	16	
140 pA	PCDH19 +	9.364	1.16	11	PCDH19 + vs. PCDH19 - p = 0.019 (*)
	PCDH19 -	17.75	2.36	16	
150 pA	PCDH19 +	9.818	1.37	11	PCDH19 + vs. PCDH19 - p = 0.019 (*)
	PCDH19 -	17.50	2.20	16	
160 pA	PCDH19 +	8.727	1.280	11	PCDH19 + vs. PCDH19 - p = 0.019 (*)
	PCDH19 -	17.125	2.219	16	

**Fig. 3c** **Input resistance (mΩ)**	**Mean**	**SEM**	**N**	**p value** (Two – tailed unpaired Student's t – test)
PCDH19 +	485.9	57.76	11	PCDH19 + vs. PCDH19 - p = 0.163 (n.s.)
PCDH19 -	590.4	45.36	16	
**RMP (mV)**	**Mean**	**SEM**	**N**	**p value** (Two – tailed unpaired Student's t – test)
PCDH19 +	-68.84	2.554	11	PCDH19 + vs. PCDH19 - p = 0.001 (**)
PCDH19 -	-57.22	1.921	16	
**Rheobase (pA)**	**Mean**	**SEM**	**N**	**p value** (Two – tailed unpaired Student's t – test)
PCDH19 +	41.82	6.441	11	PCDH19 + vs. PCDH19 - p < 0.001 (***)
PCDH19 -	13.75	2.720	16	

**Fig. 3d** **Instantaneous frequency (Hz) at 150 pA step**		**Mean**	**SEM**	**N**	**p value** (Two – way ANOVA, *post hoc* FDR)
0.1 sec	PCDH19 +	2.45	0.41	11	PCDH19 + vs. PCDH19 - p = 0.055 (n.s.)
	PCDH19 -	3.313	0.25	16	
0.2 sec	PCDH19 +	1.45	0.21	11	PCDH19 + vs. PCDH19 - p = 0.055 (n.s.)
	PCDH19 -	2.313	0.24	16	
0.3 sec	PCDH19 +	1.10	0.21	11	PCDH19 + vs. PCDH19 - p = 0.055 (n.s.)
	PCDH19 -	1.875	0.24	16	
0.4 sec	PCDH19 +	0.91	0.25	11	PCDH19 + vs. PCDH19 - p = 0.055 (n.s.)
	PCDH19 -	1.688	0.30	16	
0.5 sec	PCDH19 +	0.82	0.23	11	PCDH19 + vs. PCDH19 - p = 0.078 (n.s.)
	PCDH19 -	1.5	0.27	16	
0.6 sec	PCDH19 +	0.73	0.24	11	PCDH19 + vs. PCDH19 - p = 0.055 (n.s.)
	PCDH19 -	1.563	0.22	16	
0.7 sec	PCDH19 +	0.73	0.19	11	PCDH19 + vs. PCDH19 - p = 0.075 (n.s.)
	PCDH19 -	1.438	0.26	16	
0.8 sec	PCDH19 +	0.55	0.16	11	PCDH19 + vs. PCDH19 - p = 0.055 (n.s.)
	PCDH19 -	1.563	0.20	16	

0.9 sec	PCDH19 +	0.73	0.19	11	PCDH19 + vs. PCDH19 - p = 0.173 (n.s.)
	PCDH19 -	1.25	0.28	16	
1.0 sec	PCDH19 +	0.64	0.15	11	PCDH19 + vs. PCDH19 - p = 0.185 (n.s.)
	PCDH19 -	1.125	0.22	16	

**Exponential decay slope**	**Mean**	**SEM**	**N**	**p value** (Two – tailed unpaired Student's t – test)
PCDH19 +	5.932	1.345	8	PCDH19 + vs. PCDH19 - p = 0.712 (n.s.)
PCDH19 -	6.555	0.982	15	

**Fig. 3e** **AP threshold (mV)**	**Mean**	**SEM**	**N**	**p value** (Two – tailed unpaired Student's t – test)
PCDH19 +	-37.45	1.790	11	PCDH19 + vs. PCDH19 - p = 0.578 (n.s.)
PCDH19 -	-38.88	1.698	16	
**AP amplitude (mV)**	**Mean**	**SEM**	**N**	**p value** (Two – tailed unpaired Student's t – test)
PCDH19 +	93.33	2.532	11	PCDH19 + vs. PCDH19 - p = 0.921 (n.s.)
PCDH19 -	93.69	2.498	16	
**AP half - width (ms)**	**Mean**	**SEM**	**N**	**p value** (Two – tailed unpaired Student's t – test)
PCDH19 +	1.282	0.042	11	PCDH19 + vs. PCDH19 - p = 0.321 (n.s.)
PCDH19 -	1.363	0.059	16	
**AP maximum rise slope (mV / ms)**	**Mean**	**SEM**	**N**	**p value** (Two – tailed unpaired Student's t – test)
PCDH19 +	253.9	11.65	11	PCDH19 + vs. PCDH19 - p = 0.552 (n.s.)
PCDH19 -	240.5	16.42	16	
**AP maximum decay slope (mV / ms)**	**Mean**	**SEM**	**N**	**p value** (Two – tailed unpaired Student's t – test)
PCDH19 +	67.08	2.433	11	PCDH19 + vs. PCDH19 - p = 0.317 (n.s.)
PCDH19 -	62.95	2.895	16	

**Fig. 3e** **fAHP (mV)**	**Mean**	**SEM**	**N**	**p value** (Two – tailed unpaired Student's t – test)
PCDH19 +	19.45	1.565	11	PCDH19 + vs. PCDH19 - p = 0.259 (n.s.)
PCDH19 -	16.76	1.593	16	

**Fig. 3f**	**Group**	**Mean**	**SEM**	**N**	

**Current density (pA / pF)**					**p value** (Two – way ANOVA)
**Outward currents**					
-75 mV	PCDH19 +	0.000	0.000	10	
	PCDH19 -	0.000	0.000	15	
-70 mV	PCDH19 +	0.000	0.000	10	
	PCDH19 -	0.000	0.000	15	
-65 mV	PCDH19 +	0.000	0.000	10	
	PCDH19 -	0.000	0.000	15	
-60 mV	PCDH19 +	0.082	0.056	10	
	PCDH19 -	0.000	0.000	15	
-55 mV	PCDH19 +	0.829	0.198	10	
	PCDH19 -	0.430	0.145	15	
-50 mV	PCDH19 +	1.392	0.253	10	
	PCDH19 -	1.825	0.487	15	
-45 mV	PCDH19 +	2.777	0.625	10	
	PCDH19 -	3.265	0.748	15	
-40 mV	PCDH19 +	6.336	1.094	10	
	PCDH19 -	6.151	1.024	15	
-35 mV	PCDH19 +	9.056	1.375	10	
	PCDH19 -	10.729	1.242	15	
-30 mV	PCDH19 +	12.262	1.567	10	
	PCDH19 -	14.149	1.456	15	
-25 mV	PCDH19 +	16.830	0.676	10	
	PCDH19 -	17.314	1.801	15	
-20 mV	PCDH19 +	19.869	0.834	10	
	PCDH19 -	21.535	1.663	15	
-15 mV	PCDH19 +	23.064	1.020	10	
	PCDH19 -	25.197	1.946	15	
-10 mV	PCDH19 +	26.217	1.170	10	
	PCDH19 -	29.104	2.219	15	
-5 mV	PCDH19 +	30.018	1.486	10	
	PCDH19 -	33.277	2.457	15	PCDH19 + vs PCDH19 – $p > 0.05$ (n.s.)
0 mV	PCDH19 +	34.066	1.930	10	
	PCDH19 -	38.073	2.557	15	
5 mV	PCDH19 +	38.315	2.339	10	
	PCDH19 -	42.889	2.889	15	
10 mV	PCDH19 +	42.707	2.707	10	
	PCDH19 -	48.457	3.186	15	
15 mV	PCDH19 +	47.841	3.447	10	
	PCDH19 -	53.759	3.291	15	
20 mV	PCDH19 +	52.316	3.956	10	
	PCDH19 -	59.925	3.874	15	
25 mV	PCDH19 +	56.975	4.486	10	
	PCDH19 -	65.502	4.207	15	
30 mV	PCDH19 +	62.202	5.222	10	
	PCDH19 -	72.835	4.702	15	
35 mV	PCDH19 +	66.914	5.824	10	
	PCDH19 -	78.062	5.181	15	
40 mV	PCDH19 +	72.206	6.444	10	

	PCDH19 -	83.605	5.459	15	
45 mV	PCDH19 +	77.568	7.228	10	
	PCDH19 -	89.209	5.734	15	
50 mV	PCDH19 +	83.616	8.138	10	
	PCDH19 -	95.693	6.314	15	
55 mV	PCDH19 +	88.670	8.793	10	
	PCDH19 -	101.787	6.657	15	
60 mV	PCDH19 +	94.329	9.758	10	
	PCDH19 -	113.901	7.163	15	
65 mV	PCDH19 +	99.941	10.518	10	
	PCDH19 -	113.901	7.646	15	
70 mV	PCDH19 +	106.109	11.567	10	
	PCDH19 -	119.256	8.408	15	
75 mV	PCDH19 +	111.948	12.444	10	
	PCDH19 -	124.491	9.423	15	
**Inward currents**					
-75 mV	PCDH19 +	0.000	0.000	10	PCDH19 + vs. PCDH19 – p > 0.05 (n.s.)
	PCDH19 -	0.000	0.000	14	
-70 mV	PCDH19 +	0.000	0.000	10	
	PCDH19 -	0.000	0.000	14	
-65 mV	PCDH19 +	0.000	0.000	10	
	PCDH19 -	0.000	0.000	14	
-60 mV	PCDH19 +	0.000	0.000	10	
	PCDH19 -	0.000	0.000	14	
-55 mV	PCDH19 +	0.000	0.000	10	
	PCDH19 -	-2.239	2.239	14	
-50 mV	PCDH19 +	0.000	0.000	10	
	PCDH19 -	-44.188	21.227	14	
-45 mV	PCDH19 +	-27.480	17.185	10	
	PCDH19 -	-58.838	20.987	14	
-40 mV	PCDH19 +	-91.611	23.809	10	
	PCDH19 -	-105.529	20.436	14	
-35 mV	PCDH19 +	-97.166	19.770	10	
	PCDH19 -	-134.124	13.729	14	
-30 mV	PCDH19 +	-101.949	18.393	10	
	PCDH19 -	-135.521	10.547	14	
-25 mV	PCDH19 +	-105.352	10.806	10	
	PCDH19 -	-124.244	9.983	14	
-20 mV	PCDH19 +	-98.490	10.188	10	
	PCDH19 -	-118.320	9.279	14	
-15 mV	PCDH19 +	-91.690	9.519	10	
	PCDH19 -	-110.345	8.739	14	
-10 mV	PCDH19 +	-85.054	8.738	10	
	PCDH19 -	-102.623	8.218	14	
-5 mV	PCDH19 +	-78.369	8.052	10	
	PCDH19 -	-94.680	7.624	14	
0 mV	PCDH19 +	-71.621	7.396	10	
	PCDH19 -	-86.713	7.097	14	
5 mV	PCDH19 +	-64.995	6.580	10	
	PCDH19 -	-78.600	6.521	14	
10 mV	PCDH19 +	-58.335	5.920	10	
	PCDH19 -	-70.935	5.923	14	

15 mV	PCDH19 +	-51.736	5.220	10	
	PCDH19 -	-62.840	5.298	14	
20 mV	PCDH19 +	-45.005	4.541	10	
	PCDH19 -	-55.243	4.757	14	
25 mV	PCDH19 +	-38.509	3.841	10	
	PCDH19 -	-47.317	4.167	14	
30 mV	PCDH19 +	-31.847	3.199	10	
	PCDH19 -	-39.565	3.613	14	
35 mV	PCDH19 +	-25.457	2.600	10	
	PCDH19 -	-31.886	3.088	14	
40 mV	PCDH19 +	-19.135	2.067	10	
	PCDH19 -	-24.313	2.557	14	
45 mV	PCDH19 +	-12.921	1.811	10	
	PCDH19 -	-16.731	2.050	14	
50 mV	PCDH19 +	-6.916	1.598	10	
	PCDH19 -	-9.531	1.596	14	
55 mV	PCDH19 +	-2.560	0.909	10	
	PCDH19 -	-3.468	0.811	14	
60 mV	PCDH19 +	-0.167	0.167	10	
	PCDH19 -	-0.016	0.016	14	
65 mV	PCDH19 +	0.000	0.000	10	
	PCDH19 -	0.000	0.000	14	
70 mV	PCDH19 +	0.000	0.000	10	
	PCDH19 -	0.000	0.000	14	
75 mV	PCDH19 +	0.000	0.000	10	
	PCDH19 -	0.000	0.000	14	

**Capacitance (pF)**	**Mean**	**SEM**	**N**	**p value** (Two – tailed unpaired Student's t – test)
Ctrl	52.40	7.334	10	Ctrl vs Mosaic p = 0.432 (n.s.)
Mosaic	46.29	3.803	14	

**I / I max**	**Group**	**Mean**	**SEM**	**N**	**p value** (Two – way ANOVA)
-60 mV	PCDH19 +	0.000	0.000	10	PCDH19 + vs. PCDH19 – p > 0.05 (n.s.)
	PCDH19 -	0.000	0.000	14	
-55 mV	PCDH19 +	0.000	0.000	10	
	PCDH19 -	0.017	0.017	14	
-50 mV	PCDH19 +	0.000	0.000	10	
	PCDH19 -	0.286	0.125	14	
-45 mV	PCDH19 +	0.171	0.104	10	
	PCDH19 -	0.369	0.123	14	
-40 mV	PCDH19 +	0.616	0.152	10	
	PCDH19 -	0.653	0.111	14	
-35 mV	PCDH19 +	0.672	0.129	10	
	PCDH19 -	0.866	0.050	14	
-30 mV	PCDH19 +	0.701	0.119	10	
	PCDH19 -	0.867	0.019	14	
-25 mV	PCDH19 +	0.852	0.030	10	
	PCDH19 -	0.812	0.017	14	

**I / I max**	**Mean**	**SEM**	**N**	**p value**

				(One – sample t test)
PCDH19 –	0.286	0.125	14	p = 0.040 (*)

**Fig. 3g**<br><br>**Amplitude (pA)**	**Mean**	**SEM**	**N**	**p value**<br>(Two – tailed unpaired Student's t – test)
Ctrl	9.630	0.597	10	Ctrl vs. Mosaic p = 0.522 (n.s.)
Mosaic	10.39	1.031	9	
**Area (pA * ms)**	**Mean**	**SEM**	**N**	**p value**<br>(Two – tailed unpaired Student's t – test)
Ctrl	78.87	5.156	10	Ctrl vs. Mosaic p = 0.964 (n.s.)
Mosaic	79.36	9.624	9	
**Decay time (ms)**	**Mean**	**SEM**	**N**	**p value**<br>(Two – tailed unpaired Student's t – test)
Ctrl	12.59	0.624	10	Ctrl vs. Mosaic p = 0.191 (n.s.)
Mosaic	11.42	0.580	9	
**Frequency (Hz)**	**Mean**	**SEM**	**N**	**p value**<br>(Two – tailed unpaired Student's t – test)
Ctrl	0.372	0.043	10	Ctrl vs. Mosaic p = 0.133 (n.s.)
Mosaic	0.290	0.027	9	

**Fig. 3h**<br><br>**Amplitude (pA)**	**Mean**	**SEM**	**N**	**p value**<br>(Two – tailed unpaired Student's t – test)
Ctrl	9.554	0.786	13	Ctrl vs. Mosaic p = 0.417 (n.s.)
Mosaic	10.57	0.930	9	
**Area (pA * ms)**	**Mean**	**SEM**	**N**	**p value**<br>(Two – tailed unpaired Student's t – test)
Ctrl	145.0	25.26	13	Ctrl vs. Mosaic p = 0.897 (n.s.)
Mosaic	150.3	32.58	9	
**Decay time (ms)**	**Mean**	**SEM**	**N**	**p value**<br>(Two – tailed unpaired Student's t – test)
Ctrl	28.82	1.952	13	Ctrl vs. Mosaic p = 0.595 (n.s.)
Mosaic	31.64	5.682	9	
**Frequency (Hz)**	**Mean**	**SEM**	**N**	**p value**<br>(Two – tailed unpaired Student's t – test)
Ctrl	0.500	0.933	13	Ctrl vs. Mosaic p = 0.004 (**)
Mosaic	0.073	0.117	9	

Supplementary table 4. Mean values, SEM, number of samples and p values of data displayed in Figure 4.

**Fig. 4a**<br><br>**MFR (spike / s)**	**Mean**	**SEM**	**N**	**p value**<br>(Two – tailed unpaired Student's t – test)
Ctrl	0.734	0.097	6	Ctrl vs. Mosaic p = 0.029 (*)
Mosaic	0.485	0.013	6	
**MBR (burst / min)**	**Mean**	**SEM**	**N**	**p value**<br>(Two – tailed unpaired Student's t – test)
Ctrl	3.40	0.383	6	Ctrl vs. Mosaic p = 0.131 (n.s.)
Mosaic	2.76	0.077	6	
**MBD (ms)**	**Mean**	**SEM**	**N**	**p value**<br>(Two – tailed unpaired Student's t – test)
Ctrl	83.81	9.947	6	Ctrl vs. Mosaic p = 0.032 (*)
Mosaic	55.95	5.115	6	
**SI (a.u.)**	**Mean**	**SEM**	**N**	**p value**<br>(Two – tailed unpaired Student's t – test)
Ctrl	0.243	0.007	6	Ctrl vs. Mosaic p = 0.092 (n.s.)
Mosaic	0.313	0.037	6	

**Fig. 4b**<br><br>**MFR (spike / s)**	**Mean**	**SEM**	**N**	**p value**<br>(Two – tailed unpaired Student's t – test)
Scrambled	5.934	1.561	8	Scrambled vs. *PCDH19* shRNA p = 0.039 (*)
PCDH19 shRNA	2.051	0.682	8	
**MBR (burst / min)**	**Mean**	**SEM**	**N**	**p value**<br>(Two – tailed unpaired Student's t – test)
Scrambled	6.655	0.865	8	Scrambled vs. *PCDH19* shRNA p = 0.047 (*)
PCDH19 shRNA	4.179	0.683	7	
**MBD (ms)**	**Mean**	**SEM**	**N**	**p value**<br>(Two – tailed unpaired Student's t – test)
Scrambled	522.4	82.59	8	Scrambled vs. *PCDH19* shRNA p = 0.033 (*)
PCDH19 shRNA	291.8	41.85	7	

**Fig. 4c**<br><br>**MFR (spike / s)**	**Mean**	**SEM**	**N**	**p value**<br>(Two – tailed unpaired Student's t – test)
Ctrl	0.866	0.028	14	Ctrl vs. Mosaic p < 0.001 (***)
Mosaic	0.632	0.051	8	

MBR (burst / min)	Mean	SEM	N	p value (Two – tailed unpaired Student's t – test)
Ctrl	2.559	0.164	14	Ctrl vs. Mosaic p < 0.001 (***)
Mosaic	1.598	0.130	8	
**MBD (ms)**	**Mean**	**SEM**	**N**	**p value** (Mann – Whitney test)
Ctrl	81.82	4.731	14	Ctrl vs. Mosaic p = 0.095 (n.s.)
Mosaic	100.4	13.75	8	
**PSB (%)**	**Mean**	**SEM**	**N**	**p value** (Two – tailed unpaired Student's t – test)
Ctrl	12.18	1.481	14	Ctrl vs. Mosaic p = 0.081 (n.s.)
Mosaic	28.87	7.304	8	
**SI (a.u.)**	**Mean**	**SEM**	**N**	**p value** (Mann – Whitney test)
Ctrl	0.102	0.008	14	Ctrl vs. Mosaic p < 0.001 (***)
Mosaic	0.178	0.019	8	

**Fig. 4c- *inset*** **MFR-CA1 (spike / s)**	**Mean**	**SEM**	**N**	**p value** (Two – tailed unpaired Student's t – test)
Ctrl	0.313	0.032	14	Ctrl vs. Mosaic p < 0.001 (***)
Mosaic	0.087	0.025	7	
**MFR-CA3 (spike / s)**	**Mean**	**SEM**	**N**	**p value** (Two – tailed unpaired Student's t – test)
Ctrl	0.328	0.040	14	Ctrl vs. Mosaic p < 0.001 (***)
Mosaic	0.080	0.013	7	
**MFR-DG (spike / s)**	**Mean**	**SEM**	**N**	**p value** (Two – tailed unpaired Student's t – test)
Ctrl	0.307	0.034	14	Ctrl vs. Mosaic p < 0.001 (***)
Mosaic	0.098	0.017	7	

Supplementary table 5. Mean values, SEM, number of samples and p values of data displayed in Figure 5.

**Fig. 5b** **MFR (spike / s)**	**Mean**	**SEM**	**N**	**p value** (Two – tailed unpaired Student's t – test)
Ctrl	55.50	9.84	20	Ctrl vs. Mosaic p = 0.001 (**)
Mosaic	32.44	4.88	28	
**MFR-CA1**	**Mean**	**SEM**	**N**	**p value**

(spike / s)				(Two – tailed unpaired Student's t – test)
Ctrl	24.53	0.60	5	Ctrl vs. Mosaic p < 0.05 (*)
Mosaic	18.91	0.33	7	
**MFR-Amy (spike / s)**	**Mean**	**SEM**	**N**	**p value** (Two – tailed unpaired Student's t – test)
Ctrl	91.87	1.37	5	Ctrl vs. Mosaic p < 0.001 (***)
Mosaic	50.69	0.36	7	
**MFR-ERC (spike / s)**	**Mean**	**SEM**	**N**	**p value** (Two – tailed unpaired Student's t – test)
Ctrl	87.09	2.70	5	Ctrl vs. Mosaic p < 0.001 (***)
Mosaic	47.64	1.15	7	
**MFR-PRC (spike / s)**	**Mean**	**SEM**	**N**	**p value** (Two – tailed unpaired Student's t – test)
Ctrl	18.51	0.99	5	Ctrl vs. Mosaic p < 0.05 (*)
Mosaic	12.51	0.38	7	

**Fig. 5d** **Excitatory neurons**				
**Neurons / recording session (n) -CA1**	**Mean**	**SEM**	**N**	**p value** (Two – tailed unpaired Student's t – test)
Ctrl	25.00	0.53	5	Ctrl vs. Mosaic p = 0.96 (n.s.)
Mosaic	23.5	0.40	7	
**Neurons / recording session (n) -Amy**	**Mean**	**SEM**	**N**	**p value** (Two – tailed unpaired Student's t – test)
Ctrl	24.83	0.68	5	Ctrl vs. Mosaic p = 0.94 (n.s.)
Mosaic	22.67	0.36	7	
**Neurons / recording session (n) -ERC**	**Mean**	**SEM**	**N**	**p value** (Two – tailed unpaired Student's t – test)
Ctrl	26.50	0.31	5	Ctrl vs. Mosaic p = 0.54 (n.s.)
Mosaic	21.50	0.48	7	
**Neurons / recording session (n) -PRC**	**Mean**	**SEM**	**N**	**p value** (Two – tailed unpaired Student's t – test)
Ctrl	23.50	0.67	5	Ctrl vs. Mosaic p = 0.70 (n.s.)
Mosaic	21.67	0.54	7	
**Inhibitory neurons**				
	**Mean**	**SEM**	**N**	**p value**

Neurons / recording session (n) -CA1				(Two – tailed unpaired Student's t – test)
Ctrl	11.00	0.38	5	Ctrl vs. Mosaic p = 0.12 (n.s.)
Mosaic	15.83	0.24	7	
**Neurons / recording session (n) -Amy**	**Mean**	**SEM**	**N**	**p value** (Two – tailed unpaired Student's t – test)
Ctrl	10.67	0.62	5	Ctrl vs. Mosaic p = 0.45 (n.s.)
Mosaic	12.16	0.59	7	
**Neurons / recording session (n) -ERC**	**Mean**	**SEM**	**N**	**p value** (Two – tailed unpaired Student's t – test)
Ctrl	9.67	0.57	5	Ctrl vs. Mosaic p = 0.90 (n.s.)
Mosaic	11.00	0.63	7	
**Neurons / recording session (n) -PRC**	**Mean**	**SEM**	**N**	**p value** (Two – tailed unpaired Student's t – test)
Ctrl	10.50	0.79	5	Ctrl vs. Mosaic p = 0.58 (n.s.)
Mosaic	12.16	0.38	7	

E / I ratio

**Neurons-CA1 (E / I ratio)**	**Mean**	**SEM**	**N**	**p value** (Two – tailed unpaired Student's t – test)
Ctrl	2.42	0.15	5	Ctrl vs. Mosaic p = 0.11 (n.s.)
Mosaic	1.51	0.04	7	
**Neurons-Amy (E / I ratio)**	**Mean**	**SEM**	**N**	**p value** (Two – tailed unpaired Student's t – test)
Ctrl	2.60	0.20	5	Ctrl vs. Mosaic p = 0.33 (n.s.)
Mosaic	1.94	0.06	7	
**Neurons-ERC (E / I ratio)**	**Mean**	**SEM**	**N**	**p value** (Two – tailed unpaired Student's t – test)
Ctrl	2.96	0.13	5	Ctrl vs. Mosaic p = 0.27 (n.s.)
Mosaic	2.20	0.16	7	
**Neurons-PRC (E / I ratio)**	**Mean**	**SEM**	**N**	**p value** (Two – tailed unpaired Student's t – test)
Ctrl	2.68	0.22	5	Ctrl vs. Mosaic p = 0.19 (n.s.)
Mosaic	1.86	0.10	7	

Fig. 5e

Excitatory neurons

**MFR-CA1 (spike / s)**	**Mean**	**SEM**	**N**	**p value** (Two – tailed unpaired Student's t – test)
Ctrl	12.16	0.22	5	Ctrl vs. Mosaic p = 0.03 (*)
Mosaic	6.79	047	7	
**MFR-Amy (spike / s)**	**Mean**	**SEM**	**N**	**p value** (Two – tailed unpaired Student's t – test)
Ctrl	42.21	0.59	5	Ctrl vs. Mosaic p < 0.001 (***)
Mosaic	32.02	0.53	7	
**MFR-ERC (spike / s)**	**Mean**	**SEM**	**N**	**p value** (Two – tailed unpaired Student's t – test)
Ctrl	40.41	0.49	5	Ctrl vs. Mosaic p < 0.001 (***)
Mosaic	29.36	0.35	7	
**MFR-PRC (spike / s)**	**Mean**	**SEM**	**N**	**p value** (Two – tailed unpaired Student's t – test)
Ctrl	15.14	0.46	5	Ctrl vs. Mosaic p = 0.01 (*)
Mosaic	10.14	0.32	7	
**Inhibitory neurons**				
**Neurons-CA1 (spike / s)**	**Mean**	**SEM**	**N**	**p value** (Two – tailed unpaired Student's t – test)
Ctrl	33.34	0.37	5	Ctrl vs. Mosaic p = 0.02 (*)
Mosaic	49.35	1.34	7	
**Neurons-Amy (spike / s)**	**Mean**	**SEM**	**N**	**p value** (Two – tailed unpaired Student's t – test)
Ctrl	66.57	0.90	5	Ctrl vs. Mosaic p = 0.03 (*)
Mosaic	81.59	1.64	7	
**Neurons-ERC (spike / s)**	**Mean**	**SEM**	**N**	**p value** (Two – tailed unpaired Student's t – test)
Ctrl	68.09	0.14	5	Ctrl vs. Mosaic p = 0.003 (**)
Mosaic	86.34	1.87	7	
**Neurons-PRC (spike / s)**	**Mean**	**SEM**	**N**	**p value** (Two – tailed unpaired Student's t – test)
Ctrl	27.10	0.38	5	Ctrl vs. Mosaic p = 0.92 (n.s)
Mosaic	32.31	1.81	7	
**E / I ratio**				
**Neurons-CA1**	**Mean**	**SEM**	**N**	**p value**

(spike / s)				(Two – tailed unpaired Student's t – test)
Ctrl	0.36	0.007	5	Ctrl vs. Mosaic p < 0.001 (***)
Mosaic	0.14	0.010	7	
**Neurons-Amy (spike / s)**	**Mean**	**SEM**	**N**	**p value** (Two – tailed unpaired Student's t – test)
Ctrl	0.64	0.012	5	Ctrl vs. Mosaic p < 0.001 (***)
Mosaic	0.39	0.009	7	
**Neurons-ERC (spike / s)**	**Mean**	**SEM**	**N**	**p value** (Two – tailed unpaired Student's t – test)
Ctrl	0.59	0.007	5	Ctrl vs. Mosaic p < 0.001 (***)
Mosaic	0.34	0.004	7	
**Neurons-PRC (spike / s)**	**Mean**	**SEM**	**N**	**p value** (Two – tailed unpaired Student's t – test)
Ctrl	0.56	0.021	5	Ctrl vs. Mosaic p < 0.001 (***)
Mosaic	0.33	0.013	7	

Supplementary table 6. Mean values, SEM, number of samples and p values of data displayed in Figure 6.

**Fig. 6b** **LFP SI (a.u.)**	**Mean**	**SEM**	**N**	**p value** (Two – tailed unpaired Student's t – test)
Ctrl	0.485	0.006	20	Ctrl vs. Mosaic p < 0.001 (***)
Mosaic	0.552	0.005	28	
**LFP SI-CA1 (a.u.)**	**Mean**	**SEM**	**N**	**p value** (Two – tailed unpaired Student's t – test)
Ctrl	0.500	0.011	5	Ctrl vs. Mosaic p = 0.03 (*)
Mosaic	0.567	0.006	7	
**LFP SI-Amy (a.u.)**	**Mean**	**SEM**	**N**	**p value** (Two – tailed unpaired Student's t – test)
Ctrl	0.461	0.015	5	Ctrl vs. Mosaic p < 0.001 (***)
Mosaic	0.540	0.014	7	
**LFP SI-ERC (a.u.)**	**Mean**	**SEM**	**N**	**p value** (Two – tailed unpaired Student's t – test)
Ctrl	0.496	0.009	5	Ctrl vs. Mosaic p = 0.02 (*)
Mosaic	0.551	0.006	7	
**LFP SI-PRC (a.u.)**	**Mean**	**SEM**	**N**	**p value** (Two – tailed unpaired Student's t – test)

Ctrl	0.486	0.010	5	Ctrl vs. Mosaic p < 0.001 (***)
Mosaic	0.562	0.012	7	

Fig. 6c

α-bands

**Power spectral density (dB / Hz) CA1**	**Mean**	**SEM**	**N**	**p value** (Two – tailed unpaired Student's t – test)
Ctrl	0.37	0.02	5	Ctrl vs. Mosaic p = 0.344 (n.s.)
Mosaic	0.29	0.02	7	
**Power spectral density (dB / Hz) Amy**	**Mean**	**SEM**	**N**	**p value** (Two – tailed unpaired Student's t – test)
Ctrl	3.11	0.56	5	Ctrl vs. Mosaic p = 0.062 (n.s.)
Mosaic	3.67	0.49	7	
**Power spectral density (dB / Hz) ERC**	**Mean**	**SEM**	**N**	**p value** (Two – tailed unpaired Student's t – test)
Ctrl	0.72	0.05	5	Ctrl vs. Mosaic p = 0.175 (n.s.)
Mosaic	1.28	0.16	7	
**Power spectral density (dB / Hz) PRC**	**Mean**	**SEM**	**N**	**p value** (Two – tailed unpaired Student's t – test)
Ctrl	0.24	0.01	5	Ctrl vs. Mosaic p = 0.029 (*)
Mosaic	0.35	0.03	7	

β-bands

**Power spectral density (dB / Hz) CA1**	**Mean**	**SEM**	**N**	**p value** (Two – tailed unpaired Student's t – test)
Ctrl	0.03	0.00	5	Ctrl vs. Mosaic p = 0.118 (n.s.)
Mosaic	0.05	0.00	7	
**Power spectral density (dB / Hz) Amy**	**Mean**	**SEM**	**N**	**p value** (Two – tailed unpaired Student's t – test)

Ctrl	0.31	0.05	5	Ctrl vs. Mosaic $p = 0.071$ (n.s.)
Mosaic	0.39	0.04	7	
**Power spectral density (dB / Hz) ERC**	**Mean**	**SEM**	**N**	**p value** (Two – tailed unpaired Student's t – test)
Ctrl	0.09	0.01	5	Ctrl vs. Mosaic $p < 0.001$ (***)
Mosaic	0.13	0.01	7	
**Power spectral density (dB / Hz) PRC**	**Mean**	**SEM**	**N**	**p value** (Two – tailed unpaired Student's t – test)
Ctrl	0.02	0.00	5	Ctrl vs. Mosaic $p = 0.006$ (**)
Mosaic	0.04	0.00	7	
**γ-bands**				
**Power spectral density (dB / Hz) CA1**	**Mean**	**SEM**	**N**	**p value** (Two – tailed unpaired Student's t – test)
Ctrl	0.002	0.00	5	Ctrl vs. Mosaic $p = 0.002$ (**)
Mosaic	0.005	0.00	7	
**Power spectral density (dB / Hz) Amy**	**Mean**	**SEM**	**N**	**p value** (Two – tailed unpaired Student's t – test)
Ctrl	0.02	0.00	5	Ctrl vs. Mosaic $p = 0.053$
Mosaic	0.04	0.00	7	
**Power spectral density (dB / Hz) ERC**	**Mean**	**SEM**	**N**	**p value** (Two – tailed unpaired Student's t – test)
Ctrl	0.00	0.00	5	Ctrl vs. Mosaic $p < 0.001$ (***)
Mosaic	0.02	0.00	7	
**Power spectral density (dB / Hz) PRC**	**Mean**	**SEM**	**N**	**p value** (Two – tailed unpaired Student's t – test)
Ctrl	0.001	0.00	5	Ctrl vs. Mosaic $p < 0.001$ (***)
Mosaic	0.003	0.00	7	

Supplementary table 7. Mean values, SEM, number of samples and p values of data displayed in Supplementary Figure 1.

Suppl. Fig. 1a<br>Fold change	Mean	SEM	N	p value<br>(Two – tailed unpaired Student's t – test)
Ctrl	1.000	0.000	3	Ctrl vs E 1 – 2 p < 0.001 (***)<br>Ctrl vs E 4 – 5 p = 0.007 (**)<br>Ctrl vs E 5 – 6 p < 0.001 (***)
E 1 – 2	0.013	0.001	3	
E 4 – 5	0.170	0.036	3	
E 5 – 6	0.092	0.006	3	

Supplementary table 8. Mean values, SEM and number of samples of data displayed in Supplementary Figure 2.

Suppl. Fig. 2b<br>PCDH19 – PSD95 colocalization	Mean	SEM	N
R	0.757	0.009	13
M1	0.444	0.033	13
M2	0.432	0.025	13
**PCDH19 – VGLUT colocalization**	**Mean**	**SEM**	**N**
R	0.907	0.009	13
M1	0.726	0.021	13
M2	0.749	0.016	13

Suppl. Fig. 2c<br>PCDH19 – beta3 colocalization	Mean	SEM	N
R	0.684	0.017	15
M1	0.280	0.017	15
M2	0.381	0.020	15
**PCDH19 – VGAT colocalization**	**Mean**	**SEM**	**N**
R	0.748	0.026	15
M1	0.529	0.039	15
M2	0.666	0.030	15

Supplementary table 9. Mean values, SEM, number of samples and p values of data displayed in Supplementary Figure 3.

Suppl. Fig. 3c<br>PCDH19 / GAPDH	Mean	SEM	N	p value

				(One – way ANOVA)
WT	1.787	0.439	5	p > 0.05 (n.s.)
Pcdh19 $^{fl/x}$	1.082	0.223	5	
Pcdh19 $^{fl/fl}$	2.245	0.689	5	

Supplementary table 10. Mean values, SEM, number of samples and p values of data displayed in Supplementary Figure 4.

**Suppl. Fig. 4a** **PCDH19 / tubulin**	**Mean**	**SEM**	**N**	**p value** (Two – tailed unpaired Student's t – test)
Ctrl	0.971	0.067	3	Ctrl vs Mosaic p = 0.019 (*)
Mosaic	0.548	0.088	3	

**Suppl. Fig. 4c** **Synaptic density (N / µm³)**	**Mean**	**SEM**	**N**	**p value** (Two – tailed unpaired Student's t – test)
PCDH19 +	2.227	0.207	18	PCDH19 + vs. PCDH19 - p = 0.015 (*)
PCDH19 -	1.494	0.112	16	
**PSD thickness (nm)**	**Mean**	**SEM**	**N**	**p value** (Mann – Whitney test)
PCDH19 +	47.44	1.048	113	PCDH19 + vs. PCDH19 - p = 0.017 (*)
PCDH19 -	43.58	1.011	106	
**PSD length (nm)**	**Mean**	**SEM**	**N**	**p value** (Mann – Whitney test)
PCDH19 +	259.8	7.199	114	PCDH19 + vs. PCDH19 - p = 0.224 (n.s.)
PCDH19 -	265.5	6.942	108	
**SAL (nm)**	**Mean**	**SEM**	**N**	**p value** (Mann – Whitney test)
PCDH19 +	328.7	11.79	114	PCDH19 + vs. PCDH19 - p = 0.413 (n.s.)
PCDH19 -	313.9	11.02	108	
**Spine head area (µm²)**	**Mean**	**SEM**	**N**	**p value** (Mann – Whitney test)
PCDH19 +	0.118	0.006	99	PCDH19 + vs. PCDH19 - p = 0.042 (*)
PCDH19 -	0.099	0.005	98	
**Presynaptic area (µm²)**	**Mean**	**SEM**	**N**	**p value** (Mann – Whitney test)
PCDH19 +	0.187	0.010	97	PCDH19 + vs. PCDH19 - p = 0.422 (n.s.)
PCDH19 -	0.177	0.011	80	
**Vesicle density**	**Mean**	**SEM**	**N**	**p value**

(N / µm²)				(Mann – Whitney test)
PCDH19 +	335.8	12.83	101	PCDH19 + vs. PCDH19 - p $<$ 0.001 (***)
PCDH19 -	232.5	8.509	94	

**Suppl. Fig. 4d**<br>**Distribution of PSD thickness (nm)**	**PCDH19 +, %**	**PCDH19 -, %**	**p value**<br>(Comparison of Z-score, Mann-Whitney test)
10	0.00	0.00	PCDH19 + vs. PCDH19 –<br>p = 0.974 (ns)
20	0.88	1.85	
30	8.77	21.30	
40	32.46	28.70	
50	35.96	34.26	
60	14.04	10.19	
70	4.39	0.93	
80	2.63	0.93	
90	0.00	0.00	
100	0.00	0.93	
110	0.00	0.00	
120	0.88	0.00	
130	0.00	0.00	
140	0.00	0.00	
150	0.00	0.00	
160	0.00	0.00	
170	0.00	0.93	

**Distribution of vesicle density (N / µm²)**	**PCDH19 +, %**	**PCDH19 -, %**	**p value**<br>(Comparison of Z-score, Mann-Whitney test)
50	0	0	PCDH19 + vs. PCDH19 –<br>p = 0.783 (ns)
100	0	7.45	
150	0	13.83	
200	13.86	31.91	
250	20.79	25.53	
300	18.82	11.70	
350	20.79	3.19	
400	8.91	2.13	
450	5.94	3.19	
500	4.95	1.064	
550	2.97	0	
600	0.99	0	
650	0	0	
700	0	0	
750	0	0	
800	0.99	0	
850	0	0	
900	0	0	
950	0	0	

1000	0	0	
1050	0.99	0	
1100	0	0	

Supplementary table 11. Mean values, SEM, number of samples and p values of data displayed in Supplementary Figure 5.

**Suppl. Fig. 5a**<br><br>**MFR (spike / s)**	**Mean**	**SEM**	**N**	**p value**<br>(Two – tailed unpaired Student's t – test)
Ctrl	0.615	0.055	8	Ctrl vs. Mosaic p = 0.036 (*)
Mosaic	0.448	0.035	6	
**MBR (burst / min)**	**Mean**	**SEM**	**N**	**p value**<br>(Two – tailed unpaired Student's t – test)
Ctrl	1.889	0.131	8	Ctrl vs. Mosaic p < 0.001 (***)
Mosaic	0.912	0.141	6	
**MBD (ms)**	**Mean**	**SEM**	**N**	**p value**<br>(Two – tailed unpaired Student's t – test)
Ctrl	78.52	7.386	8	Ctrl vs. Mosaic p = 0.282 (n.s.)
Mosaic	93.55	0.141	6	
**PSB (%)**	**Mean**	**SEM**	**N**	**p value**<br>(Two – tailed unpaired Student's t – test)
Ctrl	19.33	2.874	8	Ctrl vs. Mosaic p = 0.455 (n.s.)
Mosaic	14.76	1.413	6	
**SI (a.u.)**	**Mean**	**SEM**	**N**	**p value**<br>(Two – tailed unpaired Student's t – test)
Ctrl	0.164	0.008	8	Ctrl vs. Mosaic p = 0.011 (*)
Mosaic	0.220	0.018	6	

**Suppl. Fig. 5a-*inset***<br><br>**MFR-CA1 (spike / s)**	**Mean**	**SEM**	**N**	**p value**<br>(Two – tailed unpaired Student's t – test)
Ctrl	0.101	0.019	7	Ctrl vs. Mosaic p = 0.059 (n.s.)
Mosaic	0.048	0.013	6	
**MFR-CA3 (spike / s)**	**Mean**	**SEM**	**N**	**p value**<br>(Two – tailed unpaired Student's t – test)
Ctrl	0.086	0.015	8	Ctrl vs. Mosaic p = 0.276 (n.s.)
Mosaic	0.060	0.016	6	
**MFR-DG (spike / s)**	**Mean**	**SEM**	**N**	**p value**<br>(Two – tailed unpaired Student's t – test)

Ctrl	0.081	0.009	8	Ctrl vs. Mosaic p = 0.047 (*)
Mosaic	0.050	0.010	6	

**Suppl. Fig.5b**<br><br>**MFR (spike / s)**	**Mean**	**SEM**	**N**	**p value**<br>(Two – tailed unpaired Student's t – test)
Ctrl	0.803	0.057	5	Ctrl vs. Mosaic p = 0.015 (*)
Mosaic	0.448	0.035	5	
**MBR (burst / min)**	**Mean**	**SEM**	**N**	**p value**<br>(Two – tailed unpaired Student's t – test)
Ctrl	1.699	0.166	5	Ctrl vs. Mosaic p = 0.047 (*)
Mosaic	1.020	0.238	5	
**MBD (ms)**	**Mean**	**SEM**	**N**	**p value**<br>(Two – tailed unpaired Student's t – test)
Ctrl	77.80	3.334	5	Ctrl vs. Mosaic p = 0.976 (n.s.)
Mosaic	78.41	20.07	5	
**PSB (%)**	**Mean**	**SEM**	**N**	**p value**<br>(Two – tailed unpaired Student's t – test)
Ctrl	11.26	1.690	5	Ctrl vs. Mosaic p = 0.336 (n.s.)
Mosaic	16.01	4.334	5	
**SI (a.u.)**	**Mean**	**SEM**	**N**	**p value**<br>(Two – tailed unpaired Student's t – test)
Ctrl	0.101	0.009	5	Ctrl vs. Mosaic p = 0.001 (**)
Mosaic	0.153	0.005	5	

**Suppl. Fig. 5b-*inset***<br><br>**MFR-CA1 (spike / s)**	**Mean**	**SEM**	**N**	**p value**<br>(Two – tailed unpaired Student's t – test)
Ctrl	0.244	0.056	5	Ctrl vs. Mosaic p = 0.046 (*)
Mosaic	0.100	0.023	5	
**MFR-CA3 (spike / s)**	**Mean**	**SEM**	**N**	**p value**<br>(Two – tailed unpaired Student's t – test)
Ctrl	0.284	0.047	5	Ctrl vs. Mosaic p = 0.002 (**)
Mosaic	0.078	0.009	5	
**MFR-DG (spike / s)**	**Mean**	**SEM**	**N**	**p value**<br>(Two – tailed unpaired Student's t – test)
Ctrl	0.320	0.102	5	Ctrl vs. Mosaic p = 0.049 (*)
Mosaic	0.083	0.012	5	

Supplementary table 12. Mean values, SEM, number of samples and p values of data displayed in Supplementary Figure 7.

**Suppl. Fig. 7a**<br><br>**PCDH19 mRNA expression**	**Mean**	**SEM**	**N**	**p value**<br>(Two – tailed unpaired Student's t – test)
Ctrl (Cortex)	10.16	1.151	4	Ctrl vs. Mosaic p = 0.019 (*)
Mosaic (Cortex)	3.24	1.802	3	
Ctrl (Hippocampus)	11.74	0.735	5	Ctrl vs. Mosaic p = 0.001 (**)
Mosaic (Hippocampus)	4.339	1.229	3	

**Suppl. Fig. 7b**<br><br>**PCDH19 / GAPDH**	**Mean**	**SEM**	**N**	**p value**<br>(Two – tailed unpaired Student's t – test)
Ctrl (Cortex)	0.999	0.097	8	Ctrl vs. Mosaic p < 0.001 (***)
Mosaic (Cortex)	0.393	0.069	9	
Ctrl (Hippocampus)	1.000	0.067	6	Ctrl vs. Mosaic p < 0.001 (***)
Mosaic (Hippocampus)	0.393	0.041	7	

**Suppl. Fig. 7d**<br><br>**PCDH19 / GAPDH**	**Mean**	**SEM**	**N**	**p value**<br>(One – way ANOVA)
WT	1.896	0.508	7	p > 0.05 (n.s.)
Pcdh19 $^{fl/y}$	1.442	0.209	6	
Syn1-Cre	2.343	0.710	6	

Supplementary table 13. Mean values, SEM, number of samples and p values of data displayed in Supplementary Figure 8.

**Suppl. Fig. 8**				**δ-bands**
**Power spectral density (dB / Hz) CA1**	**Mean**	**SEM**	**N**	**p value**<br>(Two – tailed unpaired Student's t – test)
Ctrl	1.69	0.14	5	Ctrl vs. Mosaic p = 0.999 (n.s.)
Mosaic	1.56	0.15	7	
**Power spectral density (dB / Hz) Amy**	**Mean**	**SEM**	**N**	**p value**<br>(Two – tailed unpaired Student's t – test)
Ctrl	14.88	3.22	5	Ctrl vs. Mosaic p = 0.999 (n.s.)
Mosaic	14.44	2.93	7	

Power spectral density (dB / Hz) ERC	Mean	SEM	N	p value (Two – tailed unpaired Student's t – test)
Ctrl	3.33	0.61	5	Ctrl vs. Mosaic p = 0.990 (n.s.)
Mosaic	2.09	0.53	7	
**Power spectral density (dB / Hz) PRC**	**Mean**	**SEM**	**N**	**p value** (Two – tailed unpaired Student's t – test)
Ctrl	1.85	0.09	5	Ctrl vs. Mosaic p = 0.999 (n.s.)
Mosaic	1.20	0.07	7	
**θ-bands**				
**Power spectral density (dB / Hz) CA1**	**Mean**	**SEM**	**N**	**p value** (Two – tailed unpaired Student's t – test)
Ctrl	0.88	0.07	5	Ctrl vs. Mosaic p = 0.999 (n.s.)
Mosaic	0.74	0.06	7	
**Power spectral density (dB / Hz) Amy**	**Mean**	**SEM**	**N**	**p value** (Two – tailed unpaired Student's t – test)
Ctrl	7.62	1.38	5	Ctrl vs. Mosaic p = 0.999 (n.s.)
Mosaic	7.53	1.45	7	
**Power spectral density (dB / Hz) ERC**	**Mean**	**SEM**	**N**	**p value** (Two – tailed unpaired Student's t – test)
Ctrl	1.85	0.20	5	Ctrl vs. Mosaic p = 0.981 (n.s.)
Mosaic	0.80	1.60	7	
**Power spectral density (dB / Hz) PRC**	**Mean**	**SEM**	**N**	**p value** (Two – tailed unpaired Student's t – test)
Ctrl	0.63	0.03	5	Ctrl vs. Mosaic p = 0.993 (n.s.)
Mosaic	0.41	0.02	7	

Supplementary table 14. Mean values, SEM, number of samples and p values of data displayed in Supplementary Figure 9.

Suppl. Fig. 9b<br><br>LFP rate<br>(LFP / s)	Mean	SEM	N	p value<br>(Two – tailed unpaired Student's t – test)
Ctrl	0.253	0.020	12	Ctrl vs. Mosaic p = 0.305 (n.s.)
Mosaic	0.290	0.025	6	
**LFP amplitude (μV)**	**Mean**	**SEM**	**N**	**p value**<br>(Two – tailed unpaired Student's t – test)
Ctrl	111.2	6.132	12	Ctrl vs. Mosaic p = 0.005 (**)
Mosaic	83.05	1.813	6	
**LFP duration (s)**	**Mean**	**SEM**	**N**	**p value**<br>(Two – tailed unpaired Student's t – test)
Ctrl	0.091	0.013	12	Ctrl vs. Mosaic p = 0.021 (*)
Mosaic	0.042	0.001	6	
**LFP syncro (a.u.)**	**Mean**	**SEM**	**N**	**p value**<br>(Two – tailed unpaired Student's t – test)
Ctrl	0.552	0.035	12	Ctrl vs. Mosaic p = 0.462 (n.s.)
Mosaic	0.594	0.035	6	

Suppl. Fig. 9c<br><br>LFP rate<br>(LFP / s)	Mean	SEM	N	p value<br>(Two – tailed unpaired Student's t – test)
Ctrl	0.343	0.026	13	Ctrl vs. Mosaic p = 0.061 (n.s.)
Mosaic	0.250	0.030	8	
**LFP amplitude (μV)**	**Mean**	**SEM**	**N**	**p value**<br>(Two – tailed unpaired Student's t – test)
Ctrl	90.06	3.183	13	Ctrl vs. Mosaic p = 0.120 (n.s.)
Mosaic	101.9	7.775	8	
**LFP duration (s)**	**Mean**	**SEM**	**N**	**p value**<br>(Two – tailed unpaired Student's t – test)
Ctrl	0.056	0.008	13	Ctrl vs. Mosaic p = 0.045 (*)
Mosaic	0.101	0.023	8	
**LFP syncro (a.u.)**	**Mean**	**SEM**	**N**	**p value**<br>(Two – tailed unpaired Student's t – test)
Ctrl	0.580	0.008	13	Ctrl vs. Mosaic p = 0.901 (n.s.)
Mosaic	0.554	0.029	8	

Suppl. Fig. 9d<br><br>LFP rate	Mean	SEM	N	p value

(LFP / s)				(Two – tailed unpaired Student's t – test)
Ctrl	0.344	0.042	5	Ctrl vs. Mosaic p = 0.753 (n.s.)
Mosaic	0.326	0.034	5	
**LFP amplitude (μV)**	**Mean**	**SEM**	**N**	**p value** (Two – tailed unpaired Student's t – test)
Ctrl	117.8	15.25	5	Ctrl vs. Mosaic p = 0.100 (n.s.)
Mosaic	88.29	4.436	5	
**LFP duration (s)**	**Mean**	**SEM**	**N**	**p value** (Two – tailed unpaired Student's t – test)
Ctrl	0.029	0.005	5	Ctrl vs. Mosaic p = 0.079 (n.s.)
Mosaic	0.070	0.019	5	
**LFP syncro (a.u.)**	**Mean**	**SEM**	**N**	**p value** (Two – tailed unpaired Student's t – test)
Ctrl	0.556	0.008	5	Ctrl vs. Mosaic p = 0.232 (n.s.)
Mosaic	0.528	0.020	5	

**Suppl. Fig. 9e** **Power spectral density (dB / Hz) α-bands**	**Mean**	**SEM**	**N**	**p value** (Two – tailed unpaired Student's t – test)
Ctrl	23.60	2.12	12	Ctrl vs. Mosaic p < 0.05 (*)
Mosaic	33.67	1.20	6	
**Power spectral density (dB / Hz) β-bands**	**Mean**	**SEM**	**N**	**p value** (Two – tailed unpaired Student's t – test)
Ctrl	-1	2.08	12	Ctrl vs. Mosaic p < 0.001 (***)
Mosaic	21	0.57	6	
**Power spectral density (dB / Hz) γ-bands**	**Mean**	**SEM**	**N**	**p value** (Two – tailed unpaired Student's t – test)
Ctrl	- 4.73	0.63	12	Ctrl vs. Mosaic p < 0.001 (***)
Mosaic	11	1.15	6	

**Suppl. Fig. 9f** **Power spectral density (dB / Hz) α-bands**	**Mean**	**SEM**	**N**	**p value** (Two – tailed unpaired Student's t – test)

Ctrl	21.67	3.71	13	Ctrl vs. Mosaic p > 0.05 (n.s.)
Mosaic	28	3.05	8	
**Power spectral density (dB / Hz) β-bands**	**Mean**	**SEM**	**N**	**p value** (Two – tailed unpaired Student's t – test)
Ctrl	2.433	0.296	13	Ctrl vs. Mosaic p < 0.01 (**)
Mosaic	9.20	0.986	8	
**Power spectral density (dB / Hz) γ-bands**	**Mean**	**SEM**	**N**	**p value** (Two – tailed unpaired Student's t – test)
Ctrl	0.167	0.731	13	Ctrl vs. Mosaic p > 0.05 (n.s.)
Mosaic	0.30	0.87	8	

**Suppl. Fig. 9g** **Power spectral density (dB / Hz) α-bands**	**Mean**	**SEM**	**N**	**p value** (Two – tailed unpaired Student's t – test)
Ctrl	25.91	4.76	13	Ctrl vs. Mosaic p = 0.998 (n.s.)
Mosaic	25.13	5.11	8	
**Power spectral density (dB / Hz) β-bands**	**Mean**	**SEM**	**N**	**p value** (Two – tailed unpaired Student's t – test)
Ctrl	5.45	2.02	13	Ctrl vs. Mosaic p < 0.001 (***)
Mosaic	-3.84	1.32	8	
**Power spectral density (dB / Hz) γ-bands**	**Mean**	**SEM**	**N**	**p value** (Two – tailed unpaired Student's t – test)
Ctrl	-4.76	1.45	13	Ctrl vs. Mosaic p = 0.895 (n.s.)
Mosaic	-3.90	1.04	8	
