# Supplementary figures and images for: Neuronal network activity and connectivity are impaired in a conditional knockout mouse model with PCDH19 mosaic expression
Source: Mol Psychiatry. 2023 Mar 30;29(6):1710–25. doi: 10.1038/s41380-023-02022-1 (PMC11371655; doi:10.1038/s41380-023-02022-1)

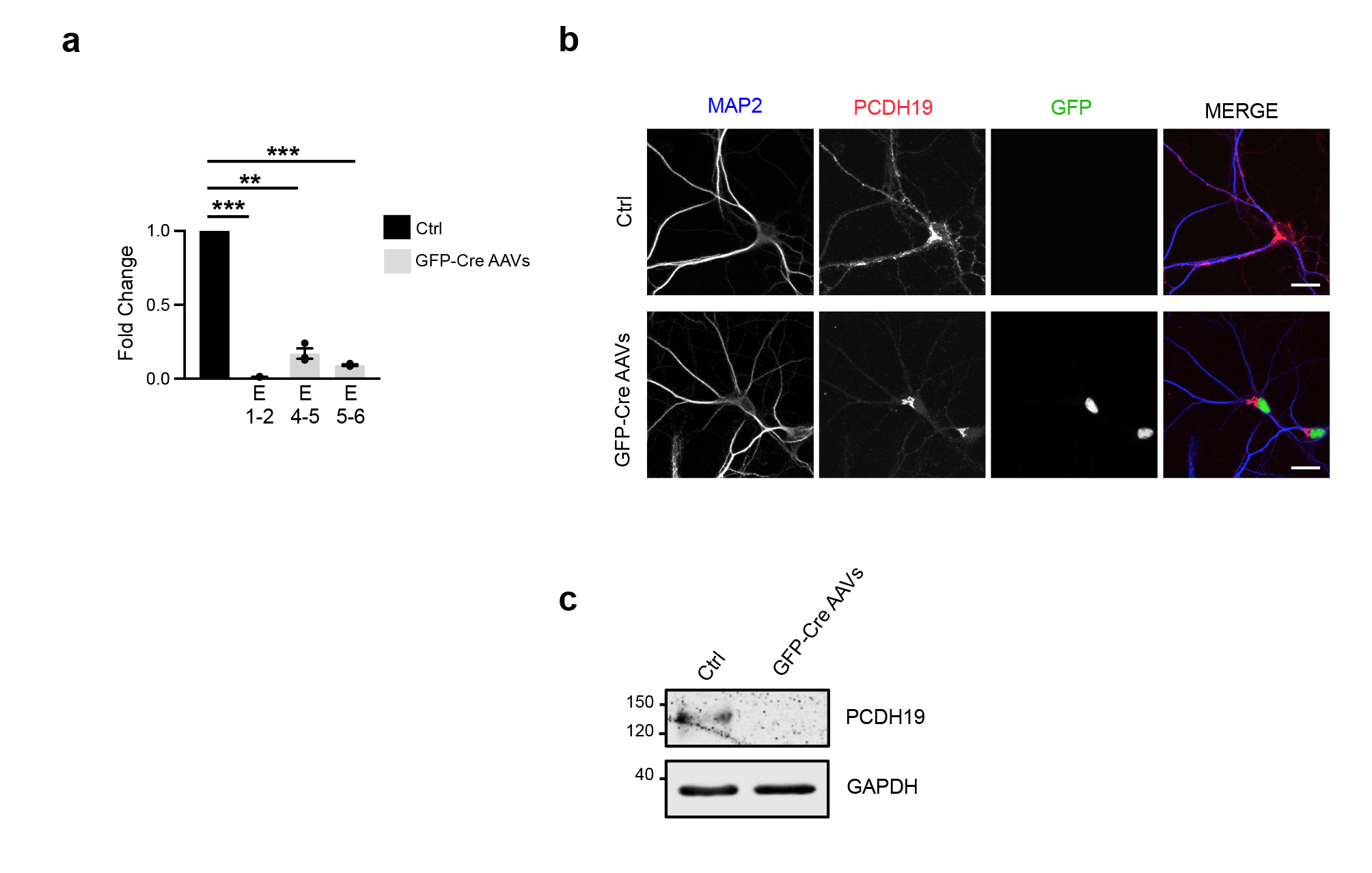

Supplement: Supplementary file 2 — Supplementary Figure 1 [file 41380_2023_2022_MOESM2_ESM.tif]

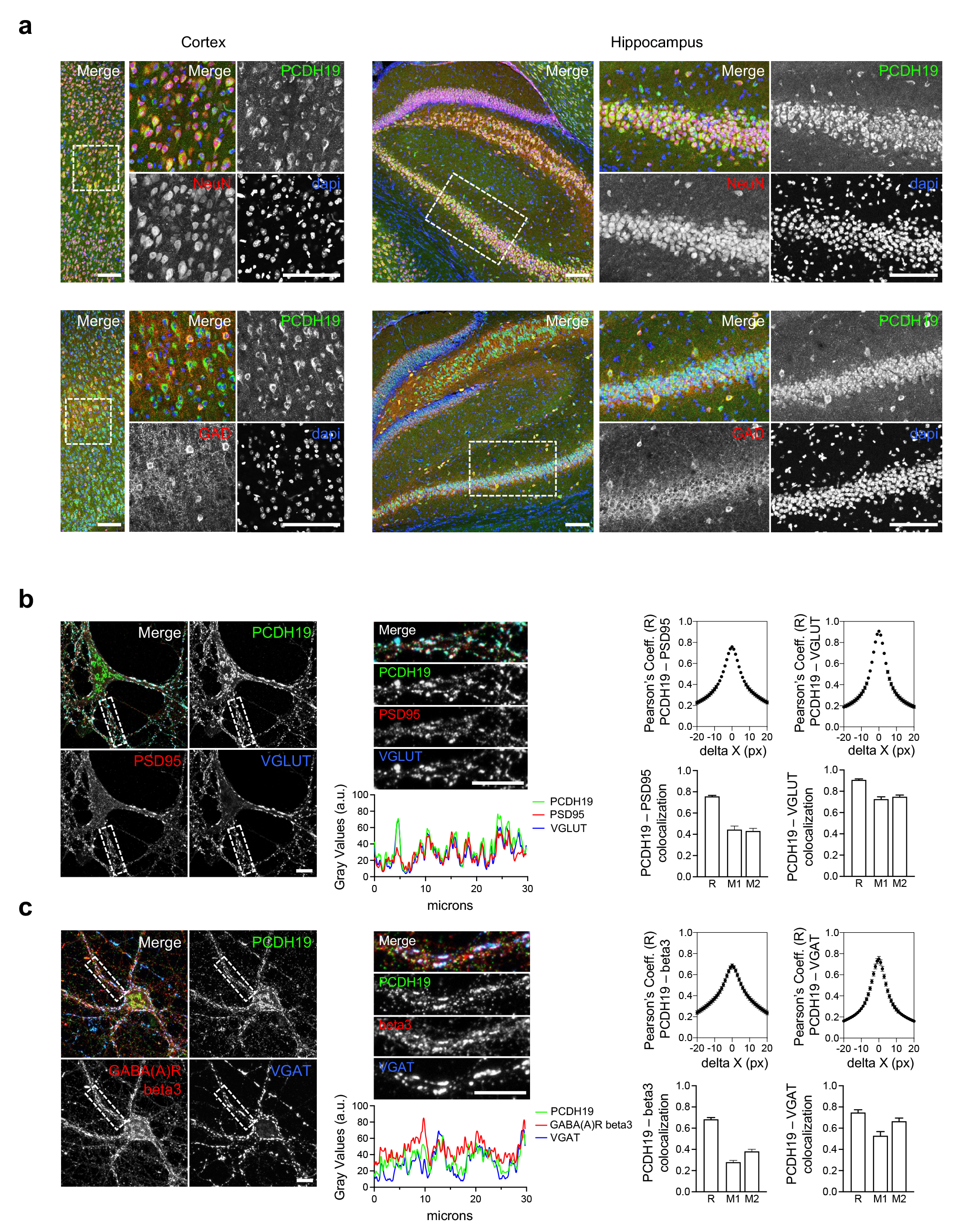

Supplement: Supplementary file 3 — Supplementary Figure 2 [file 41380_2023_2022_MOESM3_ESM.tif]

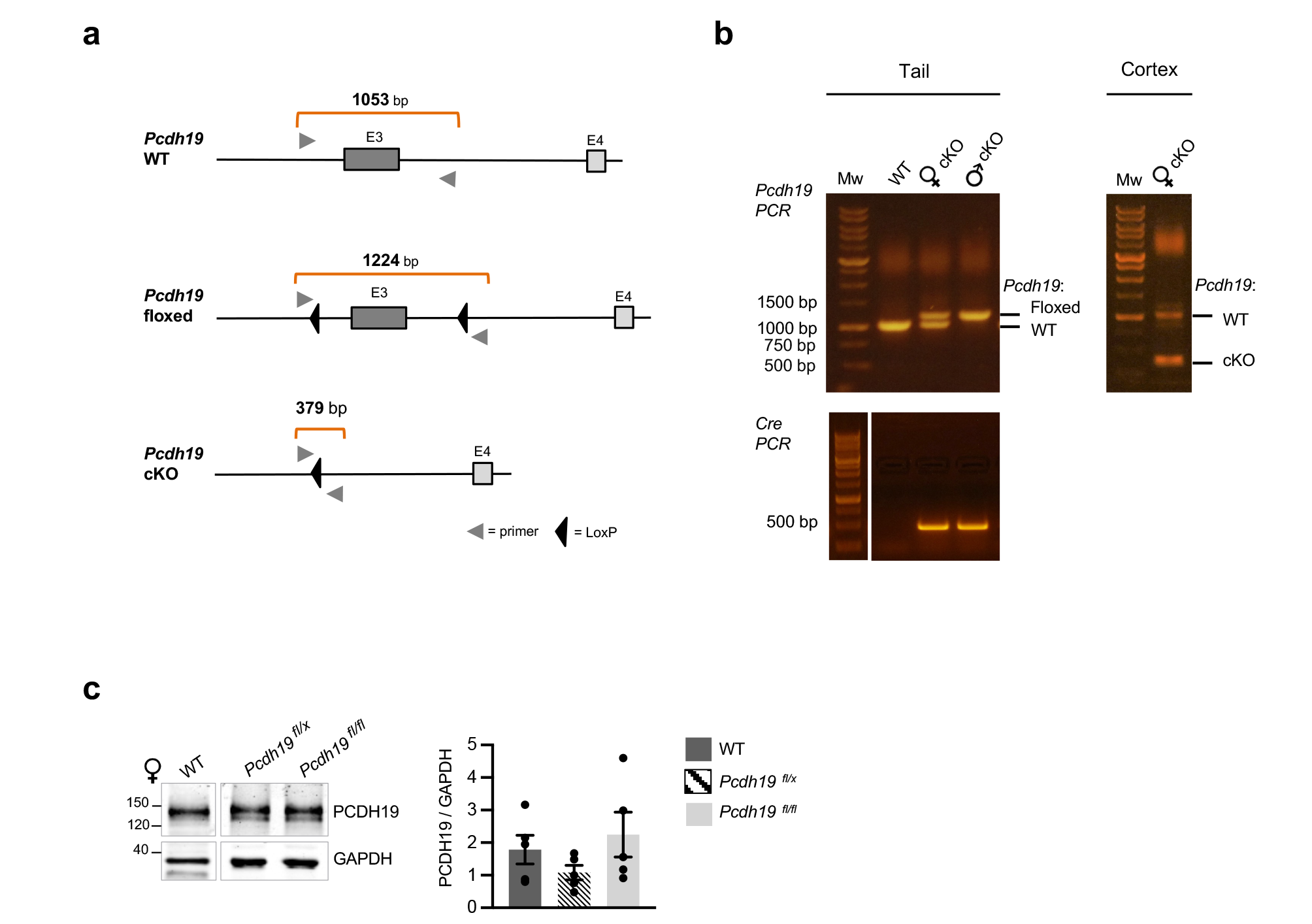

Supplement: Supplementary file 4 — Supplementary Figure 3 [file 41380_2023_2022_MOESM4_ESM.tif]

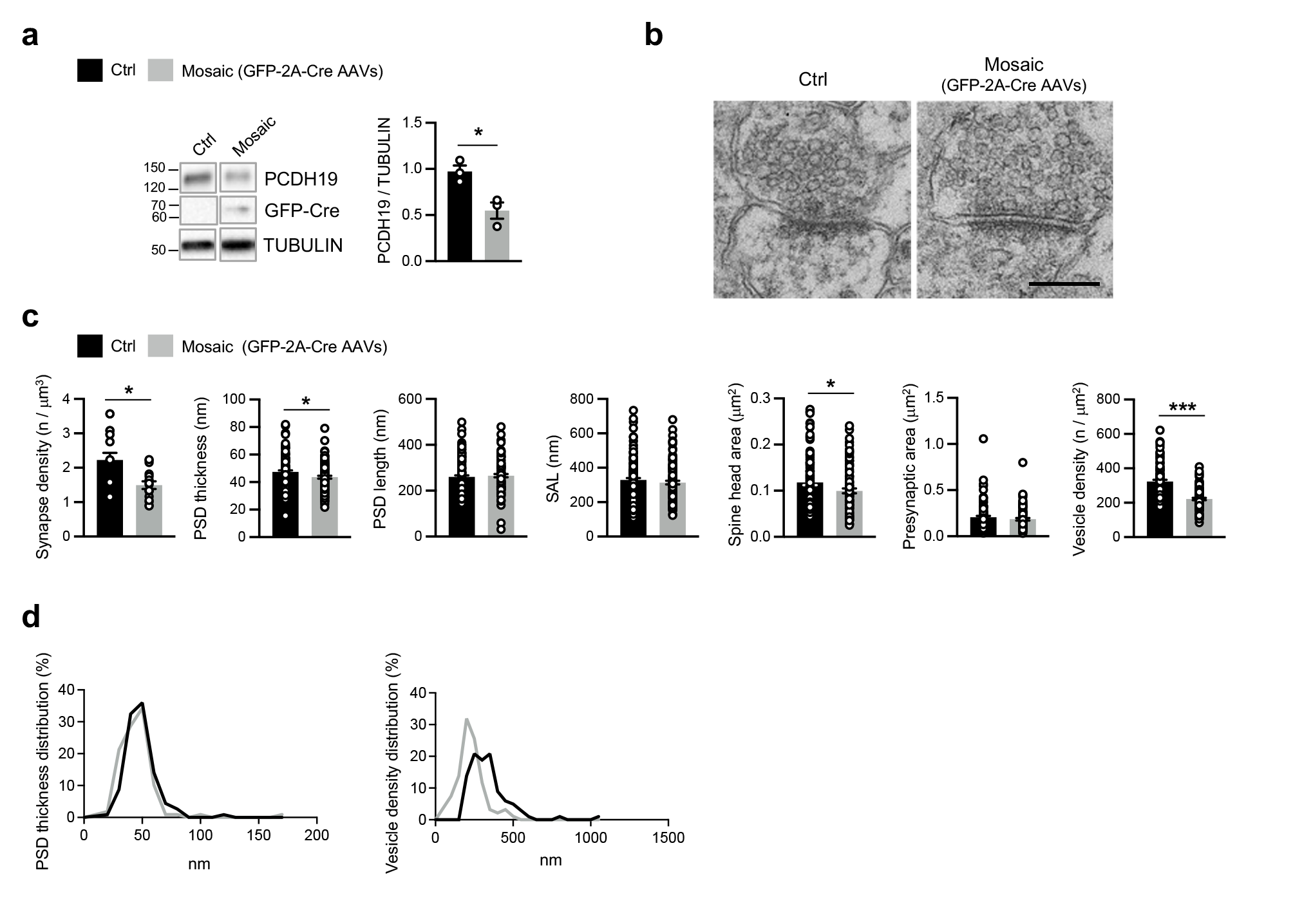

Supplement: Supplementary file 5 — Supplementary Figure 4 [file 41380_2023_2022_MOESM5_ESM.tif]

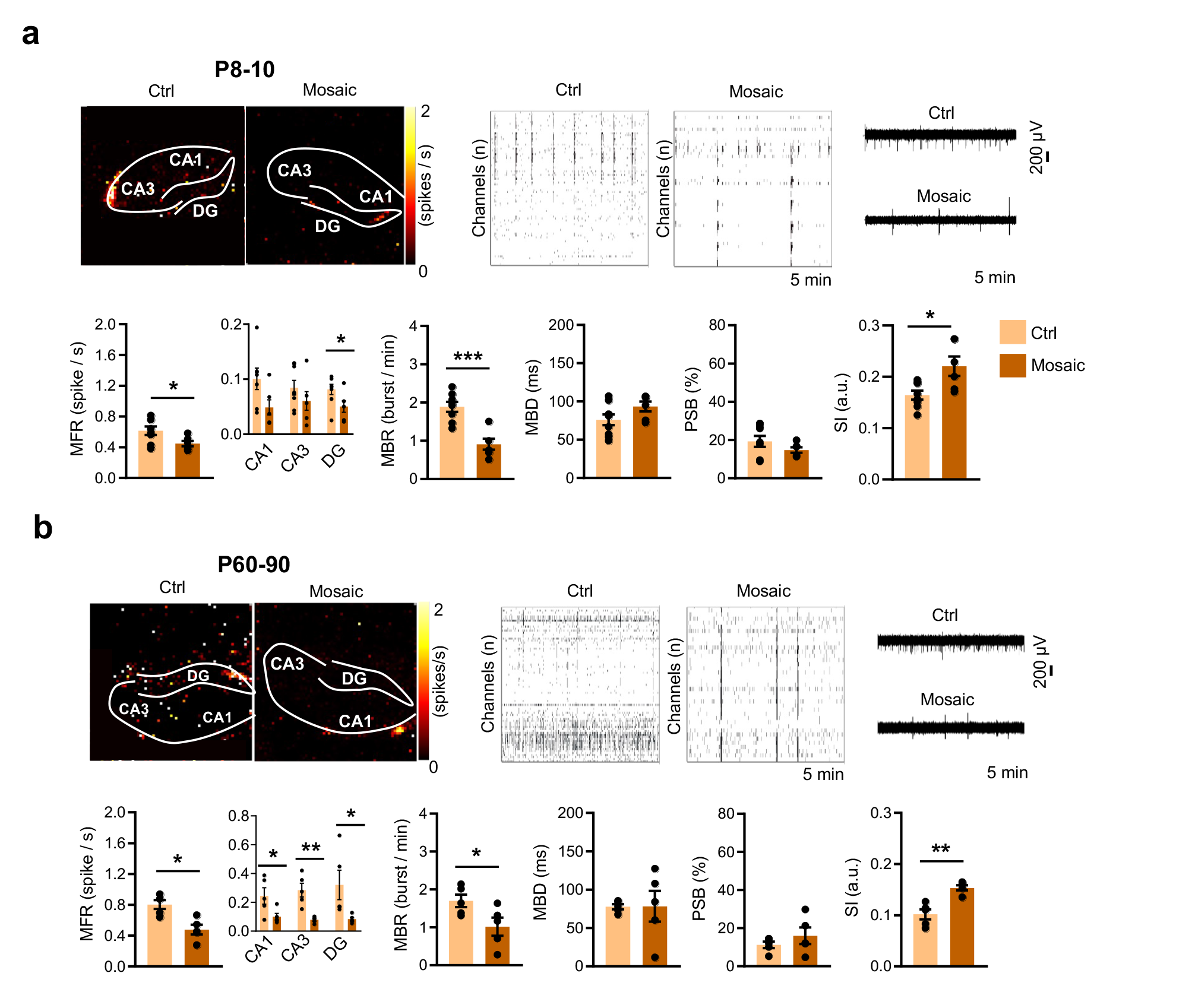

Supplement: Supplementary file 6 — Supplementary Figure 5 [file 41380_2023_2022_MOESM6_ESM.tif]

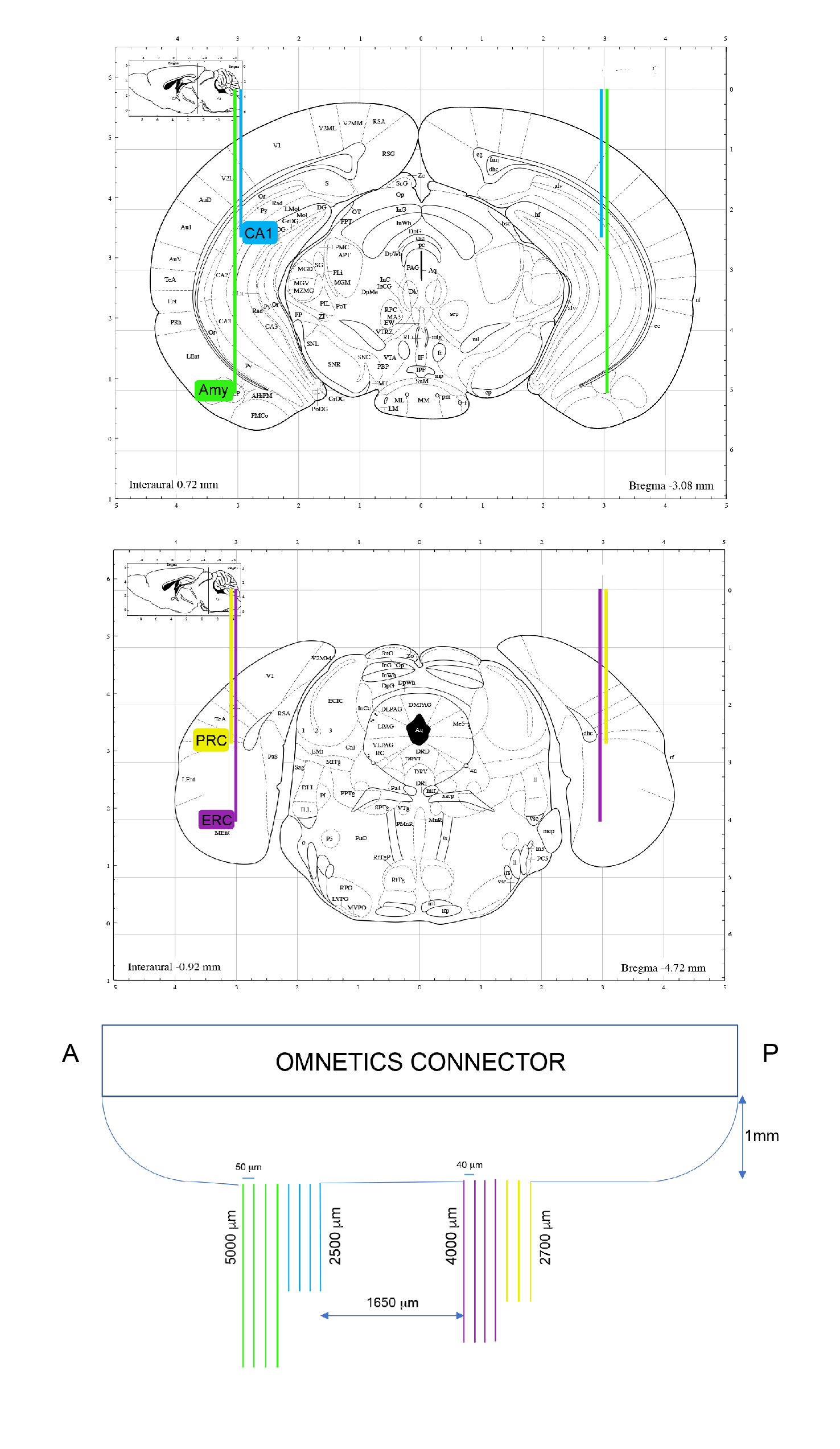

Supplement: Supplementary file 7 — Supplementary Figure 6 [file 41380_2023_2022_MOESM7_ESM.tif]

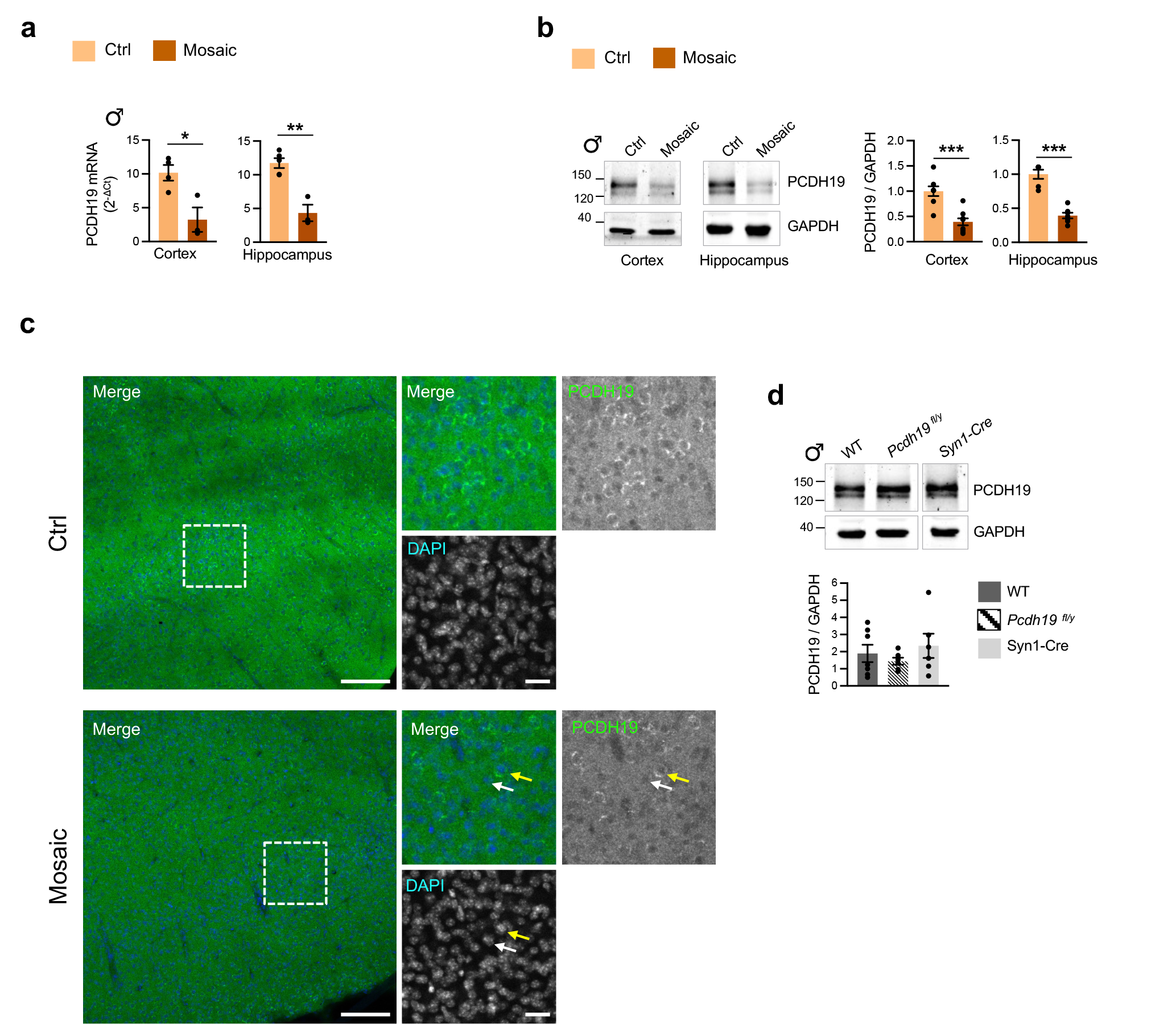

Supplement: Supplementary file 8 — Supplementary Figure 7 [file 41380_2023_2022_MOESM8_ESM.tif]

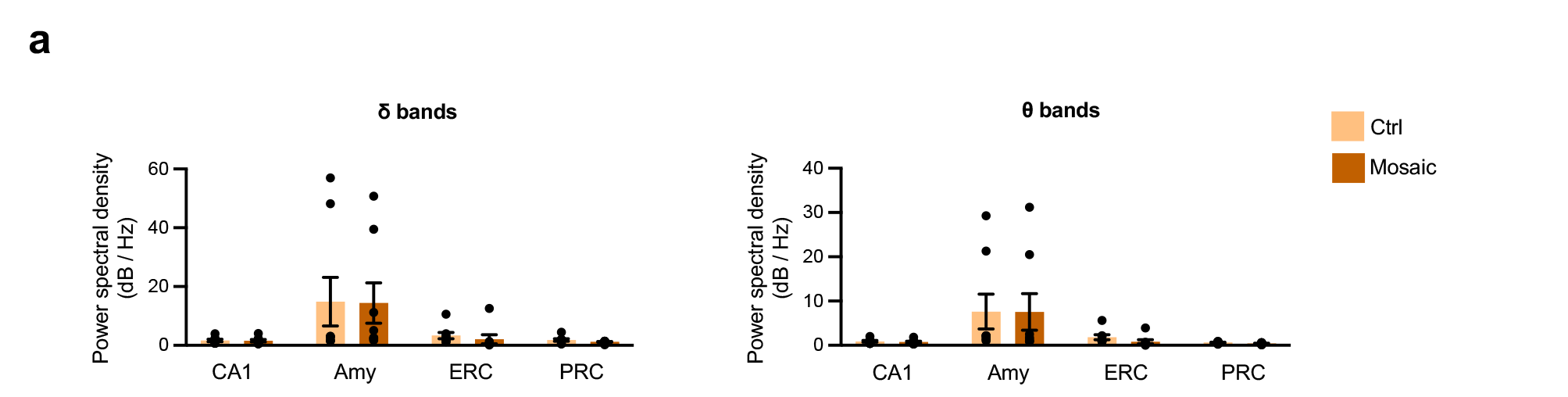

Supplement: Supplementary file 9 — Supplementary Figure 8 [file 41380_2023_2022_MOESM9_ESM.tif]

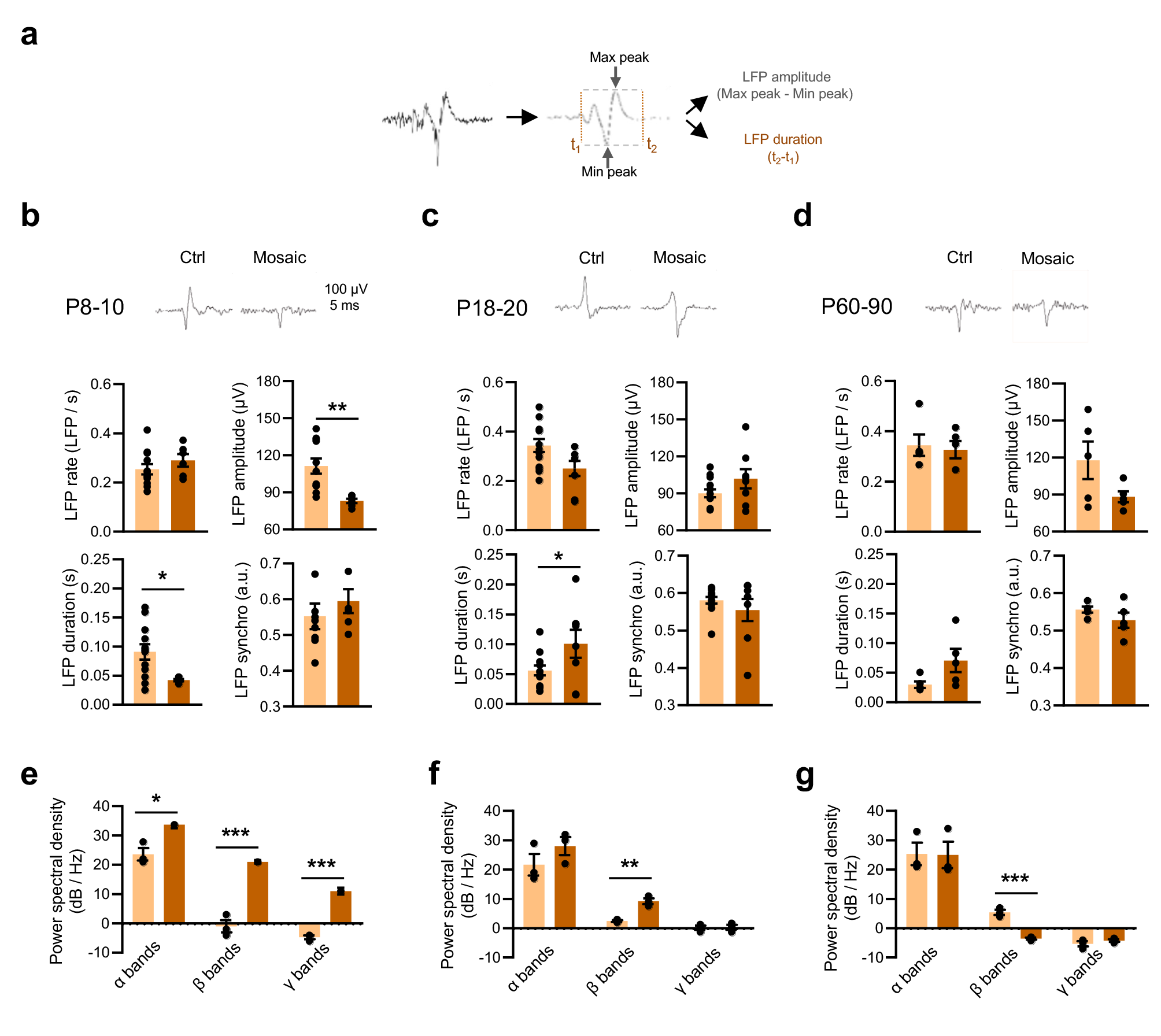

Supplement: Supplementary file 10 — Supplementary Figure 9 [file 41380_2023_2022_MOESM10_ESM.tif]
